# Author Correction: Pro-ferroptotic signaling promotes arterial aging via vascular smooth muscle cell senescence

**DOI:** 10.1038/s41467-024-54088-2

**Published:** 2024-11-14

**Authors:** Di-Yang Sun, Wen-Bin Wu, Jian-Jin Wu, Yu Shi, Jia-Jun Xu, Shen-Xi Ouyang, Chen Chi, Yi Shi, Qing-Xin Ji, Jin-Hao Miao, Jiang-Tao Fu, Jie Tong, Ping-Ping Zhang, Jia-Bao Zhang, Zhi-Yong Li, Le-Feng Qu, Fu-Ming Shen, Dong-Jie Li, Pei Wang

**Affiliations:** 1 The Center for Basic Research and Innovation of Medicine and Pharmacy (MOE), School of Pharmacy, Second Military Medical University/Naval Medical University, Shanghai, China; 2 Jinling Hospital, Affiliated Hospital of Medical School, Nanjing University, Nanjing, China; 3 Department of Vascular and Endovascular Surgery, Changzheng Hospital, Naval Medical University/Second Military Medical University, Shanghai, China; 4 Department of Pharmacy, Shanghai Tenth People’s Hospital, Tongji University School of Medicine, Shanghai, China; 5 Department of Diving and Hyperbaric Medicine, Naval Special Medical Center, Naval Medical University/Second Military Medical University, Shanghai, China; 6 Department of Cardiology, School of Medicine, Shanghai Tenth People’s Hospital, Tongji University School of Medicine, Shanghai, China; 7 Shanghai Key Laboratory of Organ Transplantation, Fudan University, Shanghai, China; 8 Institute of Clinical Science, Zhongshan Hospital Fudan University, Shanghai, China; 9 Department of Orthopedic Surgery/Spine Center, Changzheng Hospital Affiliated Hospital of Naval Medical University/Second Military Medical University, Shanghai, China; 10 The National Demonstration Center for Experimental Pharmaceutical Education, Naval Medical University/Second Military Medical University, Shanghai, China

**Keywords:** Arterial stiffening, Cell death, Iron, Ageing

Correction to: *Nature Communications* 10.1038/s41467-024-45823-w, published online 16 February 2024

The original version of this Article contained an error in Fig. 1b. The tubulin loading control from Fig. 5e was inadvertently included as the loading control during figure assembly. This panel has been replaced with the correct tubulin control. The Source Data have been updated with the correct Western blot. This has been corrected in both the PDF and HTML versions of the Article.

## Source data

Updated Source data

