# Supplementary material for: Pro-ferroptotic signaling promotes arterial aging via vascular smooth muscle cell senescence
Source: Nat Commun. 2024 Feb 16;15:1429. doi: 10.1038/s41467-024-45823-w (PMC10873425; doi:10.1038/s41467-024-45823-w)
Supplement: Supplementary file 1 — Supplementary Information [file 41467_2024_45823_MOESM1_ESM.pdf]

Supplemental Figure 1

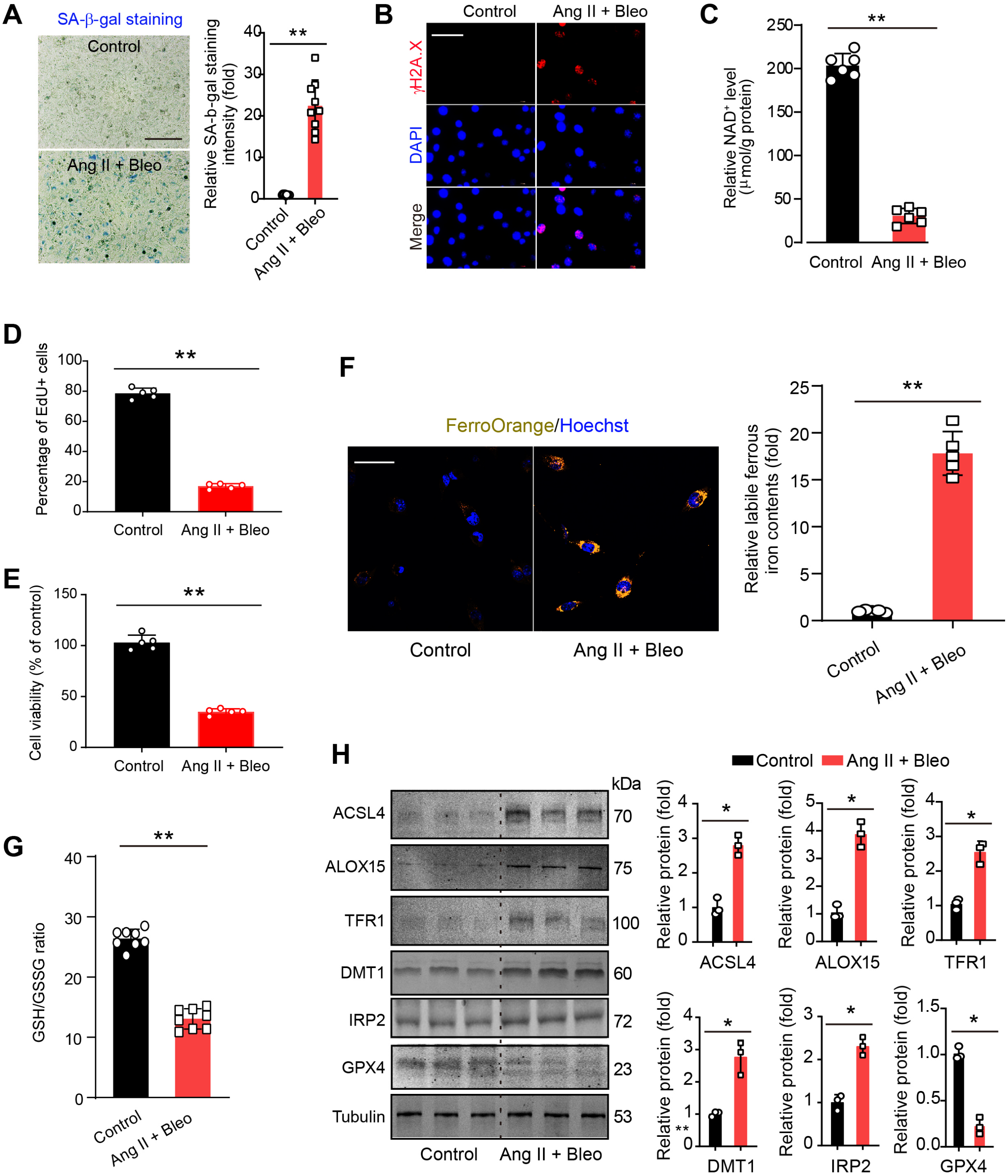

Supplemental Figure 1. Senescence, NAD$^+$ loss and ferroptosis stress are remarkably induced in VSMCs and mouse aortae upon combined stresses of angiotensin II and bleomycin.

(A) Representative images and quantitative analysis of SA-β-gal activity in mouse VSMCs (MOVAS cell line) treated with PBS (control) or angiotensin II (Ang II) + bleomycin (Bleo) for 5 days. n = 9 biologically independent samples.

(**B**) Fluorescent immunocytochemistry of senescence marker γH2A.X in mouse VSMCs treated with PBS or Ang II+Bleo for 5 days**.** The fixed cells were stained by anti-γH2A.X and followed by Alexa Fluor 555-congugated secondary antibody. Nuclei were stained by DAPI. Scale bar, 100 μm. n = 6 biologically independent samples.

(C) Intracellular levels of NAD^+ in mouse VSMCs treated with PBS or Ang II+Bleo for 5 days.

(**D**) Cell proliferation was measured by evaluating EdU incorporation. Mouse VSMCs treated with PBS or Ang II+Bleo for 5 days and labeled with EdU (Click-iT EdU, Invitrogen) for 1 h and processed to detect incorporated EdU. Images were obtained by fluorescent microscopy, and the number of EdU-positive nuclei was counted. n = 6 biologically independent samples.

(**E**) Cell viability was measured using CCK-8 assay. Mouse VSMCs treated with PBS or Ang II+Bleo for 5 days and cultured in CCK-8-added medium for 1 hour. Th absorption at 450 nm was recorded using microplate reader. n = 6 biologically independent samples.

(**F**) FerroOrange probe showing the intracellular ferrous iron pool in mouse VSMCs treated with PBS or Ang II+Bleo for 5 days. n = 5 biologically independent samples. Scale bar, 100 μm.

(**G**) Intracellular GSH/GSSG ratio in mouse VSMCs treated with PBS or Ang II+Bleo for 5 days. n = 8 biologically independent samples.

(**H**) Immunoblotting analyses of pro-ferroptosis factors ACSL4, ALOX15, TFR1 and anti-ferroptosis factor GPX4 in mouse VSMCs treated with PBS or Ang II+Bleo for 5 days. Tubulin was used as a loading control. n = 3 biologically independent samples.

Data expressed the mean±SEM. **P*<0.05, ***P*<0.01. Comparisons of parameters were performed with Two-sided Unpaired t-test. Ang II, angiotensin II; Bleo, bleomycin; GPX4, glutathione peroxidase 4; ACSL4, acyl-CoA synthetase long-chain family member 4; ALOX15, arachidonate 15-lipoxygenase; TFR1, transferrin receptor; MDA, malondialdehyde. Doses used for cell culture: Ang II, 0.1 μM; Bleo, 100 nM.

Supplemental Figure 2

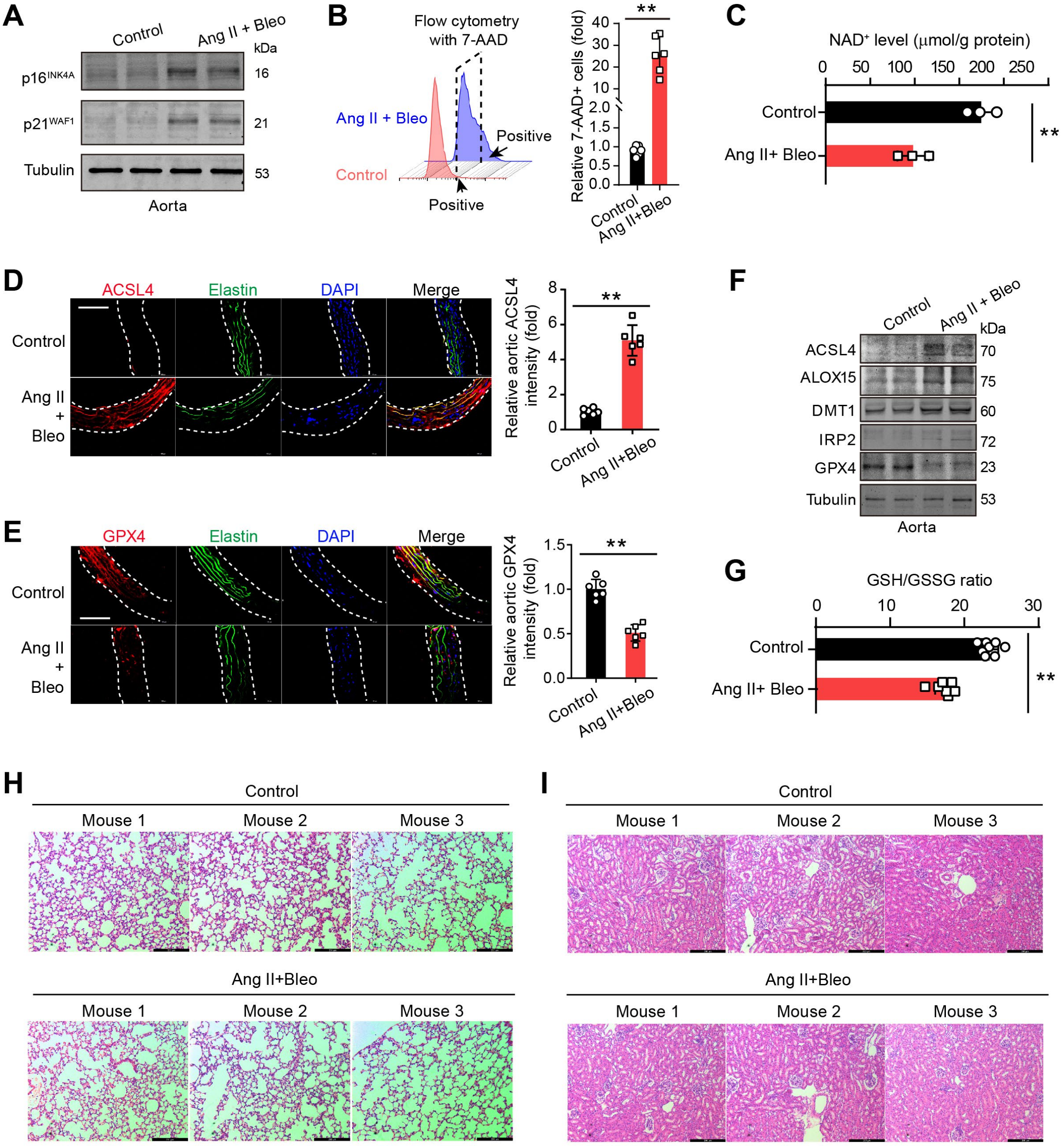

Supplemental Figure 2. Establishment of experimental vascular senescence model by infusion Ang II plus bleomycin via ALZET Osmotic micropump.

(**A**) Representative immunoblotting of senescence markers $p16^{INK4A}$ and $p21^{WAF1}$ in aortae from mice infused with angiotensin II (Ang II, 400 ng/kg/min) plus bleomycin (Bleo, 40 ng/kg/min) via ALZET Osmotic micropumps for 2 weeks. The mice in control group were infused with saline with same volume in osmotic pumps. Tubulin was used as a loading control. Experiments were repeated at least for three times.

(B) Flow cytometer analysis of dead cells with 7-AAD probe in aortae from mice infused with saline (control) and Ang II+Bleo via ALZET osmotic pumps. n = 6 biologically independent samples.

(**C**) NAD^+ level in aortae of mice infused with saline (control) or Ang II+Bleo. n = 3 biologically independent samples.

(**D-E**) Fluorescent immunohistochemistry showing the expression of ACSL4 **(D)** and GPX4 **(E)** in aortae of mice infused with saline (control) or Ang II+Bleo**.** Sections were stained by anti-ACSL4 or anti-GPX4 and followed by Alexa Fluor 555-congugated secondary antibodies. Elastin was visualized by autofluorescence and nuclei were stained by DAPI. Scale bar, 100 μm. n = 6 biologically independent samples.
(**F**) Representative immunoblotting of pro-ferroptosis factors (ACSL4, ALOX15, DMT1 and IRP2) and anti-ferroptosis factor (GPX4) in aortae from mice infused with saline (control) and Ang II+Bleo via ALZET osmotic pumps. Tubulin was used as a loading control. Experiments were repeated for three times.
(**G**) GSH/GSSG ratio in aortae of mice infused with saline (control) or Ang II+Bleo. n = 8 biologically independent samples.
(H) Representative images of H & E staining in lung of the mice with Ang II+Bleo. Scale bar, 200 μm. Experiments were repeated at least for three times.
(I) Representative images of H & E staining in kidney of the mice with Ang II+Bleo. Scale bar, 200 μm. Experiments were repeated at least for three times.
Data expressed the mean±SEM. $^{**}P<0.01$. Comparisons of parameters were performed with Two-sided Unpaired t-test. Ang II, angiotensin II; Bleo, bleomycin; GPX4, glutathione peroxidase 4; ACSL4, acyl-CoA synthetase long-chain family member 4; ALOX15, arachidonate 15-lipoxygenase; Doses used for osmotic pump infusion: Ang II, 400 ng/kg/min; Bleo, 40 ng/kg/min.

Supplemental Figure 3

Fig. S3

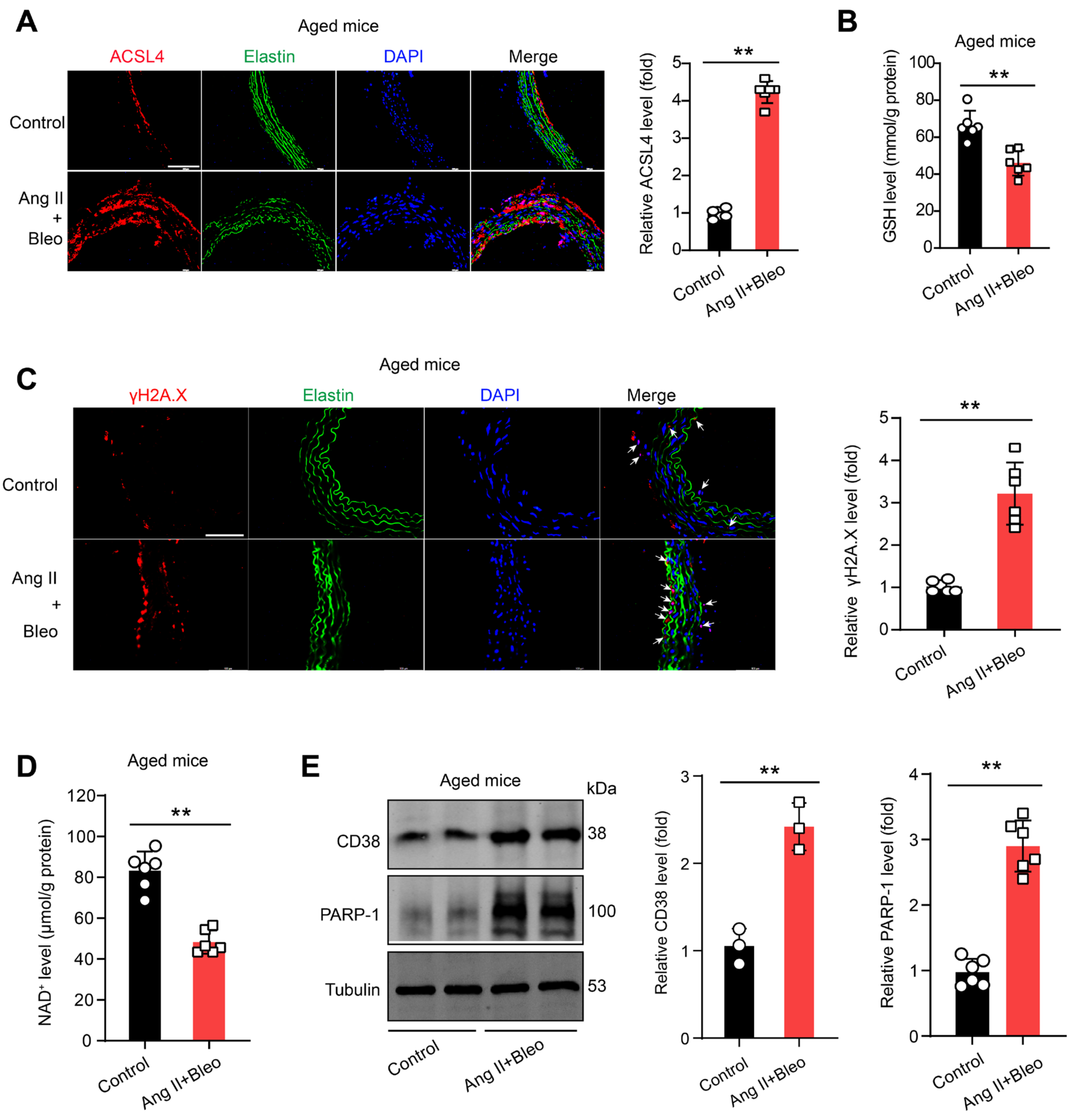

Supplemental Figure 3. Treatment of angiotensin II (Ang II) plus bleomycin (Bleo) promotes ferroptosis stress, senescence and NAD$^+$ loss in aged mice.

(A) Fluorescent immunocytochemistry and quantitative analysis of ferroptosis marker ACSL4 in aortae of mouse infused with saline (Control) or Ang II+Bleo for 2 weeks. Nuclei were stained by DAPI. Scale bar, 100 μm. n = 6 biologically independent samples.

(**B**) GSH contents in aortae of mouse infused with saline (Control) or Ang II+Bleo for 2 weeks. n = 6 biologically independent samples.

(C) Fluorescent immunocytochemistry and quantitative analysis of senescence marker H2A.X in aortae of mouse infused with saline (Control) or Ang II+Bleo for 2 weeks. Nuclei were stained by DAPI. H2A.X colocalizes with DAPI (nuclei). Scale bar, 100 μm. n = 6 biologically independent samples.

(**D**) Levels NAD$^+$ in aortae of mouse infused with saline (Control) or Ang II+Bleo for 2 weeks.

n = 6 biologically independent samples.
(**E**) Immunoblotting analysis of two NAD^+ consumers CD38 and PARP-1 in aortae of mouse infused with saline (Control) or Ang II+Bleo for 2 weeks**.** n = 6 biologically independent samples.
Data expressed the mean±SEM. $^{**}P<0.01$. Comparisons of parameters were performed with Two-sided Unpaired t-test.

Supplemental Figure 4

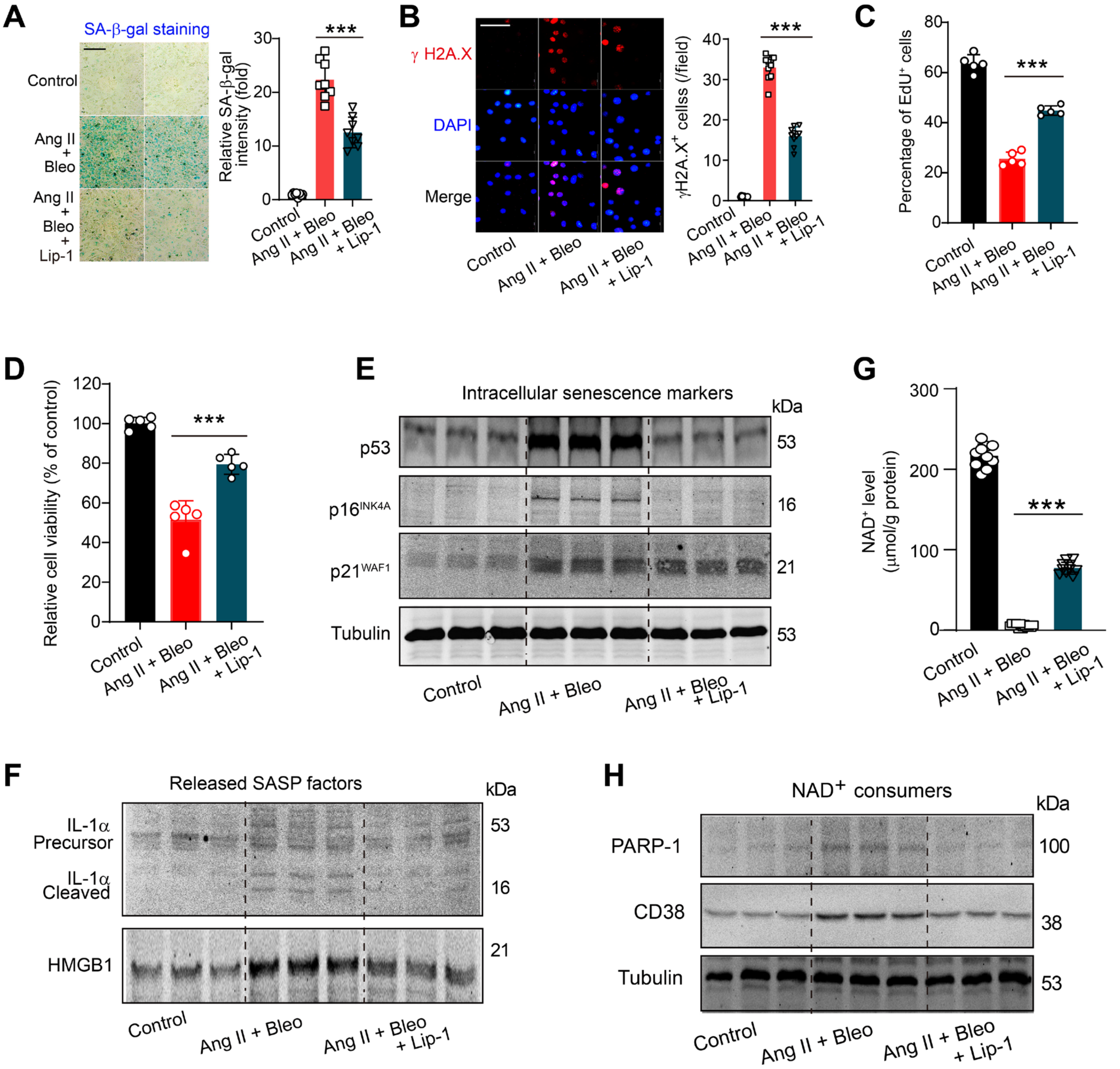

Supplemental Figure 4. Ferroptosis inhibitor liproxstain-1 attenuates oxidative stress, NAD$^+$ loss and senescence in cultured VSMCs.

(**A**) Representative SA-β–gal staining and quantitative analysis in VSMCs. Scale bar, 200 μm. n = 8 biologically independent samples.

(**B**) Representative fluorescent immunohistochemistry images and quantitative analysis of γH2A.X in mouse VSMCs. VSMCs were stained by primary antibody against γH2A.X followed with Alexa Fluor 555-conjugated second antibody (Red). DAPI was used to stain nuclei (Blue). The nuclear staining of γH2A.X colocalized with DAPI. Scale bar, 100 μm. n = 10 biologically independent samples.

(**C**) Cell proliferation was measured by evaluating EdU incorporation assay. n = 5 biologically independent samples.

(**D**) Cell viability was measured by evaluating CCK-8 assay.

(E) Immunoblotting analysis of senescence molecular markers p53, p16^{INK4A} and p21^{WAF1} in VSMCs. Experiments were repeated for three times.

(**F**) Immunoblotting analysis of released SASP factors (IL-1α and HMGB1) into culture medium. Experiments were repeated for three times.

(**G**) Intracellular NAD^+ level in VSMCs. n = 10 biologically independent samples.
(**H**) Protein expressions of two NAD^+ consumers (PARP-1 and CD38) in VSMCs were evaluated with immunoblotting analysis. Experiments were repeated for three times.
Comparisons of parameters were performed with One-Way ANOVA followed by a Tukey's multiple comparisons test. $^{**}P<0.01$, $^{***}P<0.001$. Ang II, angiotensin II; Bleo, bleomycin; γH2A.X, phosphorylated $H2A.X^{Ser139}$; GPX4, glutathione peroxidase 4; IL-1α, interleukin-1α; OE, overexpression; NAD^+, nicotinamide adenine dinucleotide; GSH, glutathione; HMGB1, high mobility group box 1; PARP-1, poly-ADP-ribose polymerase-1; SA-β-gal, senescence-associated beta galactosidase. Ang II, 0.1 μM; Bleo, 100 nM; liproxstatin-1, 1 μM.

Supplemental Figure 5

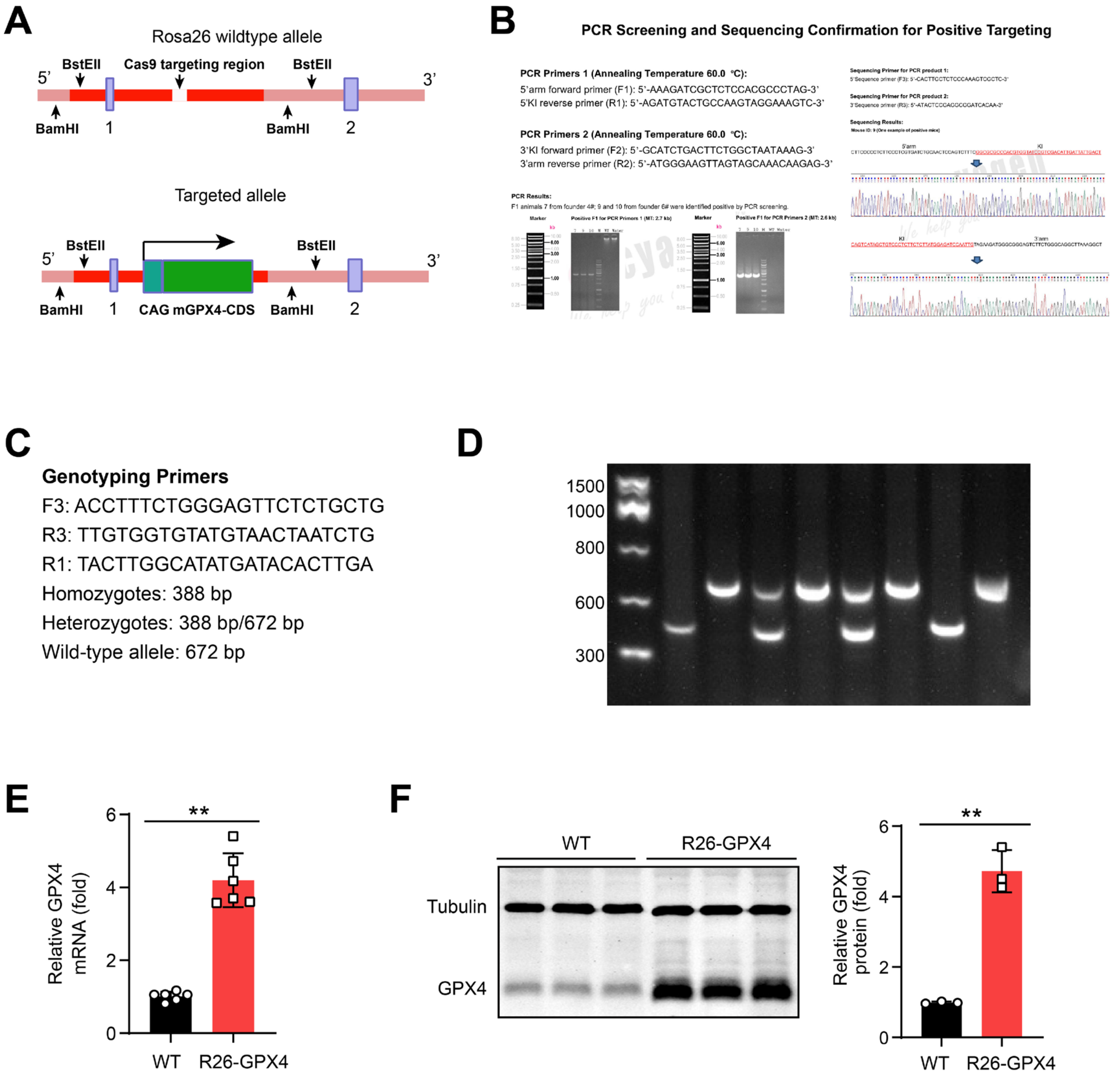

Supplemental Figure 5. Generation of a mouse line with GPX4 knockin using CRISPR/Cas9 strategy.

(**A**) Strategy for gene targeting of ROSA26-GPX4 mice. The mouse GPX4 CDS driven by CAG promotor was inserted into ROSA26 site.

(**B**) The primers for PCR screening, PCR screening results and sequencing confirmation for the positive targeting of GPX4 into ROSA26 allele (R26-GPX4 mice).

(**C**) Genotyping primers and PCR results for R26-GPX4 mice breeding.

(**D**) Representative agarose gel electrophoresis of genotyping in WT and R26-GPX4 mice.

(E) Quantitative real-time PCR showing the GPX4 mRNA levels in aortae from WT and R26-GPX4 mice. n = 6 biologically independent samples.

(**F**) Representative immunoblotting images showing the GPX4 protein levels in aortae from WT and R26-GPX4 mice. Tubulin was used as a loading control. n = 3 biologically independent samples.

Data expressed the mean±SEM. **P<0.01. Comparisons of parameters were performed with Two-sided Unpaired t-test.

Supplemental Figure 6

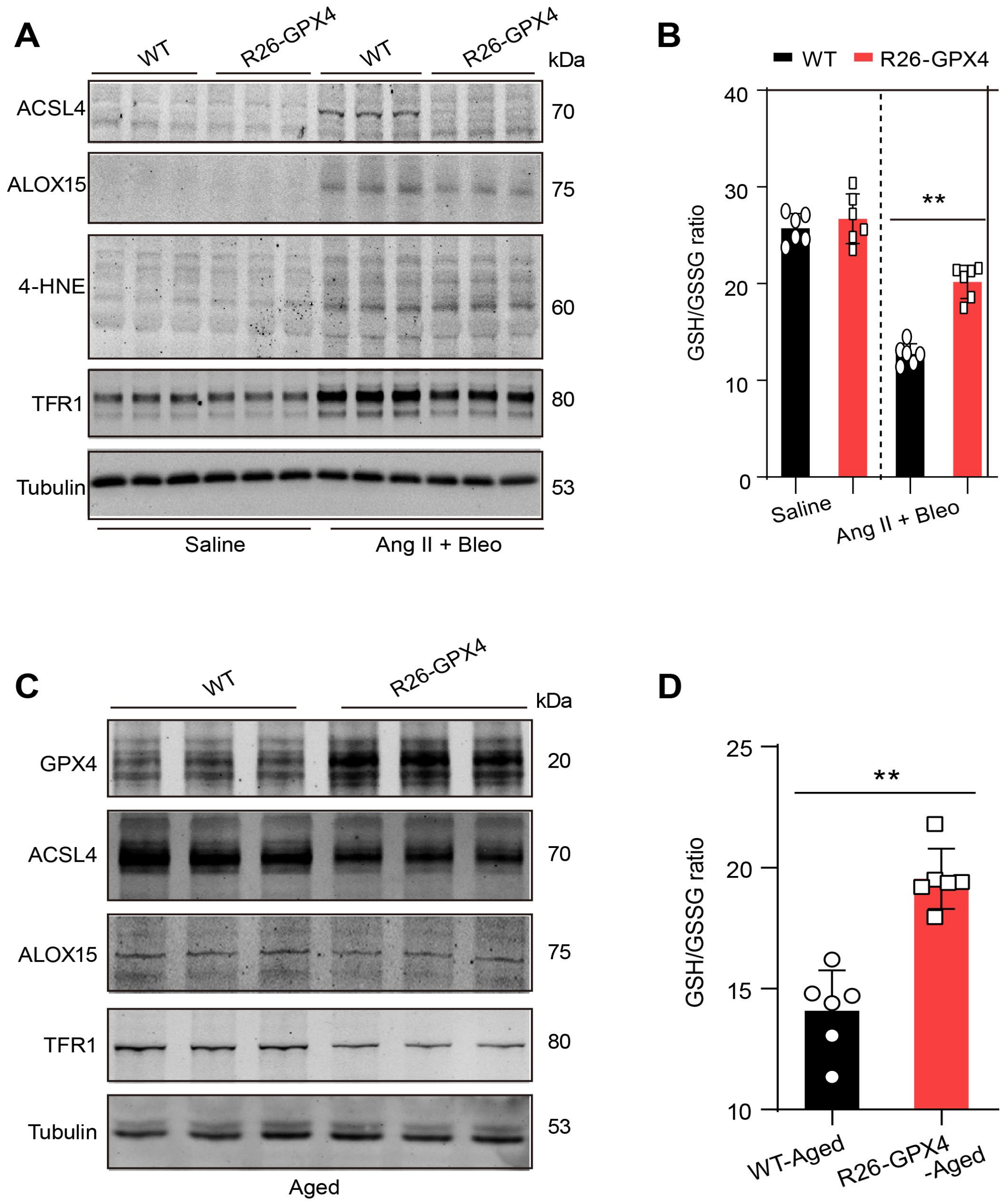

Supplemental Figure 6. Knockin of GPX4 ameliorates ferroptosis stress in mice with experimental vascular senescence via infusion of Ang II + bleomycin and natural aged mice.

(A) Immunoblotting analysis of pro-ferroptosis factors ACSL4, ALOX15, 4-HNE and TFR1 in aortae from WT and GPX4 knock-in mice infused with saline or Ang II+Bleo. n = 3 biologically independent samples.

(**B**) Intracellular GSH/GSSG ratio in aortae from WT and GPX4 knock-in mice infused with saline or Ang II+Bleo. n = 6 biologically independent samples.

(C) Immunoblotting analysis of ferroptosis markers GPX4, ACSL4, ALOX15 and TFR1 in aortae from aged WT and GPX4 knock-in mice. n = 3 biologically independent samples.

(**D**) Intracellular GSH/GSSG ratio in aortae from aged WT and GPX4 knock-in mice. n = 6 biologically independent samples.

Supplemental Figure 7

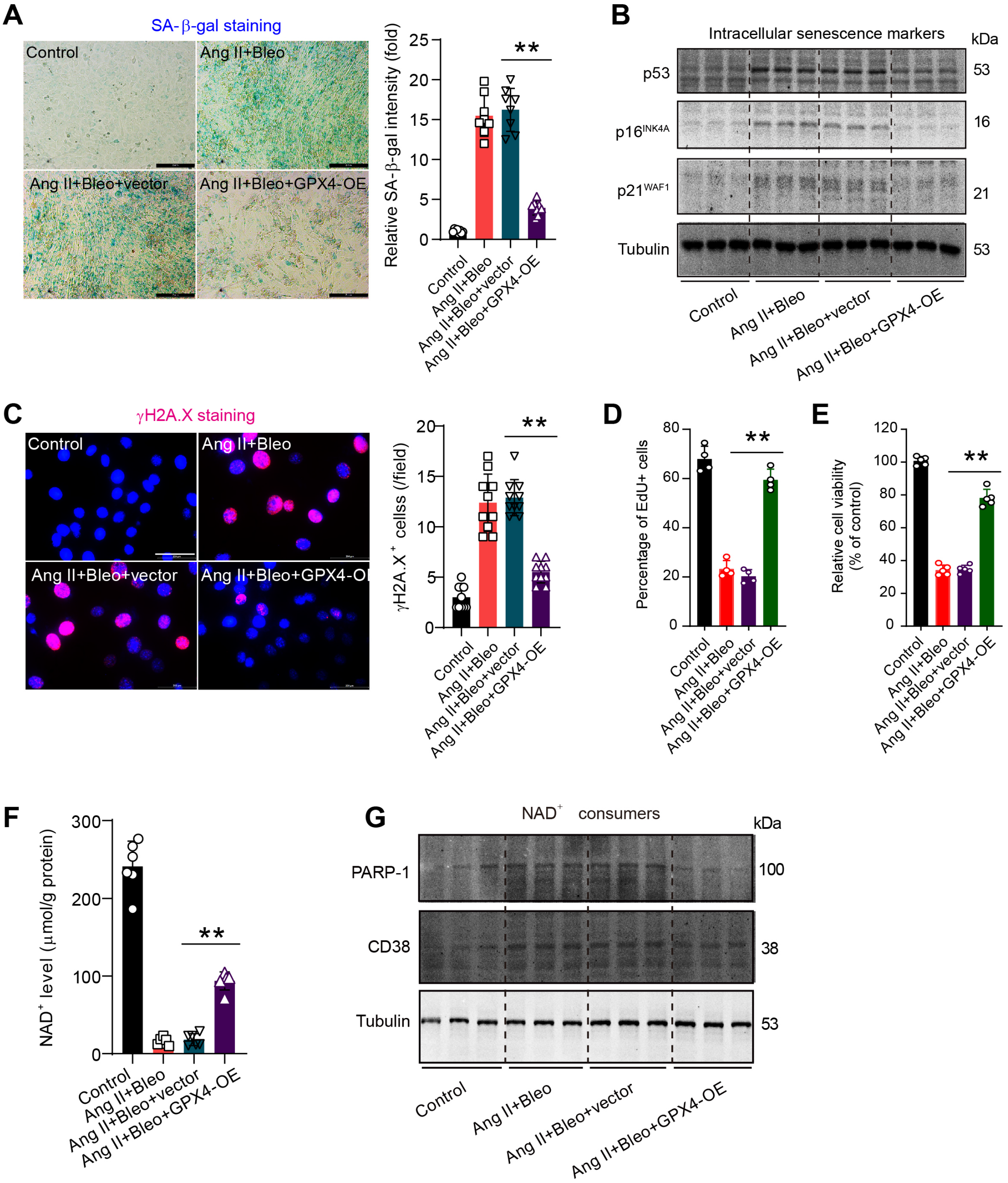

Supplemental Figure 7. Overexpression of GPX4 blocks attenuates senescence and rescues NAD$^+$ loss in cultured VSMCs.

(A) Influence of overexpression of GPX4 on SA-β-gal activity in VSMCs. n = 8 biologically independent samples.

(**B**) Immunoblotting analysis of senescence markers p53, p21^{WAF1} and p16^{INK4A} in VSMCs. n = 3 biologically independent samples.

(**C**) Representative immunofluorescent images and quantitative analysis showing the influence of GPX4 overexpression (OE) on γH2A.X in VSMCs. Scale bar, 100 μm. n = 10 biologically independent samples.

(**D**) Cell proliferation was measured by evaluating EdU incorporation assay in VSMCs. n = 4 biologically independent samples.

(**E**) Cell viability was measured by evaluating CCK-8 assay in VSMCs. n = 5 biologically independent samples.

(**F**) Intracellular NAD^+ and GSH/GSSG ratio in VSMCs. n = 6 biologically independent samples. **(G)** Representative immunoblotting analysis of NAD^+ consumers PARP-1 and CD38 in VSMCs. n = 3 biologically independent samples.

Data expressed the mean±SEM. ***P*<0.01. Comparisons of parameters were performed with One-Way ANOVA followed by a Tukey's multiple comparisons test. Doses used: Ang II, 0.1 μM; Bleo, 100 nM; Lip-1,1 μM.

Supplemental Figure 8

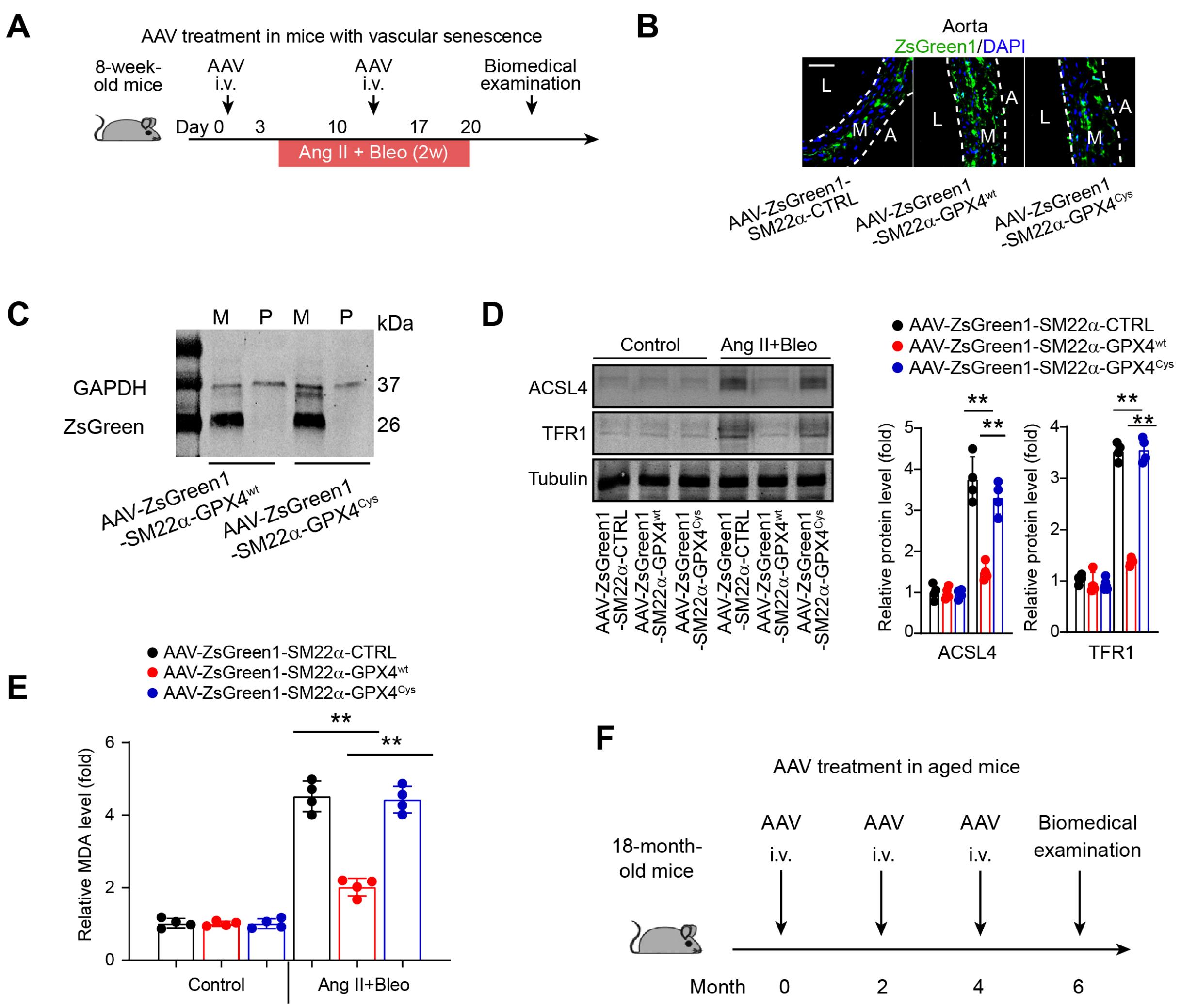

Supplemental Figure 8. VSMC-specific overexpression of GPX4, but not catalytically inactive mutant GPX4Cys, inhibits vascular ferroptosis stress.

(**A**) Study design of AAV treatment in mice with experimental vascular senescence. The AAV particles (3 × 10^{11} vg per mouse, for twice) were injected from tail vein and three days later the mice were infused with Ang II+Bleo to induce vascular senescence. At 20 days later, the mice were sacrificed for examination.

(**B**) Confirmation of VSMC-specific overexpression of GPX4 in mice by ZsGreen1 immunofluorescent analysis. In the immunofluorescence analysis, the green signals (ZsGreen1) were noted in the tunica media of aortae. The GA   The serotype 2/9 of adeno-associated virus (AAV2/9) carrying coding sequences of $GPX4^{wt}$ or $GPX4^{cys}$ under SMC-specific SM22a promoter (AAV-ZsGreen1-SM22α-$GPX4^{wt}$ and AAV-ZsGreen1-SM22α-$GPX4^{Cys}$) was injected. The control AAV (AAV-ZsGreen1-SM22α-CTRL) was also constructed. ZsGreen1 is a fluorescent protein used for tracing. Scale bar, 50 μm. Experiments were repeated for three times.

(**C**) Confirmation of VSMC-specific overexpression of GPX4 in mice by immunoblotting analysis. In immunoblotting analysis, the tunica media (M) and perivascular adipose tissue (P) were isolated respectively for immunoblotting analysis. Experiments were repeated for three times.

(**D**) Immunoblotting analyses of ferroptosis markers ACSL4 and TFR1 in aortae of mice were

infused with Ang II+Bleo and with AAV injection. n = 4 biologically independent samples.
(**E**) Relative MDA contents in aortae of mice were infused with Ang II+Bleo and with AAV injection. n = 4 biologically independent samples.
(**F**) Study design of AAV treatment in aged mice. The AAV particles were injected for three times (every two months, 3 × 10^{11} vg per mouse) in 18-month-old mice. At 6 months later (three injections), the mice were sacrificed for examination of vascular aging.
Data expressed the mean±SEM. **P<0.01. Comparisons of parameters were performed with One-Way ANOVA followed by a Tukey's multiple comparisons test.

Supplemental Figure 9

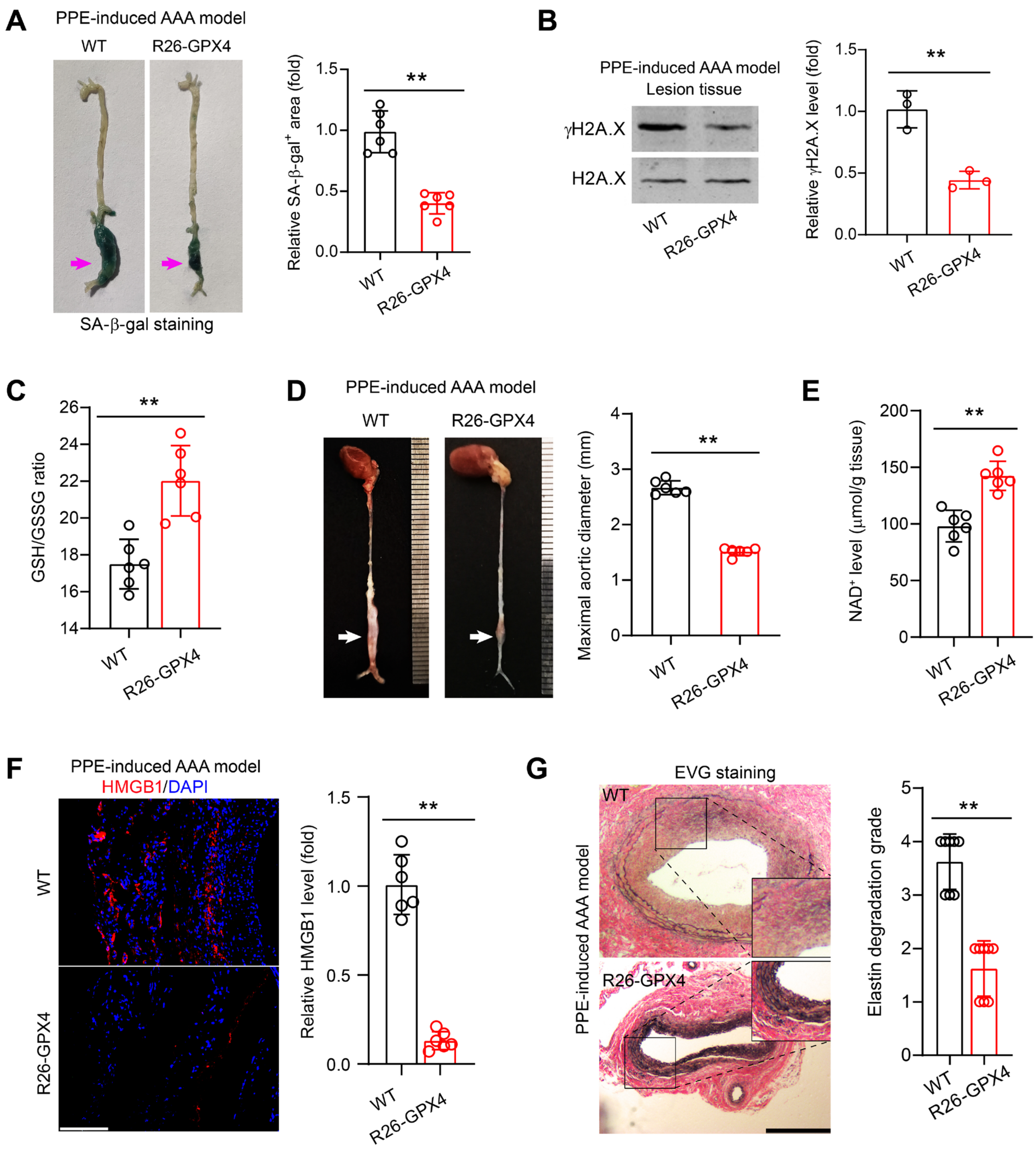

Supplemental Figure 9. GPX4 knockin mice exhibit reduced senescence and pathologies in an experimental model of abdominal aortic aneurysm.

(**A**) Representative image and quantitative analysis on the SA-β-gal staining in aortae tree from WT and R26-GPX4 mice with experimental AAA induced by porcine pancreatic elastase. SA-β-gal staining was performed in the whole aorta tree. n = 6 biologically independent samples.

(**B**) Representative immunoblotting and quantitative analysis of γH2A.X in AAA tissue from WT and R26-GPX4 mice. n = 3 biologically independent samples.

(**C**) GSH/GSSG ratio in AAA aortae of WT and R26-GPX4 mice. n = 6 biologically independent samples.

(**D**) Representative image and quantitative analysis of AAA diameter of enlarged aorta from WT and R26-GPX4 mice. n = 6 biologically independent samples.

(**E**) NAD^+ content in AAA aortae of WT and R26-GPX4 mice. n = 6 biologically independent samples.

(F) Representative images and quantitative analysis of HMGB1 immunohistochemistry staining in the AAA lesion of WT and R26-GPX4 mice. Scale bar, 100 μm. n = 6 biologically independent samples.

(**G**) Representative images of EVG staining and quantitative analysis of elastic degradation grade in AAA tissue of WT and R26-GPX4 mice. Scale bar, 500 μm. n = 8 biologically independent samples.

Data expressed the mean±SEM. **$P<0.01$. Comparisons of parameters were performed with One-Way ANOVA followed by a Tukey's multiple comparisons test.

Supplemental Figure 10

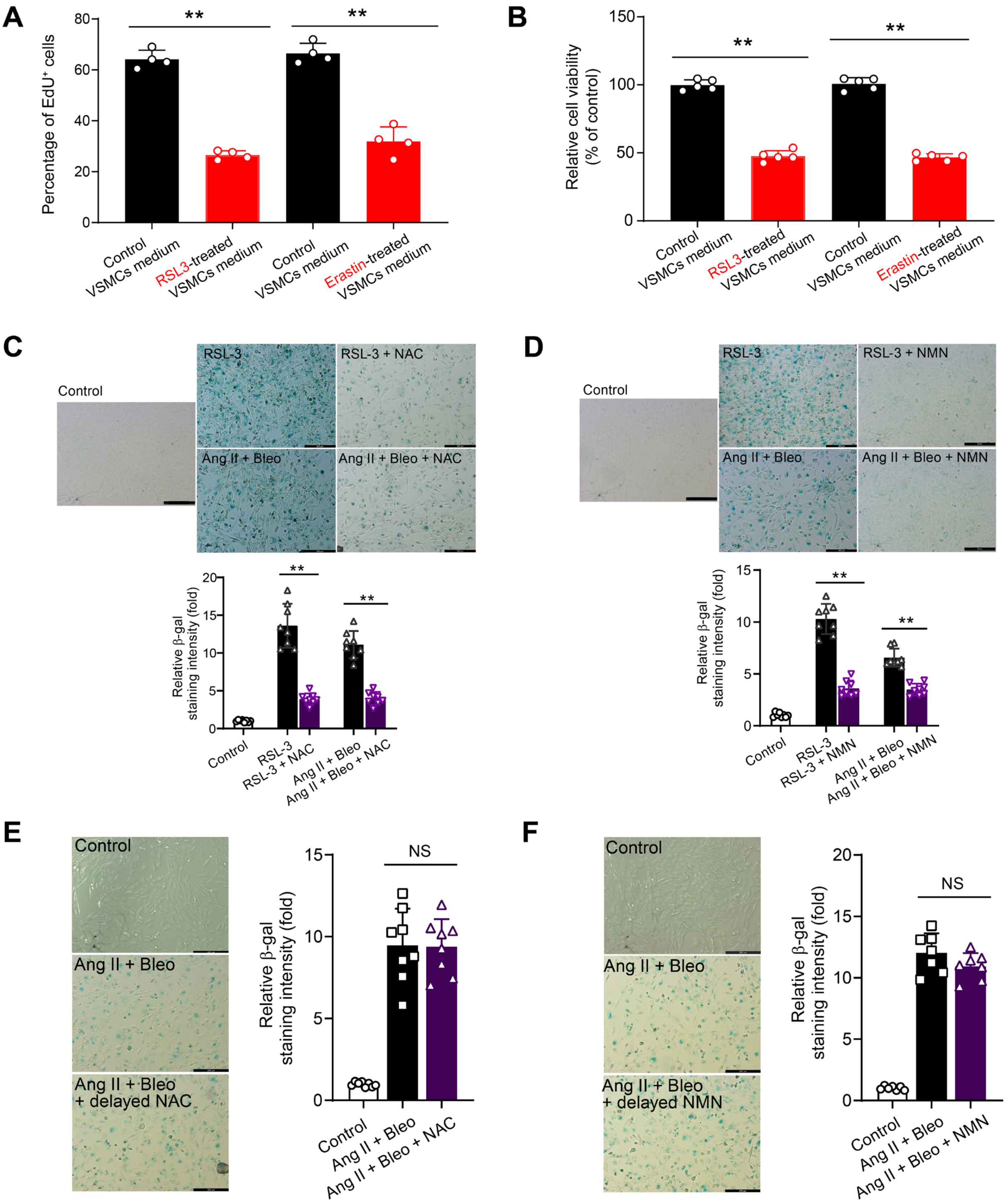

Supplemental Figure 10. Effects of RSL3/erastin-treated VSMCs culture medium, ROS scavenger N-acetyl-L-cysteine (NAC), ferroptosis inhibitor liproxstatin-1 and NAD$^+$ precursor nicotinamide mononucleotide (NMN) on vascular senescence.

(**A-B**) VSMCs received transferred culture medium from RSL3/erastin at cytotoxic dose (10 μM)-treated VSMCs, and their proliferation and viability were measured using EdU incorporation and CCK-8 kit respectively. n = 4-5 biologically independent samples.

(**C-D**) SA-β-gal staining of VSMCs as indicated in the figure. Ferroptosis inducer RSL3 (0.5 μM) and Ang II plus bleomycin (Ang II+Bleo) were added into the culture medium to induce

senescence. ROS scavenger NAC (5 mM) or NAD^+ precursor NMN (300 μM) was simultaneously administrated into the medium. Five days later, the SA-β-gal staining was performed in the VSMCs. n = 8 biologically independent samples.

(**E-F**) SA-β-gal staining of VSMCs as indicated in the figure. Ang II plus bleomycin (Ang II+Bleo) were added into the culture medium to induce senescence for 5 days. Then, ROS scavenger NAC (5 mM, **E**) or NAD^+ precursor NMN (300 μM, **F**) was then administrated into the medium for additional 2 days. The SA-β-gal staining was performed in the VSMCs. n = 7-8 biologically independent samples.

Data expressed the mean±SEM. $^{**}P<0.01$. Comparisons of parameters were performed with Two-sided Unpaired t-test. NS, no significance.

Supplemental Figure 11

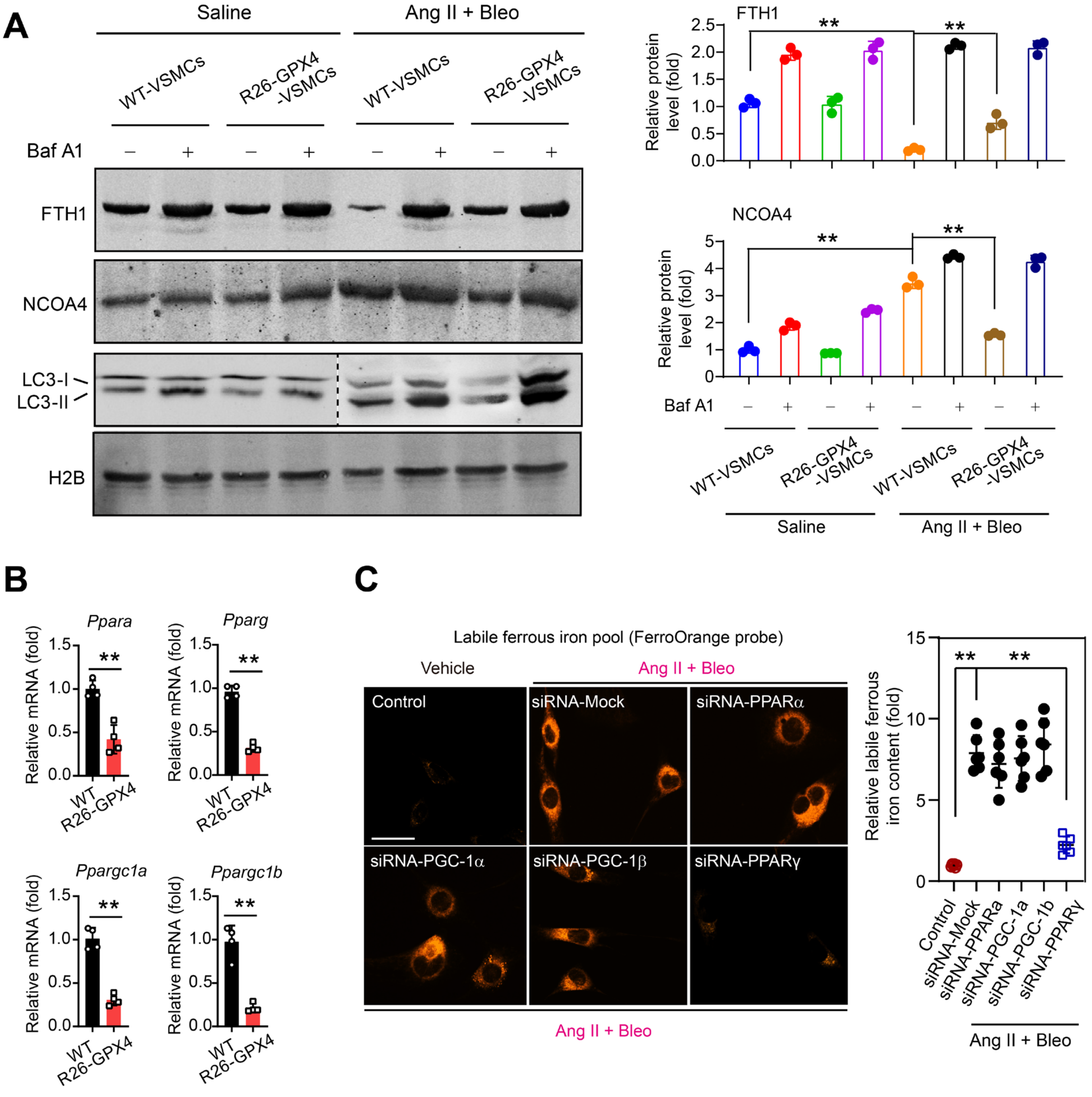

Supplemental Figure 11. PPARγ is involved in ferritinophagy in VSMCs upon Ang II + Bleo stress.
(A) Immunoblotting analyses of autophagic flux in VSMCs isolated from aortae of WT and R26-GPX4 mice under bafilomycin A1. Ang II+Bleo was used to stimulate VSMCs. Histone 2B (H2B) was used to demonstrate that full lysis has been achieved and for a loading control. n = 3 biologically independent samples.
(B) Quantitative PCR analyses were performed to confirm the differentially expressed PPARγ–related genes which had been revealed by RNA-sequencing in aortae from WT mice and GPX4 knock-in mice (R26-GPX4) infused with Ang II+Bleo. The differentially expressed PPARγ–related genes include *Ppara, Pparg, Ppargc1a* and *Ppargc1b*. n = 4 biologically independent samples.
(C) Identification of key controller in ferritinophagy with RNAi-based strategy in a labile iron pool (LIP) assay. VSMCs were transfected with siRNAs targeting PPAR-associated molecules,

including PPARα, PGC-1α, PGC-1β and PPARγ, and incubated with Ang II+Bleo and FerroOrange probe to detect the LIP level. The FerroOrange probe fluorescent intensity is proportional to the LIP. Scale bar, 20 μm. n = 6 biologically independent samples.
Data expressed the mean±SEM. $^{**}P<0.01$, $^{**}P<0.001$. Comparisons of parameters were performed with two-sided unpaired t test.

Supplemental Figure 12

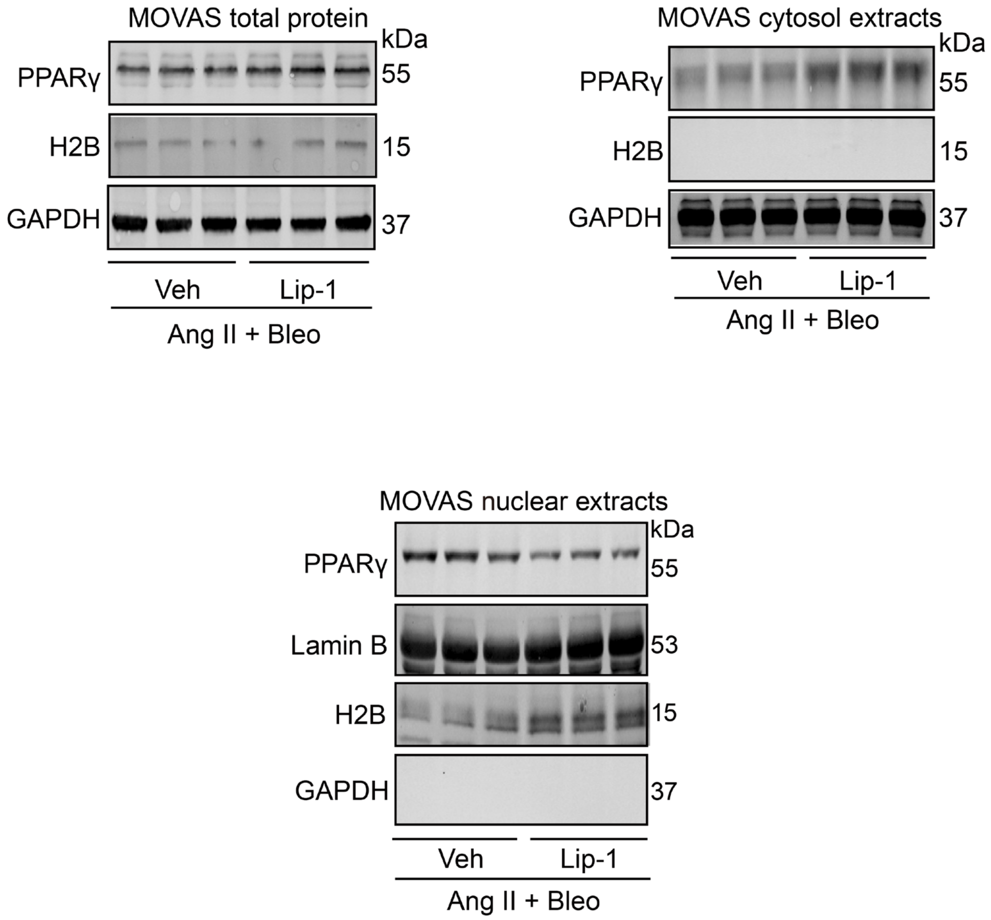

Supplemental Figure 12. Ferroptosis inhibitor liproxstatin-1 regulates PPARγ nuclear-cytoplasm shuttling. PPARγ protein expression in the total, cytosol and nuclear extracts of VSMCs treated with liproxstatin-1 under Ang II+Bleo stress. Histone 2B (H2B) and Lamin B were used as indicators of nuclear extract. GAPDH was used as an indicator of cytosol extract. n = 3 biologically independent samples.

Supplemental Figure 13

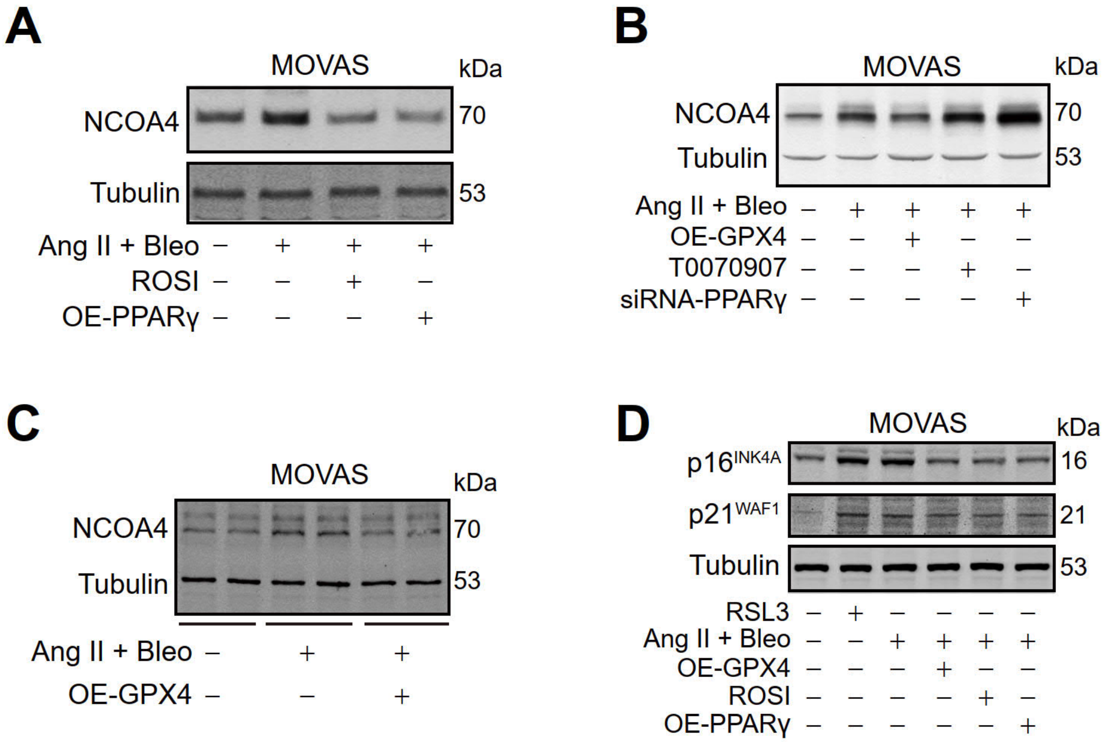

Supplemental Figure 13. Inhibition of ferroptosis signaling regulates NCOA4 expression.

(**A**) Immunoblotting analysis of NCOA4 in cultured VSMCs with Ang II+Bleo stress, overexpression of PPARγ and PPARγ selective agonist rosiglitazone (ROSI, 10 mM).

(**B**) Immunoblotting analysis of NCOA4 in in cultured VSMCs treated with Ang II+Bleo or overexpression of GPX4.

(**C**) Immunoblotting analysis of NCOA4 in cultured VSMCs with Ang II+Bleo stress, overexpression of GPX4, PPARγ selective antagonist T0070907 (10 mM) and knockdown of PPARγ with siRNA.

(**D**) Effects of ferroptosis inducer RSL3, Ang II+Bleo, GPX4 overexpression, PPARγ selective agonist rosiglitazone (ROSI) and overexpression of PPARγ on senescence markers $p16^{INK4A}$ and $p21^{WAF1}$ in cultured VSMCs.

All experiments were repeated for three times.

Supplemental Figure 14

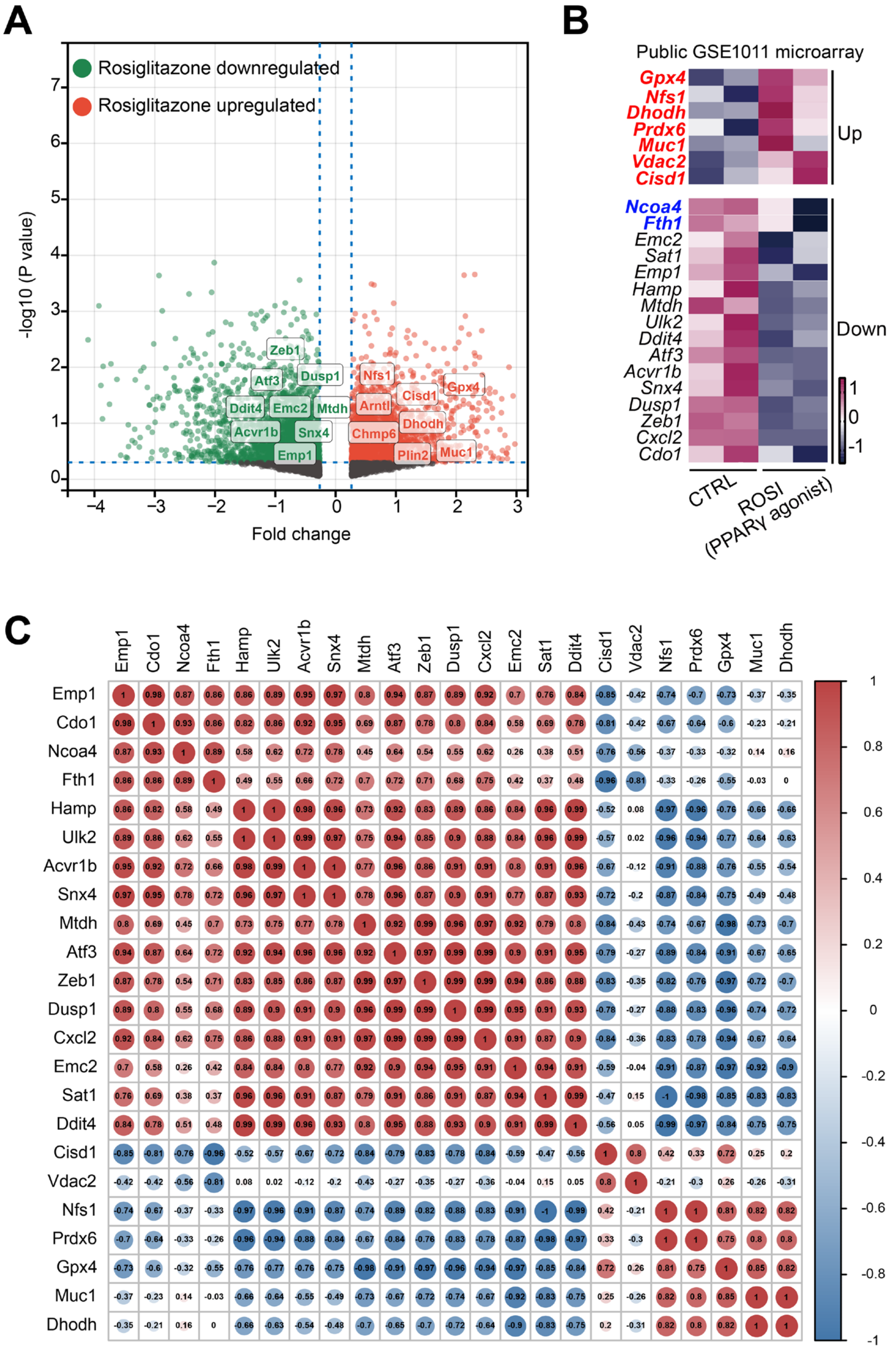

Supplemental Figure 14. Analysis of altered genes in aorta of mice treated with a PPARγ agonist rosiglitazone or vehicle based on a public gene expression data set (GSE1011). **(A)** Volcano plot shows the upregulated and downregulated genes by rosiglitazone treatment for 21 days in mice aorta.

(**B**) Heatmap derived from a public microarray data (No. GSE1011, https://www.ncbi.nlm.nih.gov/geo/query/acc.cgi?acc=GSE1011) showing the upregulated or downregulated genes by rosiglitazone (ROSI) treatment in mice aorta.

(C) Correlation plot shows the correlation between the upregulated and downregulated genes by rosiglitazone treatment in mice aorta.

Supplemental Figure 15

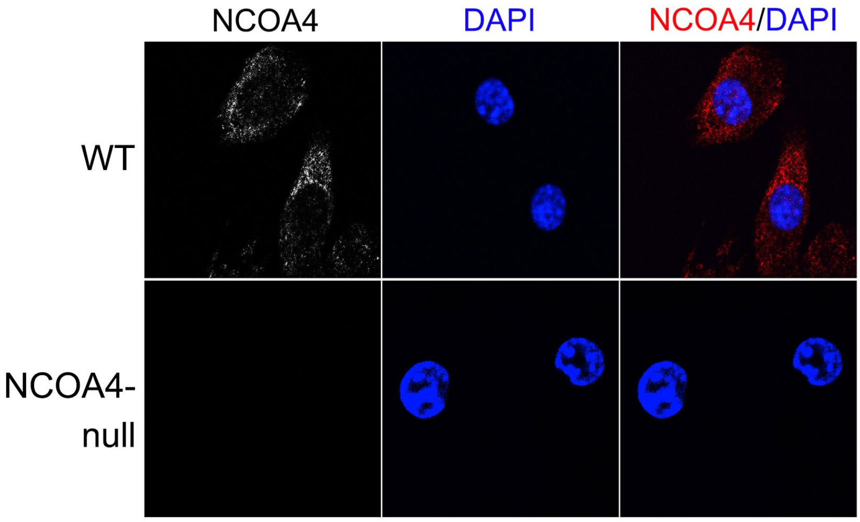

Supplemental Figure 15. Confirmation of anti-NCOA4 in VSMCs with NCOA4 knockout by CRISPR/Cas9. For depleting NCOA4 in cells, the mouse VSMCs was transfected with CRISPR/Cas9 plasmid (Addgene) targeting NCOA4. Two days later, cells were treated with puromycin (2 μg/ml) for 5 days to select positive clones. The survived cells (NCOA4-null cells) were kept for culture and expanded. The cells were stained with anti-NCOA4 (#sc-373739, Santa Cruz Biotechnology) followed by Alexa Fluor 555-conjugated secondary antibody. DAPI was used to stain nuclei. Images were obtained in FluoView™ FV1000 Confocal (Olympus, Tokyo, Japan). Experiments were repeated for three times.

Supplemental Table 1

Gene name	FC(KI/ WT)	Log2FC(KI/WT)	P value (deseq2)	P adjust (deseq2)	Significant	Regulate	WT1	WT2	WT3	R26-GPX4-1	R26-GPX4-2	R26-GPX4-3	WT mean	R26-GPX4 mean
**Alb**	0.05	-4.323467	3E-10	1E-06	yes	down	8.58	2.15	3.53	0.17	0.51	0.17	4.7533	0.283333333
**Cox7a1**	0.091	-3.463093	4E-08	2E-05	yes	down	40.33	165.81	63.82	5.19	21.77	6.24	89.987	11.06666667
**Retnla**	0.121	-3.047518	0.0006	0.0237	yes	down	7.81	3.16	92.26	13.16	0.46	0.71	34.41	4.776666667
**Cox8b**	0.132	-2.91809	6E-10	2E-06	yes	down	209.25	680.19	293.1	43.01	99.35	69.75	394.18	70.70333333
**Car14**	0.14	-2.834804	6E-05	0.004	yes	down	0.47	3.06	1.38	0.57	0.22	0.18	1.6367	0.323333333
**Cyp2b10**	0.157	-2.671312	0.0002	0.0085	yes	down	1.57	11.15	0.93	0.69	1.73	0.34	4.55	0.92
**Ighg2c**	0.159	-2.655311	0.0013	0.04	yes	down	0.43	8.35	0.55	0.41	0.81	0.72	3.11	0.646666667
**Cidea**	0.16	-2.640191	6E-10	2E-06	yes	down	87.84	251.96	145.6	27.1	51.2	20.05	161.81	32.78333333
**Slc27a2**	0.162	-2.625021	2E-06	0.0003	yes	down	2.17	11.72	6.55	0.73	2.55	1.36	6.8133	1.546666667
**Hpd**	0.162	-2.622886	0.0002	0.0115	yes	down	1.59	2.84	1.77	1.03	0.14	0.33	2.0667	0.5
**Hpx**	0.173	-2.528025	7E-06	0.0008	yes	down	3.91	1.34	1.75	0.38	0.55	0.49	2.3333	0.473333333
**Ighm**	0.174	-2.519223	0.0001	0.008	yes	down	7.47	118.6	8.15	10.52	11.32	5.63	44.74	9.156666667
**Cpt1b**	0.184	-2.442181	1E-06	0.0002	yes	down	8.95	29.97	11.76	1.81	7.55	1.99	16.893	3.783333333
**Igkc**	0.2	-2.322934	0.0002	0.0089	yes	down	18.13	282.79	43.92	21.7	19.13	49.32	114.95	30.05
**Inca1**	0.22	-2.187018	0.0001	0.0069	yes	down	1.77	7.4	4.73	1.29	0.33	1.96	4.6333	1.193333333
**Depp1**	0.22	-2.183419	6E-06	0.0007	yes	down	20.02	80.3	22.34	5.09	10.31	18.83	40.887	11.41
**Adra1a**	0.224	-2.157694	1E-06	0.0002	yes	down	1.39	3.23	1.26	0.38	0.67	0.63	1.96	0.56
**Sox6os**	0.229	-2.124251	0.0016	0.0469	yes	down	5.22	11.62	6.63	2.26	1.01	3.64	7.8233	2.303333333
**Aspg**	0.231	-2.115821	6E-06	0.0008	yes	down	3.15	16.65	7.42	1.62	6.21	2.11	9.0733	3.313333333
**Plin5**	0.232	-2.105759	6E-07	0.0001	yes	down	6.13	15.31	8.79	1.22	4.02	3.71	10.077	2.983333333
**Ces1d**	0.245	-2.026354	6E-12	2E-07	yes	down	71.57	125.38	72.84	26.99	29.51	27.81	89.93	28.10333333
**mt-Co3**	0.247	-2.017589	1E-08	8E-06	yes	down	1356.6	1987.24	913.7	701.35	287.2	317.07	1419.2	435.2066667
**Poln**	0.25	-2.001152	0.0002	0.0111	yes	down	3.41	2.78	3.69	0.5	1.21	0.9	3.2933	0.87
**Gys2**	0.253	-1.983895	1E-06	0.0002	yes	down	4.12	2.97	6.29	0.94	2.81	1.77	4.46	1.84
**Tnnt3**	0.257	-1.959299	0.0005	0.0198	yes	down	2.34	5.66	2.36	1.31	0.91	1.19	3.4533	1.136666667
**Coq8a**	0.257	-1.960625	1E-08	1E-05	yes	down	34.26	111.17	43.61	22.49	20.75	18.8	63.013	20.68
**Adig**	0.257	-1.962604	7E-09	6E-06	yes	down	75.41	140.11	103.5	23.37	23.59	55.05	106.35	34.00333333

**Eci3**	0.258	-1.954351	0.0006	0.0213	yes	down	1.93	1.43	1.67	0.28	0.42	1.27	1.6767	0.656666667
**Pck1**	0.264	-1.920763	2E-10	1E-06	yes	down	146.45	265.31	142.2	75.61	53.92	51.42	184.67	60.31666667
**Apoc1**	0.266	-1.909998	5E-06	0.0006	yes	down	181.95	545.4	321.2	50.13	89.06	233.4	349.53	124.1966667
**Mtarc1**	0.269	-1.894726	0.0004	0.0162	yes	down	1.92	0.86	2.25	0.32	0.29	1.23	1.6767	0.613333333
**Fcor**	0.273	-1.871088	7E-05	0.0045	yes	down	40.35	71.21	55.72	9.29	9.12	43.35	55.76	20.58666667
**Ppargc1a**	0.279	-1.83967	5E-07	0.0001	yes	down	2.67	3.32	1.35	0.59	1.28	0.75	2.4467	0.873333333
**Fam13a**	0.283	-1.822769	2E-07	5E-05	yes	down	7.64	24.57	12.95	5.66	3.59	6.71	15.053	5.32
**Retn**	0.284	-1.816173	1E-08	1E-05	yes	down	196.31	210.08	206.6	47.61	41.85	127.58	204.33	72.34666667
**Klb**	0.285	-1.809722	8E-07	0.0002	yes	down	5.75	5.18	2.89	1.5	0.73	2.51	4.6067	1.58
**Calml3**	0.288	-1.798113	0.0014	0.0421	yes	down	1.15	8.45	2.13	0.95	1.18	2.24	3.91	1.456666667
**Acsm5**	0.29	-1.78386	0.0002	0.0113	yes	down	1.09	1.42	1.18	0.38	0.44	0.59	1.23	0.47
**Fgl1**	0.297	-1.752497	3E-05	0.0023	yes	down	22.96	70.83	37.23	6.15	13.78	27.96	43.673	15.96333333
**Lyz1**	0.299	-1.739737	1E-05	0.0012	yes	down	6.22	7.14	19.73	3.56	3.64	5.21	11.03	4.136666667
**Rn7s2**	0.308	-1.697271	6E-08	2E-05	yes	down	451.95	199.61	574	163.89	165.65	164.14	408.5	164.56
**Rn7s1**	0.308	-1.697271	6E-08	2E-05	yes	down	451.95	199.61	574	163.89	165.65	164.14	408.5	164.56
**Acsm3**	0.308	-1.698201	2E-08	1E-05	yes	down	16.62	33.84	26.66	8.59	11.22	12.86	25.707	10.89
**Acaa1b**	0.309	-1.692963	2E-06	0.0003	yes	down	7.98	10.26	8.62	2.24	5.38	2.95	8.9533	3.523333333
**Immp2l**	0.31	-1.68956	0.0001	0.007	yes	down	7.03	25.9	8.65	9.64	11.58	11.44	13.86	10.88666667
**Ctcflos**	0.312	-1.681918	0.0005	0.0188	yes	down	1	5.97	1.76	1.51	0.7	1.12	2.91	1.11
**Acot4**	0.312	-1.680532	5E-05	0.0034	yes	down	1.04	0.97	1.59	0.74	0.39	0.32	1.2	0.483333333
**Fasn**	0.315	-1.665267	2E-06	0.0004	yes	down	607.11	210.85	212.7	103.11	145.47	197.82	343.54	148.8
**Myo5c**	0.319	-1.646622	0.0009	0.0321	yes	down	1.61	1.27	0.41	0.11	0.31	0.25	1.0967	0.223333333
**Prtn3**	0.32	-1.645006	0.0007	0.0258	yes	down	5.01	19.43	11.81	2.31	2.82	9.18	12.083	4.77
**Lncbate1**	0.32	-1.645632	0.0012	0.0398	yes	down	4.17	6.27	8.48	1.28	1.12	5.38	6.3067	2.593333333
**Rbp7**	0.322	-1.636624	0.0001	0.0072	yes	down	10.75	24.72	14.92	4.98	4.14	11.76	16.797	6.96
**Otop1**	0.322	-1.636726	2E-05	0.002	yes	down	3.65	3.19	1.82	0.69	1.88	0.8	2.8867	1.123333333
**Orm1**	0.325	-1.622189	8E-07	0.0002	yes	down	71.05	58.98	59.07	11.89	19.33	45.6	63.033	25.60666667
**Lgals4**	0.328	-1.607202	0.0002	0.0087	yes	down	3.96	7.8	3.46	2.22	0.84	2.36	5.0733	1.806666667
**Gpt**	0.33	-1.600179	2E-09	4E-06	yes	down	14.5	15.28	13.6	4.78	6.15	7.89	14.46	6.273333333
**Chchd10**	0.33	-1.599791	5E-07	0.0001	yes	down	103.89	306.58	176	56.9	69.81	106	195.5	77.57
**Ifi27l2a**	0.334	-1.582667	7E-07	0.0002	yes	down	1640.6	2676.24	3007	732.67	635.63	1802.82	2441.4	1057.04

**H2-Q10**	0.336	-1.571487	5E-08	2E-05	yes	down	12.74	11.67	13.44	4.06	4.07	7.14	12.617	5.09
**Gsta3**	0.337	-1.570343	8E-07	0.0002	yes	down	19.4	13.66	7.73	6.48	6.05	6.15	13.597	6.226666667
**Mycl**	0.338	-1.563499	0.0003	0.0129	yes	down	1.58	4.75	1.93	1.14	0.5	1.83	2.7533	1.156666667
**Acaa2**	0.338	-1.56544	5E-09	6E-06	yes	down	82.05	157.07	87.19	40.63	55.11	41.65	108.77	45.79666667
**Dgat2**	0.341	-1.553221	4E-10	1E-06	yes	down	74.59	104.12	74.41	24.85	39.38	42.38	84.373	35.53666667
**Tmem79**	0.344	-1.537746	0.0015	0.0449	yes	down	2.64	2.64	0.8	0.63	1.25	1.2	2.0267	1.026666667
**Acss3**	0.344	-1.538524	2E-06	0.0004	yes	down	4.97	11.05	4.99	2.43	2.02	4.43	7.0033	2.96
**Slc22a1**	0.347	-1.525816	0.0005	0.0185	yes	down	22.07	16.12	19.77	13.88	6.29	3.57	19.32	7.913333333
**Adtrp**	0.349	-1.518689	3E-06	0.0005	yes	down	15.05	36.97	26.29	6.74	7.07	15.73	26.103	9.846666667
**Fabp4**	0.352	-1.505192	1E-05	0.0011	yes	down	2562.1	4526.58	3529	892.61	975.95	2821.18	3539.3	1563.246667
**Cfd**	0.354	-1.499381	3E-05	0.0024	yes	down	3706.6	7638.11	6058	1655.26	1393.49	4744.22	5800.9	2597.656667
**Inmt**	0.355	-1.495678	0.0009	0.0304	yes	down	36.6	8.3	33.23	22.49	5.28	4.68	26.043	10.81666667
**Arxes1**	0.355	-1.492174	2E-06	0.0003	yes	down	10.75	7.52	9.5	3.17	2.84	6.07	9.2567	4.026666667
**Thrsp**	0.356	-1.490782	3E-09	4E-06	yes	down	337.51	249.09	223.4	72.78	113.1	165.07	269.99	116.9833333
**Ppp1r1a**	0.359	-1.479438	3E-08	2E-05	yes	down	17.12	18.28	20.12	6.67	7.4	10.34	18.507	8.136666667
**Ppara**	0.359	-1.476132	0.0004	0.016	yes	down	1.71	3.53	1.77	0.93	2.11	0.43	2.3367	1.156666667
**Amy1**	0.359	-1.478266	5E-08	2E-05	yes	down	17.42	26.59	19.42	8.15	7.4	14.27	21.143	9.94
**Kcnk3**	0.36	-1.471983	2E-06	0.0003	yes	down	9.49	21.89	12.73	4.32	7.05	9.43	14.703	6.933333333
**Scd1**	0.362	-1.465021	2E-06	0.0003	yes	down	884.5	587.25	338.3	138.28	330.5	314.33	603.36	261.0366667
**Prkar2b**	0.369	-1.43908	3E-09	4E-06	yes	down	38.17	47.27	38.4	13.91	16.42	26.57	41.28	18.96666667
**Ankef1**	0.369	-1.436773	4E-05	0.0031	yes	down	9.27	11.6	7.04	3.61	1.75	6.44	9.3033	3.933333333
**Rgcc**	0.37	-1.433518	4E-07	1E-04	yes	down	73.98	148.73	104.4	42.7	35.05	75.14	109.03	50.96333333
**Ppargc1l**	0.371	-1.42986	0.001	0.0328	yes	down	0.84	2.1	0.8	0.53	0.77	0.42	1.2467	0.573333333
**Tuba8**	0.372	-1.428135	4E-05	0.0028	yes	down	2.31	3.22	3.13	1.22	1.24	1.52	2.8867	1.326666667
**Rn7sk**	0.374	-1.4197	1E-05	0.0011	yes	down	95.52	75.04	146.7	50.84	49.36	54.5	105.77	51.56666667
**Nat8l**	0.378	-1.40263	2E-07	6E-05	yes	down	3.31	3.18	2.01	1.25	1.59	1.06	2.8333	1.3
**Slc36a2**	0.385	-1.378193	2E-06	0.0003	yes	down	35.5	68.02	44.04	17.56	15.72	37.78	49.187	23.68666667
**Fmc1**	0.385	-1.376937	1E-06	0.0002	yes	down	49.26	62.7	66.45	33.51	29.2	24	59.47	28.90333333
**B3galt2**	0.385	-1.375624	2E-05	0.0021	yes	down	4.42	4.49	3.86	2.34	0.61	3.05	4.2567	2
**Ces1f**	0.39	-1.358209	1E-06	0.0002	yes	down	10.1	17.47	17.05	5.73	6.56	9.1	14.873	7.13
**Lipe**	0.391	-1.35445	4E-08	2E-05	yes	down	101.56	145.51	116.3	55.51	38.95	85.96	121.13	60.14

**Acss1**	0.392	-1.35064	9E-06	0.001	yes	down	4.16	11.11	5.54	3.49	3.31	3.4	6.9367	3.4
**Rpl37rt**	0.394	-1.342428	5E-06	0.0006	yes	down	251.48	239.69	195.7	115.29	168.75	64.98	228.96	116.34
**Pcx**	0.394	-1.342567	6E-09	6E-06	yes	down	76.28	71.6	56.9	25.11	26.57	47.35	68.26	33.01
**Adhfe1**	0.395	-1.340521	5E-06	0.0006	yes	down	15.63	30.97	26.42	8.56	9.89	19.22	24.34	12.55666667
**Pfkfb1**	0.397	-1.33147	2E-05	0.0016	yes	down	8.42	9.13	9.36	3.78	2.56	6.63	8.97	4.323333333
**Nrg4**	0.397	-1.332072	5E-07	0.0001	yes	down	12.92	16.74	18.88	5	8.43	9.58	16.18	7.67
**Slc2a4**	0.398	-1.329187	1E-07	3E-05	yes	down	21.55	26.19	30.13	10.74	9.94	18.13	25.957	12.93666667
**Clstn3**	0.402	-1.315903	8E-07	0.0002	yes	down	32.39	37.59	31.39	9.38	15.14	22.02	33.79	15.51333333
**Dbi**	0.403	-1.311435	2E-09	4E-06	yes	down	1084.4	1256.39	1090	501.97	442.12	789.89	1143.5	577.9933333
**Cyp2e1**	0.403	-1.312921	3E-05	0.0026	yes	down	278.94	723.19	580.1	218.99	166.26	418.31	527.42	267.8533333
**Etfb**	0.404	-1.307243	3E-09	4E-06	yes	down	285.36	446.63	313.2	161.61	177.54	201.31	348.4	180.1533333
**Cyp2f2**	0.404	-1.30819	7E-05	0.0045	yes	down	18.72	56.88	36.66	11.36	16.36	29.8	37.42	19.17333333
**Pank1**	0.405	-1.302896	4E-06	0.0006	yes	down	4.39	9.4	6.3	2.29	4.36	2.93	6.6967	3.193333333
**Lpl**	0.408	-1.293639	2E-07	5E-05	yes	down	199.42	336.78	255.7	117.14	108.02	173.8	263.97	132.9866667
**Acacb**	0.408	-1.292436	3E-08	2E-05	yes	down	17.4	26.03	13.35	7.91	10.36	10.45	18.927	9.573333333
**Dgat1**	0.411	-1.281954	7E-10	2E-06	yes	down	62.2	80.84	70.45	30.3	31.16	42.51	71.163	34.65666667
**Aldh1a7**	0.413	-1.274091	3E-06	0.0005	yes	down	10.6	6.52	5.55	3.27	3.38	4.64	7.5567	3.763333333
**Rassf6**	0.415	-1.268357	0.0005	0.0194	yes	down	2.92	3.94	5	3.31	1.4	1.26	3.9533	1.99
**Atp5md**	0.415	-1.268527	2E-11	3E-07	yes	down	671.84	746.16	702.1	366.96	349.54	419.99	706.69	378.83
**Agpat2**	0.42	-1.251485	3E-07	7E-05	yes	down	195.02	85.98	117.9	42.46	77.91	80.92	132.98	67.09666667
**Gpd1**	0.421	-1.247073	2E-07	6E-05	yes	down	186.77	215.32	176.3	63.75	92.27	144.71	192.78	100.2433333
**Elovl6**	0.422	-1.243882	2E-06	0.0004	yes	down	11.88	7.15	4.78	2.76	5.54	3.91	7.9367	4.07
**Eci1**	0.423	-1.239747	4E-09	5E-06	yes	down	83.05	122.23	88.2	46.04	49.51	55.95	97.827	50.5
**Tcap**	0.425	-1.232952	0.0014	0.0428	yes	down	44.58	17.79	82.28	42.42	10.46	21.74	48.217	24.87333333
**Shmt1**	0.425	-1.232785	0.0015	0.0449	yes	down	1.62	2.55	5.19	1.55	1.19	1.19	3.12	1.31
**Atp1a2**	0.426	-1.23204	4E-10	2E-06	yes	down	70.22	78.2	54.03	34.17	31.87	36.29	67.483	34.11
**Oxld1**	0.43	-1.215946	0.0002	0.0092	yes	down	11.44	13.62	10.58	8.23	4.87	5.37	11.88	6.156666667
**Ech1**	0.43	-1.215966	4E-09	5E-06	yes	down	150.17	227.44	173.6	92.02	95.9	106.79	183.74	98.23666667
**Acss2**	0.431	-1.213117	9E-06	0.001	yes	down	37.47	12.24	13.55	8.79	12.75	12.18	21.087	11.24
**Hcar2**	0.434	-1.204922	0.0002	0.0115	yes	down	4.1	7.65	6.19	3.92	1.72	4.01	5.98	3.216666667
**Chdh**	0.434	-1.203242	3E-06	0.0005	yes	down	3.35	4.64	3.66	1.94	1.44	2.86	3.8833	2.08

**Cox7b**	0.436	-1.198979	1E-10	1E-06	yes	down	174.32	274.26	173	104.71	134.43	105.1	207.19	114.7466667
**Rgs2**	0.437	-1.195917	2E-07	6E-05	yes	down	36.9	44.47	26.05	18.84	13.95	24.63	35.807	19.14
**Mcrip2**	0.438	-1.19219	3E-07	8E-05	yes	down	37.07	50.09	42.98	19.43	20.55	33.5	43.38	24.49333333
**Clec2d**	0.438	-1.192516	6E-08	2E-05	yes	down	64.2	83.36	73.8	42.31	26.57	51.14	73.787	40.00666667
**Pxmp2**	0.439	-1.186737	0.0002	0.0102	yes	down	11.26	17.57	16.3	6.57	4.69	12.36	15.043	7.873333333
**Acadm**	0.442	-1.176529	1E-08	8E-06	yes	down	147.45	194.11	131.6	75.12	86.53	93.38	157.73	85.01
**Tshr**	0.444	-1.171434	0.0008	0.0271	yes	down	4.36	4.02	2	1.6	0.9	2.88	3.46	1.793333333
**Mrap**	0.444	-1.171153	9E-05	0.0057	yes	down	76.12	101.25	76.32	26.5	28.89	87.76	84.563	47.71666667
**Cmbl**	0.445	-1.167261	9E-05	0.0056	yes	down	18.55	27.36	20.93	9.66	7.89	20.79	22.28	12.78
**Aldh3b2**	0.448	-1.15844	0.0003	0.0118	yes	down	5.02	6.05	5.73	1.83	2.44	5.09	5.6	3.12
**Vat1l**	0.449	-1.154575	0.0004	0.0173	yes	down	5.31	13.08	12.88	4.07	2.65	10.06	10.423	5.593333333
**Pygl**	0.45	-1.151188	2E-08	1E-05	yes	down	23.9	26.77	15.7	11.51	14.64	12.45	22.123	12.86666667
**Pet100**	0.45	-1.15091	3E-06	0.0005	yes	down	31.81	44.66	44.57	18.38	16.84	23.05	40.347	19.42333333
**Hadhb**	0.451	-1.150131	4E-08	2E-05	yes	down	145.45	252.9	166.9	95.8	122.34	119.39	188.42	112.51
**Aspa**	0.451	-1.14894	0.0002	0.0096	yes	down	8.1	9.38	10.52	3.15	5.09	6	9.3333	4.746666667
**Aqp7**	0.451	-1.148831	0.0003	0.0143	yes	down	6.3	16.97	8.55	4.28	3.89	9.31	10.607	5.826666667
**Acvr1c**	0.451	-1.148895	0.0001	0.0076	yes	down	3.12	3.65	2.42	1.42	0.88	2.53	3.0633	1.61
**Nudt7**	0.452	-1.145242	7E-08	3E-05	yes	down	22.92	27.15	24.62	11.62	10.28	16.95	24.897	12.95
**Ndufb4c**	0.455	-1.136208	7E-08	2E-05	yes	down	255.24	270.33	240.8	139.56	117.87	192.74	255.45	150.0566667
**Hibch**	0.456	-1.133234	3E-08	2E-05	yes	down	29.02	43.46	39.28	19.52	21.38	22.37	37.253	21.09
**Aco2**	0.456	-1.133441	2E-07	6E-05	yes	down	166.57	255.34	154.2	101.07	119.83	98.18	192.04	106.36
**mt-Co2**	0.458	-1.127222	2E-05	0.0018	yes	down	732.54	1409.69	579.2	649.47	385.08	535.18	907.14	523.2433333
**Acadvl**	0.458	-1.126442	1E-07	5E-05	yes	down	114.08	190.21	123.6	74.01	90.77	79.82	142.64	81.53333333
**Tmem45l**	0.459	-1.124145	7E-06	0.0008	yes	down	58.89	51.67	51.62	27.03	14.06	49.94	54.06	30.34333333
**Cidec**	0.462	-1.114803	7E-05	0.0045	yes	down	213.99	389.67	361.5	149.34	112.85	270.83	321.72	177.6733333
**Cox7a2**	0.465	-1.103627	5E-10	2E-06	yes	down	240.41	286.36	257.9	158.89	140.7	155.44	261.57	151.6766667
**Atp5k**	0.467	-1.098651	7E-09	6E-06	yes	down	392.99	460.11	442.2	285.28	235.72	258.63	431.78	259.8766667
**Tcim**	0.468	-1.0962	0.0001	0.0067	yes	down	17.3	36.93	24.79	11.07	10.95	24.48	26.34	15.5
**Plin1**	0.468	-1.096747	3E-05	0.0023	yes	down	137.43	139.34	130	55.3	43.05	127.68	135.59	75.34333333
**Kyat3**	0.47	-1.087772	3E-05	0.0027	yes	down	9.43	13.23	9.11	4.13	5.51	10.3	10.59	6.646666667
**Ffar4**	0.47	-1.089276	0.0008	0.027	yes	down	4.34	4.81	6.28	3.56	2.06	3.29	5.1433	2.97

**Adipoq**	0.471	-1.086105	0.001	0.0333	yes	down	309.51	623.13	482.2	183.68	130.62	518.34	471.6	277.5466667
**Rnf125**	0.473	-1.081561	0.0001	0.0081	yes	down	12	20.45	6.14	7.22	6.22	9.9	12.863	7.78
**Aldh6a1**	0.473	-1.080778	3E-07	8E-05	yes	down	52.52	76.56	62.37	35.55	31.06	49.03	63.817	38.54666667
**Tmem17!**	0.474	-1.077853	7E-05	0.0048	yes	down	4.94	5.5	5.19	2.32	2.13	4.43	5.21	2.96
**Pparg**	0.474	-1.077836	5E-05	0.0037	yes	down	26.71	57.01	35.85	15.89	13.33	33.3	39.857	20.84
**Cd1d1**	0.475	-1.074056	0.0001	0.0072	yes	down	18.57	28	22	9.8	7.78	20.84	22.857	12.80666667
**Pnpla3**	0.476	-1.070531	0.0009	0.0303	yes	down	20.94	12.65	8.95	3.55	5.94	10.98	14.18	6.823333333
**Proca1**	0.478	-1.063495	0.0009	0.0303	yes	down	7.94	10.42	9.36	3.95	3.64	9.16	9.24	5.583333333
**Lpin1**	0.478	-1.064588	3E-08	2E-05	yes	down	18.25	23.6	16.37	10.71	10.79	13.28	19.407	11.59333333
**Gstz1**	0.479	-1.061647	1E-06	0.0002	yes	down	120.67	150.25	141.8	59.1	56.18	106.11	137.58	73.79666667
**Gpat3**	0.48	-1.058002	2E-05	0.0016	yes	down	6.84	6.01	7.87	3.24	3.21	5.89	6.9067	4.113333333
**Cox6c**	0.481	-1.057185	1E-09	3E-06	yes	down	797.38	949.47	835.3	517.98	505.45	542.69	860.72	522.04
**Adora1**	0.481	-1.05667	3E-06	0.0004	yes	down	6.79	9.84	7.31	4.27	3.75	5.93	7.98	4.65
**Lncppara**	0.483	-1.049515	0.0003	0.0119	yes	down	6.28	3.99	5.65	3.67	1.8	5.03	5.3067	3.5
**Car3**	0.485	-1.044626	0.0005	0.0184	yes	down	1417.9	1532.13	1728	659.84	460.62	1717.29	1559.2	945.9166667
**Ndufa2**	0.486	-1.039519	2E-09	4E-06	yes	down	255.37	306.91	306.4	176.89	167.78	186.38	289.56	177.0166667
**Hadh**	0.486	-1.040193	4E-09	5E-06	yes	down	89.03	111.84	87	55.97	57.15	59.33	95.957	57.48333333
**Echdc3**	0.486	-1.040066	5E-05	0.0039	yes	down	7.01	8.55	6.68	3.6	4.46	4.79	7.4133	4.283333333
**Pnpla2**	0.487	-1.038994	1E-05	0.0012	yes	down	253.65	482.32	418.7	185.19	165.72	306.69	384.89	219.2
**Acadl**	0.487	-1.038033	2E-07	6E-05	yes	down	117.62	184.79	130.7	76.62	89.49	95.34	144.37	87.15
**Hint2**	0.488	-1.034388	9E-07	0.0002	yes	down	67.01	85.46	69.99	40.47	44.6	52.38	74.153	45.81666667
**Ndufb2**	0.489	-1.032486	6E-09	6E-06	yes	down	176.74	185.45	183.8	111.77	94.3	103.68	181.99	103.25
**Echs1**	0.49	-1.028779	6E-08	2E-05	yes	down	83.26	120.05	97.98	57.5	54.66	69.99	100.43	60.71666667
**Selenbp1**	0.492	-1.024369	4E-07	9E-05	yes	down	26.07	36.5	33.65	22.32	15.68	20.34	32.073	19.44666667
**Plaat3**	0.493	-1.018885	1E-06	0.0002	yes	down	42.53	57.48	51.27	28.4	23.18	43.96	50.427	31.84666667
**Nudt8**	0.493	-1.018918	3E-05	0.0027	yes	down	19.54	23.21	23.14	14.8	13.49	12.04	21.963	13.44333333
**Ndufb11**	0.494	-1.018266	2E-09	4E-06	yes	down	204.47	244.12	226.1	138.03	131.83	141.88	224.9	137.2466667
**Arl4a**	0.495	-1.013792	4E-09	5E-06	yes	down	57.82	57.67	55.6	37.57	29.89	39.03	57.03	35.49666667
**Acaca**	0.495	-1.015049	9E-06	0.001	yes	down	50.87	26.47	27.02	16.33	16.88	28.31	34.787	20.50666667
**Mccc2**	0.496	-1.010561	1E-07	4E-05	yes	down	22.84	27.03	21.16	14.32	12.44	17.87	23.677	14.87666667
**Prxl2a**	0.497	-1.008885	8E-07	0.0002	yes	down	40.95	61.66	51.77	32.68	25.99	41.54	51.46	33.40333333

**Acsl1**	0.499	-1.002867	6E-08	2E-05	yes	down	179.77	153.65	136.3	79.6	87.25	119.89	156.57	95.58
**Uqcrq**	0.5	-1.001439	4E-09	5E-06	yes	down	469.95	547.71	473.2	262.65	301.02	329.66	496.94	297.7766667
**Egln3**	0.5	-1.000867	6E-05	0.0042	yes	down	4.38	4.07	4.83	2.99	2.44	2.65	4.4267	2.693333333
**Ccdc69**	0.5	-0.999684	0.0006	0.021	yes	down	6.6	11.1	9.01	6.29	3.59	6.78	8.9033	5.553333333
**Lat2**	2.004	1.002998	0.0002	0.0115	yes	up	4.13	6.79	13.94	21.93	21.97	13.28	8.2867	19.06
**Fgr**	2.005	1.003345	0.001	0.0343	yes	up	1.53	3.56	3.32	10.48	6.24	4.42	2.8033	7.046666667
**Dpep2**	2.013	1.009441	9E-06	0.001	yes	up	5.73	7.77	7.16	19.24	17.75	14.54	6.8867	17.17666667
**Cxcr4**	2.016	1.011548	0.0003	0.0129	yes	up	3.8	7.43	5.44	16.46	15.74	11.1	5.5567	14.43333333
**Slc37a2**	2.027	1.019512	0.0001	0.0063	yes	up	1.34	2.67	3.15	9.12	4.54	5.53	2.3867	6.396666667
**Nxpe5**	2.033	1.023286	0.0002	0.0082	yes	up	1.39	2.39	2.73	6.58	4.81	4.82	2.17	5.403333333
**Plxnc1**	2.04	1.02853	0.0002	0.0114	yes	up	0.8	1.17	2.06	1.84	3.47	1.67	1.3433	2.326666667
**Plek**	2.054	1.038106	4E-06	0.0006	yes	up	5.76	8.91	12.32	27.34	20.97	20.05	8.9967	22.78666667
**Il10ra**	2.067	1.047359	0.0002	0.0088	yes	up	2.52	5.86	5.68	16.56	11.33	8.33	4.6867	12.07333333
**Dock2**	2.1	1.070269	8E-06	0.0009	yes	up	1.57	2.74	3.06	7.61	6.14	5.38	2.4567	6.376666667
**Vav1**	2.107	1.075416	0.0005	0.0184	yes	up	1.69	5.17	7.29	15.14	10.06	9.51	4.7167	11.57
**Slc15a3**	2.107	1.075038	0.0001	0.0064	yes	up	2.92	6.01	8.46	18.66	14.98	10.9	5.7967	14.84666667
**Cx3cr1**	2.11	1.077154	0.0002	0.0089	yes	up	1.8	3.4	4.71	11.99	8.09	6.05	3.3033	8.71
**Stac2**	2.113	1.079198	0.0004	0.0175	yes	up	1.41	1.09	1.1	1.98	4.21	3.59	1.2	3.26
**Pilra**	2.124	1.087006	0.0001	0.0067	yes	up	3.43	5.59	6.04	14.1	10.42	6.99	5.02	10.50333333
**Ch25h**	2.125	1.087441	0.0001	0.0074	yes	up	3.38	5.56	8.19	13.39	16.22	15.98	5.71	15.19666667
**Gpx4**	2.13	1.09074	6E-09	6E-06	yes	up	514.58	601.89	545.4	1545.36	1551.72	1440.44	553.97	1512.506667
**Gpr88**	2.133	1.09307	0.0006	0.022	yes	up	0.77	0.69	1.13	1.99	2.13	2.69	0.8633	2.27
**Lair1**	2.162	1.112617	0.0004	0.0152	yes	up	1.87	4.83	7.41	19.63	9.08	9.01	4.7033	12.57333333
**Cdk18**	2.166	1.114948	0.0001	0.0074	yes	up	0.96	1.14	1.53	5.79	4.72	4	1.21	4.836666667
**Rab11fip**	2.176	1.121665	0.0003	0.0125	yes	up	0.53	1.13	0.77	2.05	2.34	1.21	0.81	1.866666667
**Slamf7**	2.191	1.131784	0.0017	0.0497	yes	up	0.83	2.68	1.38	5.11	3.8	2.88	1.63	3.93
**Fyb**	2.238	1.16213	5E-08	2E-05	yes	up	4.14	6.66	5.51	15.3	11.03	12.72	5.4367	13.01666667
**Neurl3**	2.258	1.174952	2E-05	0.0016	yes	up	3.27	7.26	7.68	21.51	14.82	15.37	6.07	17.23333333
**Cd180**	2.258	1.174745	0.0007	0.025	yes	up	1.1	2.8	2.93	9.66	5.94	3.86	2.2767	6.486666667
**Pou2f2**	2.278	1.187769	0.0017	0.0496	yes	up	0.22	0.6	0.66	2.1	1.33	1.1	0.4933	1.51
**Lcp1**	2.291	1.195892	5E-07	0.0001	yes	up	21.87	33.23	43.16	115.78	89.47	73.98	32.753	93.07666667

**Padi2**	2.299	1.201224	0.0006	0.0212	yes	up	0.45	0.32	0.7	1.35	4.96	1.61	0.49	2.64
**Rrm2**	2.308	1.206614	0.0006	0.022	yes	up	0.89	1.29	2.02	5.12	4.14	2.87	1.4	4.043333333
**Clec12a**	2.311	1.20851	0.0016	0.0467	yes	up	1.1	5.1	6.16	18.28	10.1	8.78	4.12	12.38666667
**Cd72**	2.314	1.210535	2E-05	0.0019	yes	up	10.06	16.31	39.75	93.17	90.65	51.31	22.04	78.37666667
**Stap1**	2.315	1.210791	0.0005	0.0203	yes	up	0.55	1.23	1.74	3.05	2.67	1.98	1.1733	2.566666667
**C1ql3**	2.321	1.214938	0.0013	0.042	yes	up	0.89	0.44	0.79	1.64	2.42	1.9	0.7067	1.986666667
**Il1rn**	2.337	1.224838	0.0012	0.0394	yes	up	1.58	4.26	6.48	13.55	12.86	8.52	4.1067	11.64333333
**Psd4**	2.404	1.265611	8E-06	0.0009	yes	up	0.81	1.34	2.25	6.85	4.94	4.5	1.4667	5.43
**Il21r**	2.425	1.27782	9E-05	0.0058	yes	up	0.97	2.17	2.27	6.48	4.94	4.87	1.8033	5.43
**Slfn10-ps**	2.426	1.278778	0.0013	0.0411	yes	up	0.28	0.41	0.66	1.18	1.62	1.19	0.45	1.33
**Mrip-ps**	2.438	1.285839	0.0011	0.0362	yes	up	2.03	2.63	4.17	9.92	7.88	5.62	2.9433	7.806666667
**Ccr5**	2.438	1.285563	0.0002	0.0089	yes	up	2.36	7.3	10.72	30.09	15.62	16.16	6.7933	20.62333333
**Cemip**	2.439	1.286084	1E-10	1E-06	yes	up	4.1	4.54	3.28	13.35	11.45	10.81	3.9733	11.87
**Parvg**	2.451	1.293639	0.0007	0.025	yes	up	0.54	1.85	2.42	6.94	4.6	3.8	1.6033	5.113333333
**Blnk**	2.504	1.324136	0.0001	0.0069	yes	up	0.99	2.21	3.31	7.42	7.79	5.43	2.17	6.88
**Mmp12**	2.521	1.333952	3E-08	2E-05	yes	up	11.7	13.71	18.77	43.05	59.8	37.94	14.727	46.93
**Trem1**	2.529	1.338697	0.0001	0.0065	yes	up	0.63	1.06	1.09	3.16	2.61	2.57	0.9267	2.78
**Srd5a2**	2.558	1.354945	5E-05	0.0035	yes	up	2.03	2.43	2.68	4.58	10.24	7.84	2.38	7.553333333
**Adam8**	2.647	1.404442	5E-06	0.0007	yes	up	2.88	6.25	8.79	23.06	16.85	18.51	5.9733	19.47333333
**Basp1**	2.666	1.414814	3E-07	7E-05	yes	up	4.61	4.54	7.74	21.69	16.57	15.58	5.63	17.94666667
**Spn**	2.746	1.457301	9E-07	0.0002	yes	up	2.25	2.15	2.18	9.76	8.43	3.74	2.1933	7.31
**Nlrp3**	2.768	1.46907	0.0002	0.0098	yes	up	0.34	0.42	1.19	2.48	1.27	1.75	0.65	1.833333333
**Gjb2**	2.966	1.568667	0.0008	0.0268	yes	up	0.59	0.21	0.72	1.93	2.42	1.1	0.5067	1.816666667
**Clca3a2**	3.38	1.757064	4E-07	9E-05	yes	up	0.59	1.12	1.51	4.43	3.36	5.8	1.0733	4.53
**Map3k9**	3.648	1.867011	0.0003	0.0136	yes	up	0.12	0.12	0.15	0.65	0.51	0.57	0.13	0.576666667
**Il7r**	4.421	2.144425	6E-05	0.0042	yes	up	0.08	0.44	0.3	2.55	0.76	1.83	0.2733	1.713333333
**Actc1**	4.485	2.16521	0.0005	0.0204	yes	up	1.1	1.46	1.45	2.56	1.25	18.98	1.3367	7.596666667
**Gpr31c**	4.611	2.205088	5E-05	0.0035	yes	up	0.2	0.08	0.29	1.72	0.91	0.52	0.19	1.05
**Myh6**	9.172	3.197266	7E-05	0.0048	yes	up	0.14	0.94	1.79	3.92	0.36	30.32	0.9567	11.53333333

Supplemental Table 2. Primers in quantitative PCR

Gene Name	sense (5'-3')	anti-sense (5'-3')
Gapdh	ACAGCCGCATCTTCTTGTGC	CACTTTGCCACTGCAAATGG
Gpx4	TGCTGTGCGGGGCTCT	CGATGTCCTTGGCTGAGAATT
TNF-α	GGAACACGTCGTGGGATAATG	GGCAGACTTTGGATGCTTCTT
IL-8	GGCATCTTCGTCCGTCCC	CCAACAGTAGCCTTCACCCAT
IL-6	ATGAAGTTCCTCTCTGCAAGAGAC	CACTAGGTTTGCCGAGTAGATCTC
IL-1α	TCGTCAGGCAGAAGTTTGTCA	CGACTTTGTTCTTTGGTGGC
Ppara	TGCCTGTCTGTCGGGATGT	GCGGGTTGTTGCTGGTCT
Pparg	ACAGTTGATTTCTCCAGCATTTC	CTACTTTGATCGCACTTTGGTATT
Ppargc1a	CTTTCTGGGTGGATTGAAGTGG	TCTTTGTGGCTTTTGCTGTTG
Ppargc1b	TCAGATGGAACCCCAAGCG	GGCACTCTACAATCTCACCGAAC
