# Supplementary material for: Pro-ferroptotic signaling promotes arterial aging via vascular smooth muscle cell senescence
Source: Nat Commun. 2024 Feb 16;15:1429. doi: 10.1038/s41467-024-45823-w (PMC10873425; doi:10.1038/s41467-024-45823-w)
Supplement: Supplementary file 3 — Reporting Summary [file 41467_2024_45823_MOESM3_ESM.pdf]

nature portfolio

Corresponding author(s): PEI WANG

Last updated by author(s): Jan 18, 2024

Reporting Summary

Nature Portfolio wishes to improve the reproducibility of the work that we publish. This form provides structure for consistency and transparency in reporting. For further information on Nature Portfolio policies, see our Editorial Policies and the Editorial Policy Checklist.

Statistics

For all statistical analyses, confirm that the following items are present in the figure legend, table legend, main text, or Methods section.

n/a	Confirmed	
☐	☒	The exact sample size (*n*) for each experimental group/condition, given as a discrete number and unit of measurement
☐	☒	A statement on whether measurements were taken from distinct samples or whether the same sample was measured repeatedly
☐	☒	The statistical test(s) used AND whether they are one- or two-sided<br>*Only common tests should be described solely by name; describe more complex techniques in the Methods section.*
☒	☐	A description of all covariates tested
☒	☐	A description of any assumptions or corrections, such as tests of normality and adjustment for multiple comparisons
☐	☒	A full description of the statistical parameters including central tendency (e.g. means) or other basic estimates (e.g. regression coefficient) AND variation (e.g. standard deviation) or associated estimates of uncertainty (e.g. confidence intervals)
☐	☒	For null hypothesis testing, the test statistic (e.g. *F*, *t*, *r*) with confidence intervals, effect sizes, degrees of freedom and *P* value noted<br>*Give P values as exact values whenever suitable.*
☒	☐	For Bayesian analysis, information on the choice of priors and Markov chain Monte Carlo settings
☒	☐	For hierarchical and complex designs, identification of the appropriate level for tests and full reporting of outcomes
☒	☐	Estimates of effect sizes (e.g. Cohen's *d*, Pearson's *r*), indicating how they were calculated

Our web collection on statistics for biologists contains articles on many of the points above.

Software and code

Policy information about availability of computer code

Data collection	Vevo 2100 high-frequency ultrasound system (VisualSonic, Toronto, ON, Canada); CFX96 Real Time PCR Detection System (BioRad); Odyssey system (Li-Cor Biosciences); Automatic biochemical analyzer (Beckman-Coulter, USA); Electron microscope (Hitachi, H-800, Tokyo, Japan), Leica Application Suite (Leica); Flow cytometry (FACS Calibur, BD Biosciences, Corp., San Jose, CA); FV1000 confocal microscopy (Olympus); Illumina Hiseq2000 platform.
Data analysis	GraphPad Prism 8.0.2 (GraphPad Software, California, USA); Excel 2017 (Microsoft); FlowJo software (version 14.0.0); Image Studio Lite (Li-COR); ImageJ 1.52p (NIH, USA).

For manuscripts utilizing custom algorithms or software that are central to the research but not yet described in published literature, software must be made available to editors and reviewers. We strongly encourage code deposition in a community repository (e.g. GitHub). See the Nature Portfolio guidelines for submitting code & software for further information.

Data

Policy information about availability of data

All manuscripts must include a data availability statement. This statement should provide the following information, where applicable:

- Accession codes, unique identifiers, or web links for publicly available datasets
- A description of any restrictions on data availability
- For clinical datasets or third party data, please ensure that the statement adheres to our policy

Source data for Figures 1–10 and Supplementary Data Figures 1–15 are provided as a Source Datafile. The raw RNA-sequencing data of aortae tissue from mice has been deposited in the NCBI Sequence Read Archive (SRA) database (PRJNA907269, https://www.ncbi.nlm.nih.gov/sra/?term=PRJNA907269). Public gene expression data (GSE1011) was accessible from Bioproject Database (https://www.ncbi.nlm.nih.gov/bioproject/PRJNA87077).

Research involving human participants, their data, or biological material

Policy information about studies with human participants or human data. See also policy information about sex, gender (identity/presentation), and sexual orientation and race, ethnicity and racism.

Reporting on sex and gender	We did not report the sex and gender in human data.<br>Sex was not considered in the study design.
Reporting on race, ethnicity, or other socially relevant groupings	We have provided sufficient information about the human participants in the Method section.
Population characteristics	Two populations of humans were included.<br>Population 1 consisted of patients who underwent carotid aneurysm resection. Middle-aged (< 45-y-old) and elderly (> 65-y-old) patients were included.<br>Population 2 comprised 80 participants who voluntarily attended regular health examinations. A total of 73 people were included in present analysis with the response rate 91.3%. The detailed information regarding these populations is presented in the Method section.
Recruitment	For population 1, middle-aged (<45 years old, n=6) and elderly (>65 years old, n=6) patients were recruited.<br><br>For population 2, the inclusion criteria were: 1) over 18 years old; 2) stable on drugs and lifestyle, which means no significant changes in medication, diet, or exercise habit for at least one month; 3) willing and able to complete the study with written informed consent. The exclusion criteria were: 1) severe organ dysfunctions including heart (NYHA III-IV), kidney (CKD 4~5), or liver (Child-Pugh score >6); 2) musculoskeletal diseases; 3) physical disability relevant to routine exercise; 4) severe uncontrolled hypertension (resting systolic/diastolic blood pressure > 180/110 mmHg) or diabetes (HbA1c > 10.0%); 5) recent cerebral or cardiovascular events including myocardial infarction and stroke; 6) low life expectancy like malignant tumor.<br><br>The criteria are all objective factors, and there was no self-selection bias or any other biases.
Ethics oversight	Population 1: Shanghai Changzheng Hospital. This study was performed according to the requirements of the Ethical Committee of Changzheng Hospital and the Declaration of Helsinki. All participants gave written informed consent.<br><br>Population 2: Shanghai Tenth People's Hospital. The protocol of the study was conducted in accordance with the Declaration of Helsinki and was approved by the Ethics Committee of Shanghai Tenth People's Hospital affiliated Tongji University Medical School. All participants gave written informed consent.

Note that full information on the approval of the study protocol must also be provided in the manuscript.

Field-specific reporting

Please select the one below that is the best fit for your research. If you are not sure, read the appropriate sections before making your selection.

☒ Life sciences ☐ Behavioural & social sciences ☐ Ecological, evolutionary & environmental sciences

For a reference copy of the document with all sections, see nature.com/documents/nr-reporting-summary-flat.pdf

Life sciences study design

All studies must disclose on these points even when the disclosure is negative.

Sample size	In in vitro (cultured cell) study, we choose > 3 biological repeats in each group for treatment. Sample sizes were based on our previous experience, providing enough statistical power to detect the usually strong effects observed in our experiments. A ll sample sizes are clearly described in the manuscript or the figure legend.<br><br>In animal study, we choose > 4 mice in each group for treatment. Sample sizes were based on our previous experience, providing enough statistical power to detect the usually strong effects observed in our experiments. All sample sizes are clearly described in the manuscript or

the figure legend.

In human study, sample sizes were based on our previous experience, providing enough statistical power to detect the usually strong effects observed in our experiments.

Data exclusions	In population 2 of human study, 80 voluntarily-attended participants who underwent regular health examination in Shanghai Tenth People's Hospital affiliated Tongji University Medical School from Dec 2018 to Dec 2019 were included. Finally, 73 people were included in present analysis with the response rate 91.3%. The exclusion criteria were: 1) severe organ dysfunctions including heart (NYHA III-IV), kidney (CKD 4~5), or liver (Child-Pugh score >6); 2) musculoskeletal diseases; 3) physical disability relevant to routine exercise; 4) severe uncontrolled hypertension (resting systolic/diastolic blood pressure > 180/110 mmHg) or diabetes (HbA1c > 10.0%); 5) recent cerebral or cardiovascular events including myocardial infarction and stroke; 6) low life expectancy like malignant tumor.
Replication	All the experiments were performed in triplicates with a good reproducibility of the experimental findings.
Randomization	In experiments with young and aged mice, the subjects were grouped based on their age. In other experiments, mice or cells were randomly assigned to various groups with different treatments.
Blinding	The data were analyzed by groups. In experiments involving young and aged mice, the investigators were not blinded, as these two types of mice have totally distinct appearances. However, in other experiments, the investigators were blinded.

Reporting for specific materials, systems and methods

We require information from authors about some types of materials, experimental systems and methods used in many studies. Here, indicate whether each material, system or method listed is relevant to your study. If you are not sure if a list item applies to your research, read the appropriate section before selecting a response.

Materials & experimental systems

n/a	Involved in the study
☐	☒ Antibodies
☐	☒ Eukaryotic cell lines
☒	☐ Palaeontology and archaeology
☐	☒ Animals and other organisms
☒	☐ Clinical data
☒	☐ Dual use research of concern
☒	☐ Plants

Methods

n/a	Involved in the study
☒	☐ ChIP-seq
☒	☐ Flow cytometry
☒	☐ MRI-based neuroimaging

Antibodies

Antibodies used

Immunoblotting:
anti-GPX4, #ab125066, Abcam, 1:2000; anti-ACSL4, #sc-365230, Santa Cruz Biotechnology, 1:2000; anti-ALOX15, #PA5-15065, Invitrogen, 1:2000; anti-TFR1, #10084-2-AP, Proteintech, 1:1000; anti-MDA, #ab6463, Abcam, 1:4000; anti-4-HNE, #ab46545,Abcam, 1:4000; anti-p53, #A5761, Abclonal, 1:2000; anti-p16INK4A, #sc-1661, Santa Cruz Biotechnology, 1:1000; anti-p21WAF1, #sc-817, Santa Cruz Biotechnology, 1:1000; anti-gammaH2A.XSer139, #ab81299, Abcam, 1:2000; anti-H2A.X, #A11412, ABclonal, 1:2000; anti-PARP-1, #13371-1-AP, Proteintech, 1:2000; anti-CD38, #sc-374650, Santa Cruz Biotechnology, 1:2000; anti-IL-1beta, # 16765-1-AP, Proteintech, 1:2000; anti-HMGB1, #ab18256, Abcam, 1:2000; anti-LC3, #14600-1-AP, Proteintech, 1:1000; anti-NCOA4, #sc-373739, Santa Cruz Biotechnology, 1:1000; anti-FTH1, #4393, Cell Signaling Technology, 1:2000; anti-PPARgamma, #16643-1-AP, #60004-1-Ig, Proteintech, 1:1000; anti-tubulin, #AC008, Abclonal, 1:4000.

Immunofluorescence and immunohistochemistry:
anti-GPX4, #ab125066, Abcam, 1:500; anti-ACSL4, #sc-365230, Santa Cruz Biotechnology, 1:200; anti-ALOX15, #PA5-15065, Invitrogen, 1:500; anti-MDA, #ab6463, Abcam, 1:1000; anti-4-HNE, #ab46545,Abcam, 1:1000; anti-p53, #A5761, Abclonal, 1:1000; anti-p16INK4A, #sc-1661, Santa Cruz Biotechnology, 1:200; anti-p21WAF1, #sc-817, Santa Cruz Biotechnology, 1:200; anti-gammaH2A.XSer139, #ab81299, Abcam, 1:500; anti-IL-1beta, # 16765-1-AP, Proteintech, 1:500; anti-HMGB1, #ab18256, Abcam, 1:500; anti-NCOA4, #sc-373739, Santa Cruz Biotechnology, 1:200; anti-FTH1, #4393, Cell Signaling Technology, 1:500; anti-a-SMA, #14395-1-AP, Proteintech, 1:500.

Immunoprecipitation
anti-FTH1, #4393, Cell Signaling Technology, 1:100; anti-HA-tag, #ab9110, Abcam, 1:200; anti-Flag-tag, #ab205606, Abcam, 1:200.

Validation

All antibodies were purchased from commercial sources and were validated by the vendors.

Eukaryotic cell lines

Policy information about cell lines and Sex and Gender in Research

Cell line source(s)	ATCC (MOVAS: CRL-2797)

Authentication	The cell line was purchased from an authenticated vendor of ATCC.
Mycoplasma contamination	The cells were tested negative for mycoplasma contamination.
Commonly misidentified lines (See ICLAC register)	None

Animals and other research organisms

Policy information about studies involving animals; ARRIVE guidelines recommended for reporting animal research, and Sex and Gender in Research

Laboratory animals	C57BL/6J mice were obtained from Sino-British SIPPR/BK Lab Animal Ltd. (Shanghai, China). The knockin mouse stains harboring GPX4 at ROSA26 locus (R26-GPX4, project No. KICMS181221LY4) was generated by Cas9/CRISPR-mediated genome editing (Cyagen Biosciences, Santa Clara, CA, USA)<br><br>The mice were bred and housed under specific pathogen free conditions in the central animal facility. All mice were housed at a temperature of 21°-23°C with relative humidity of 35%-65% and 12h light/dark cycle in individually ventilated cages with access to water and standard chow diet.
Wild animals	This study did not use wild animals.
Reporting on sex	The aging process of blood vessels is influenced by sex hormones. To mitigate this interference factor, only male mice were utilized.
Field-collected samples	This study did not use field-collected samples.
Ethics oversight	All operations in mice were approved by the Animal Care and Use Committee of Naval Medical University and followed the Principles of Laboratory Animal Care published by the National Institutes of Health (NIH publication 86-23 revised 1985) and ARRIVE guidelines.

Note that full information on the approval of the study protocol must also be provided in the manuscript.
