# Supplementary material for: Pro-ferroptotic signaling promotes arterial aging via vascular smooth muscle cell senescence
Source: Nat Commun. 2024 Feb 16;15:1429. doi: 10.1038/s41467-024-45823-w (PMC10873425; doi:10.1038/s41467-024-45823-w)
Supplement: Supplementary file 4 — Source Data [file 41467_2024_45823_MOESM4_ESM.zip › Uncropped gels.pdf]

Fig. 1B

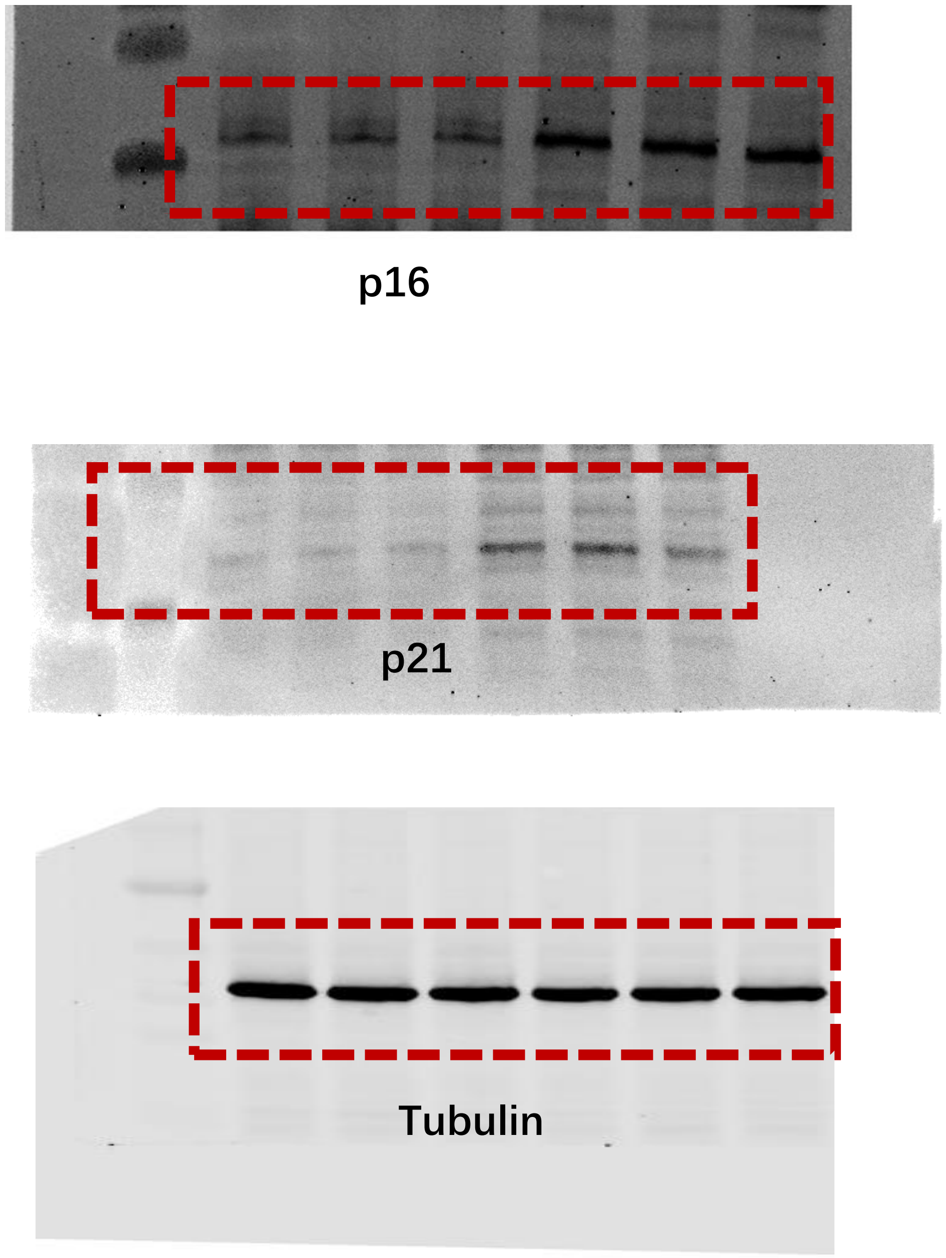


Fig. 1D

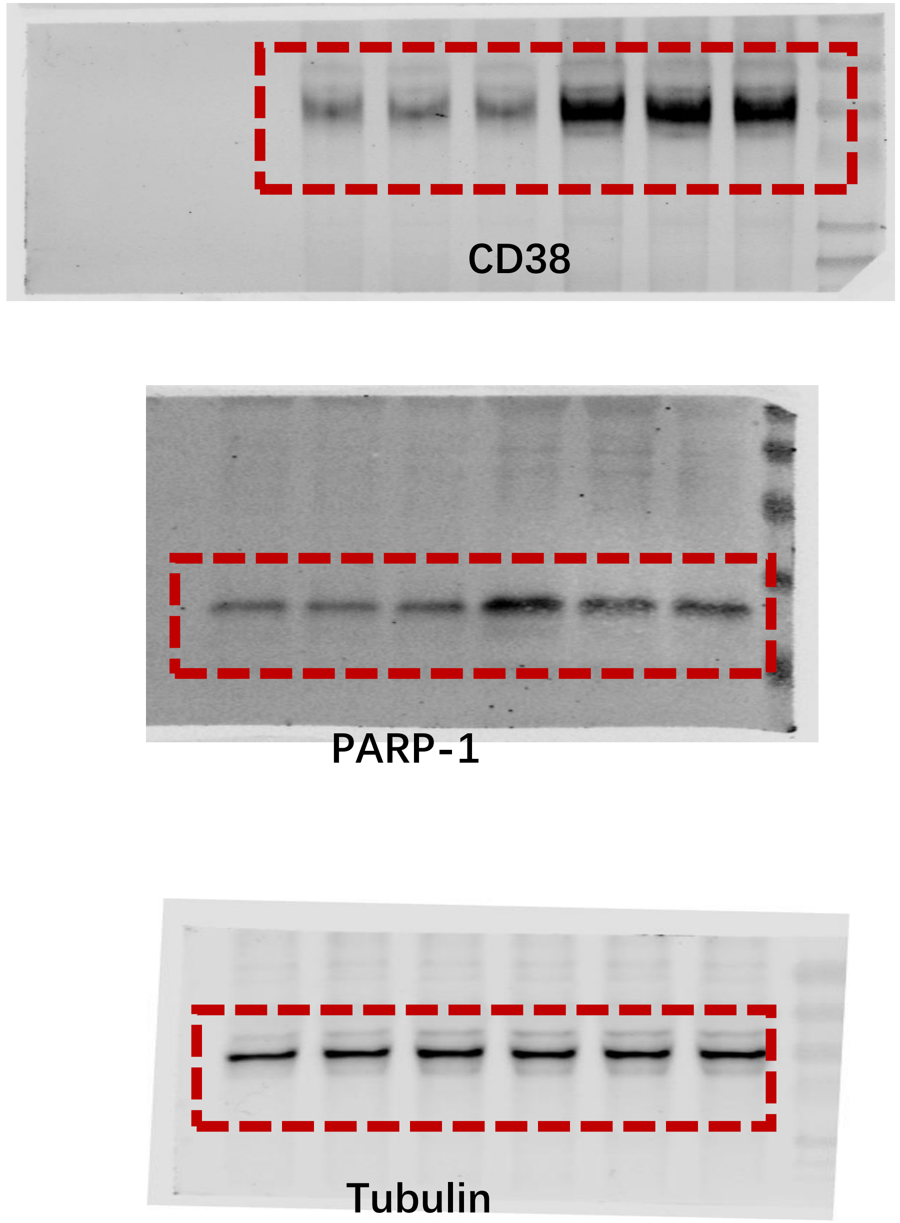

Fig. 1E

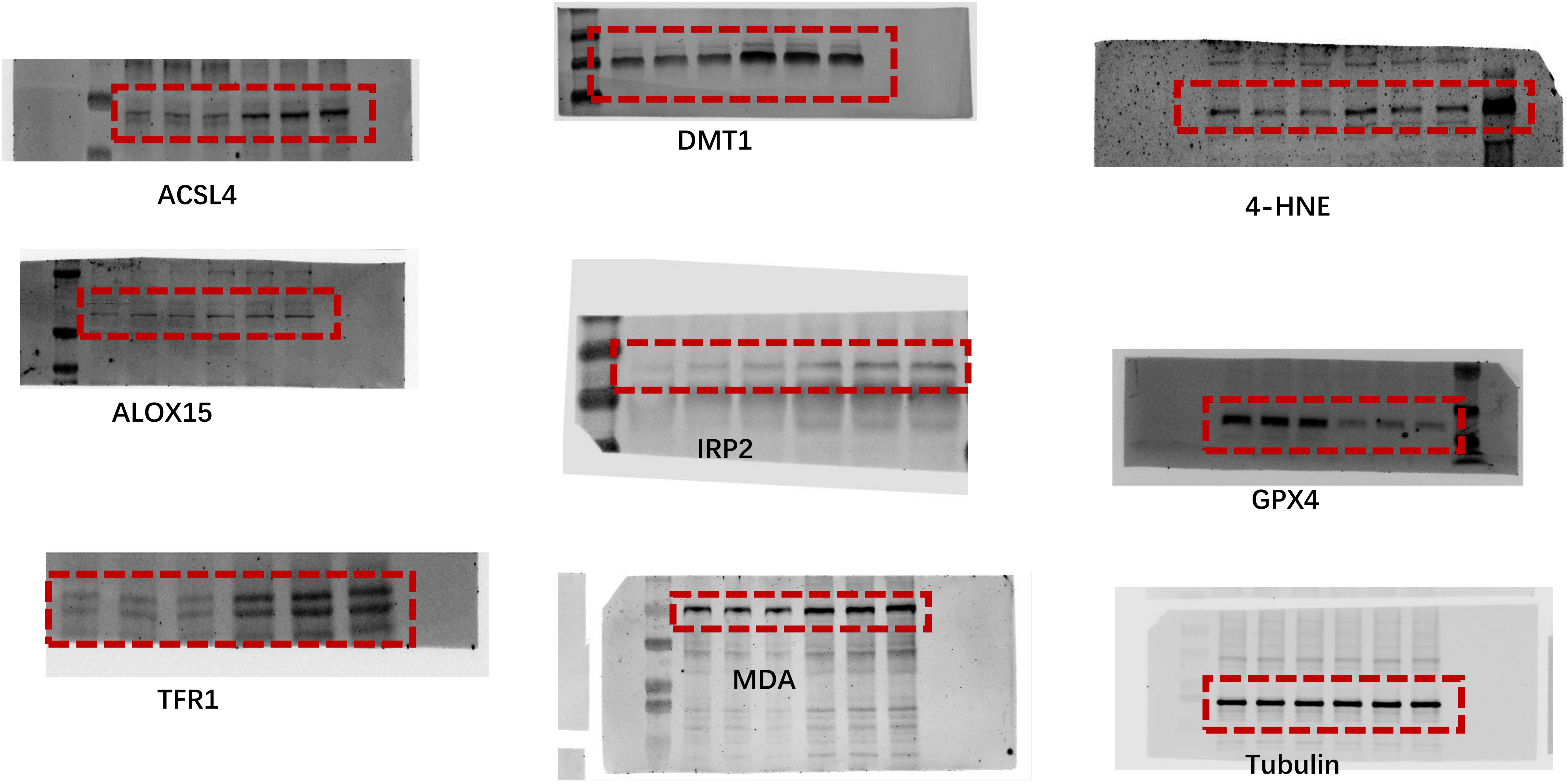

Fig. 1K

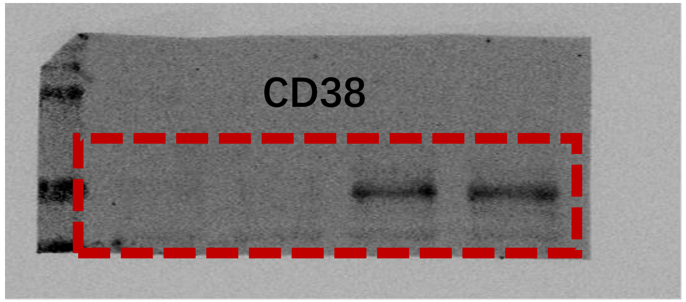


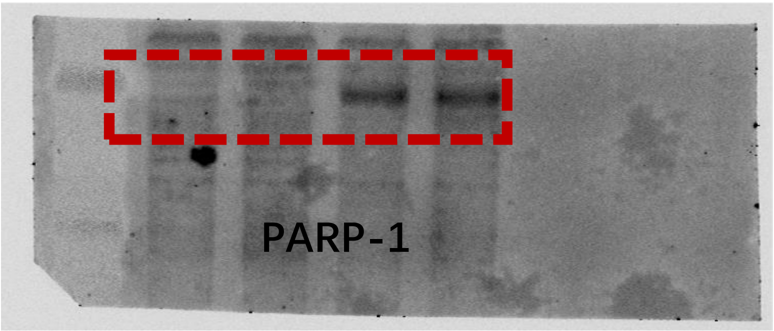


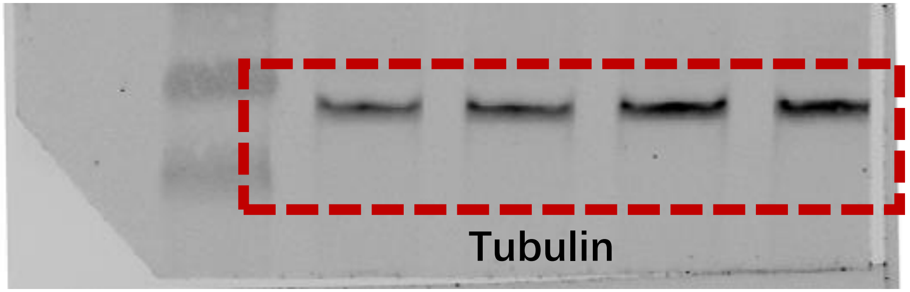

Fig. 2D

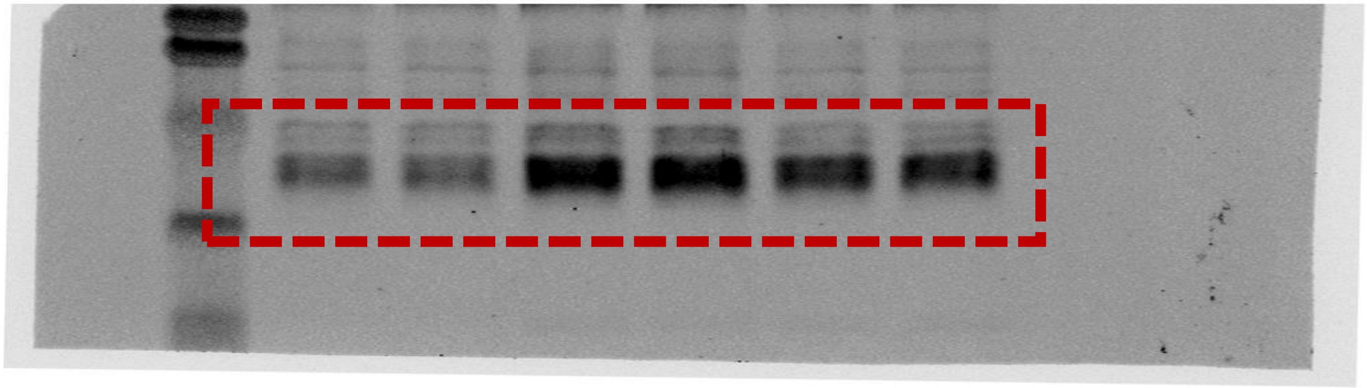

HMGB1

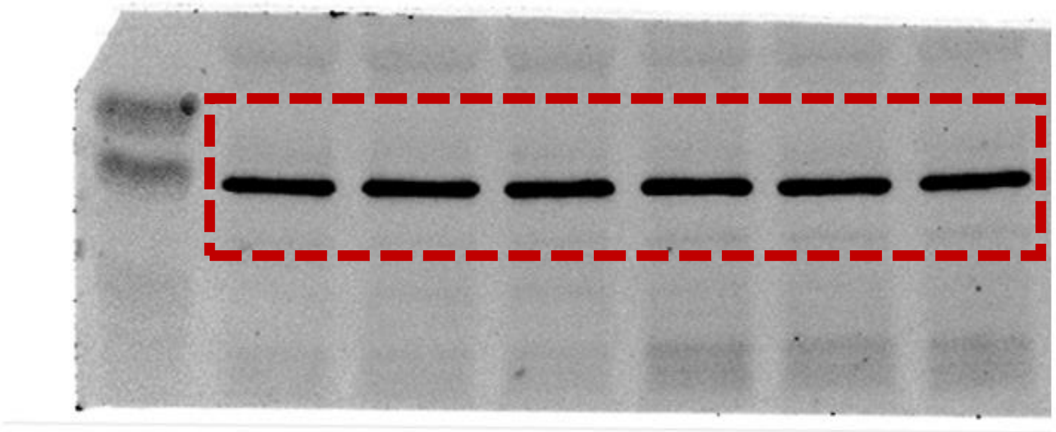

Tubulin

Fig. 2E

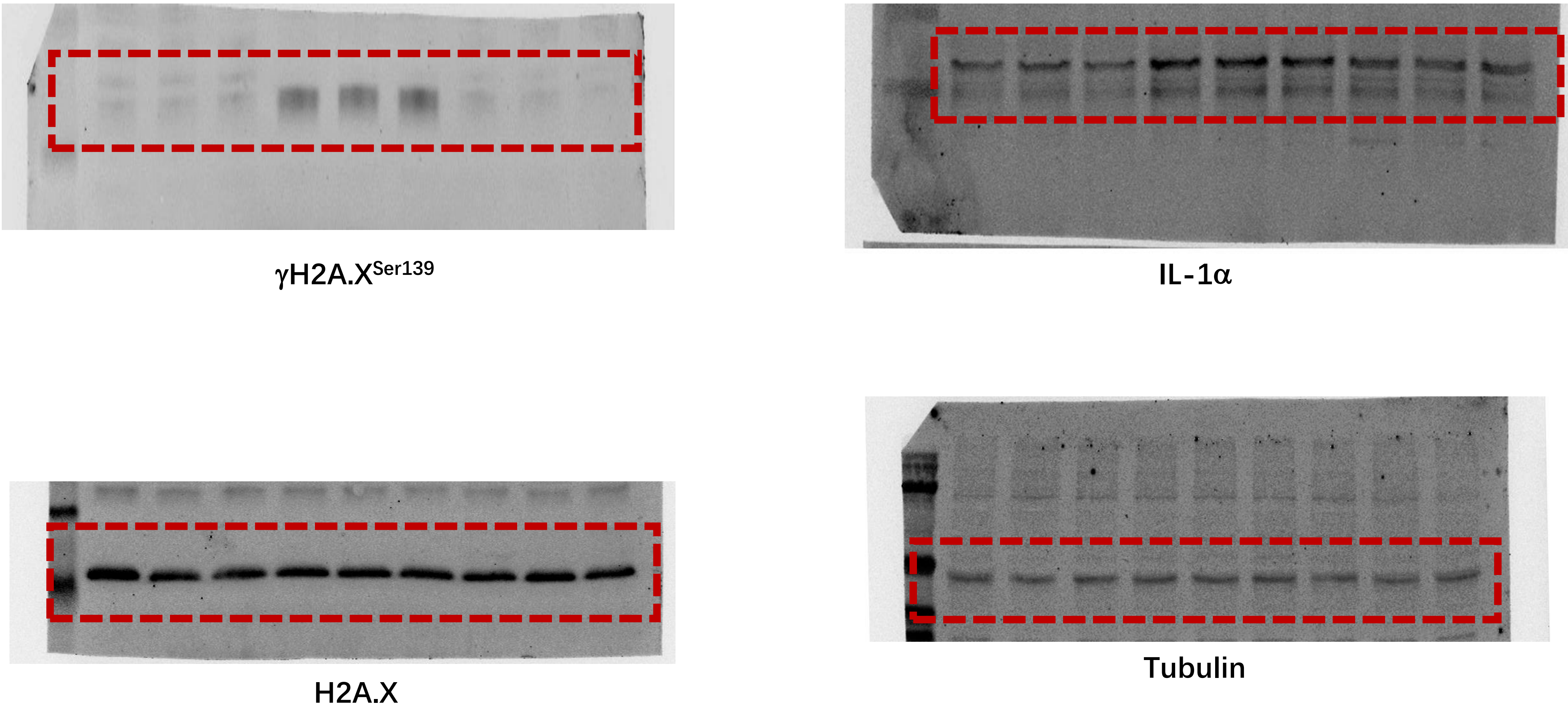

Fig. 2G

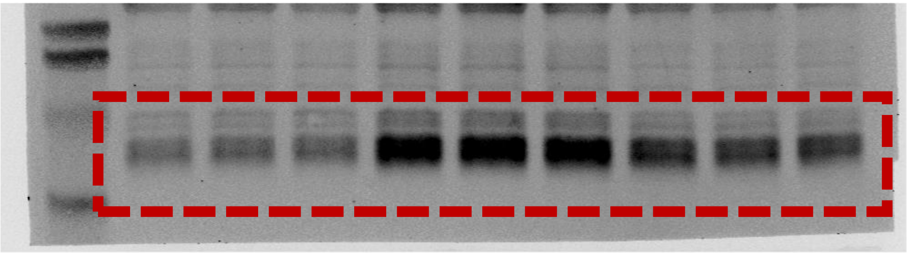

CD38

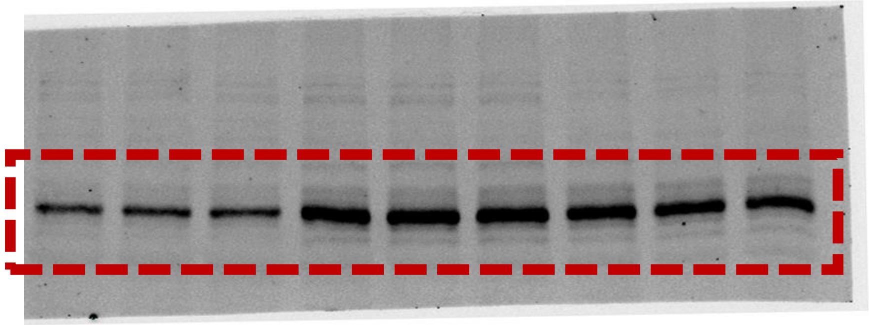

PARP-1

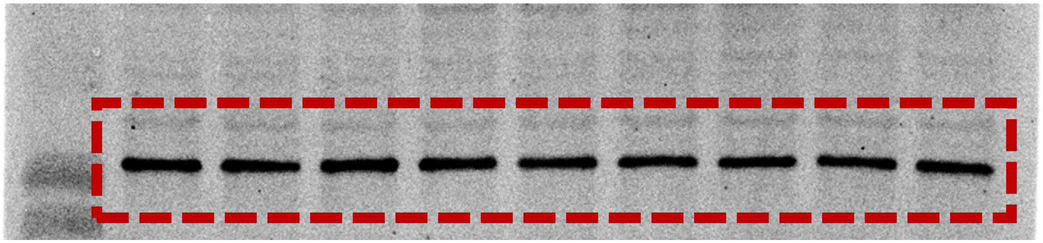

Tubulin

Fig. 3A

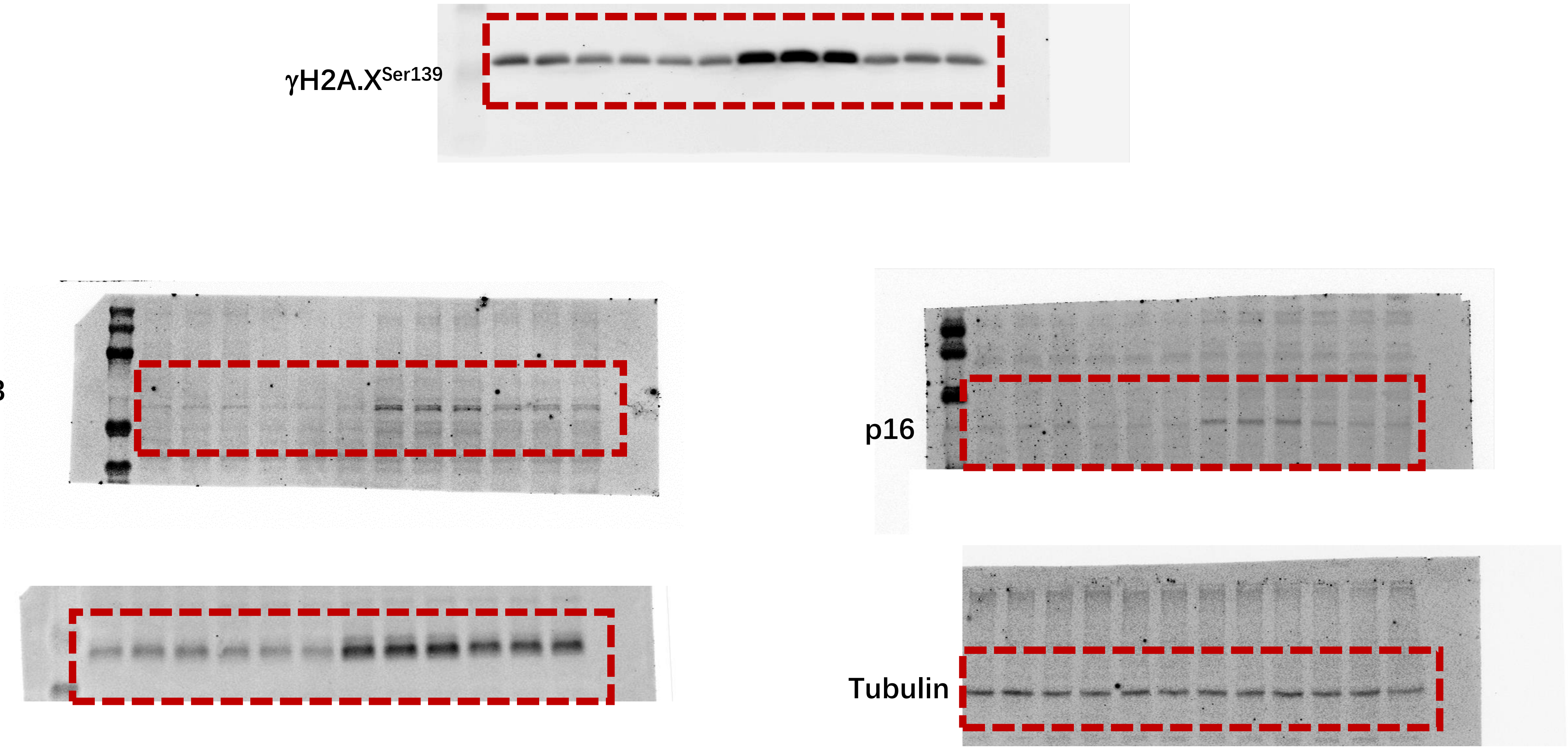

Fig. 3C

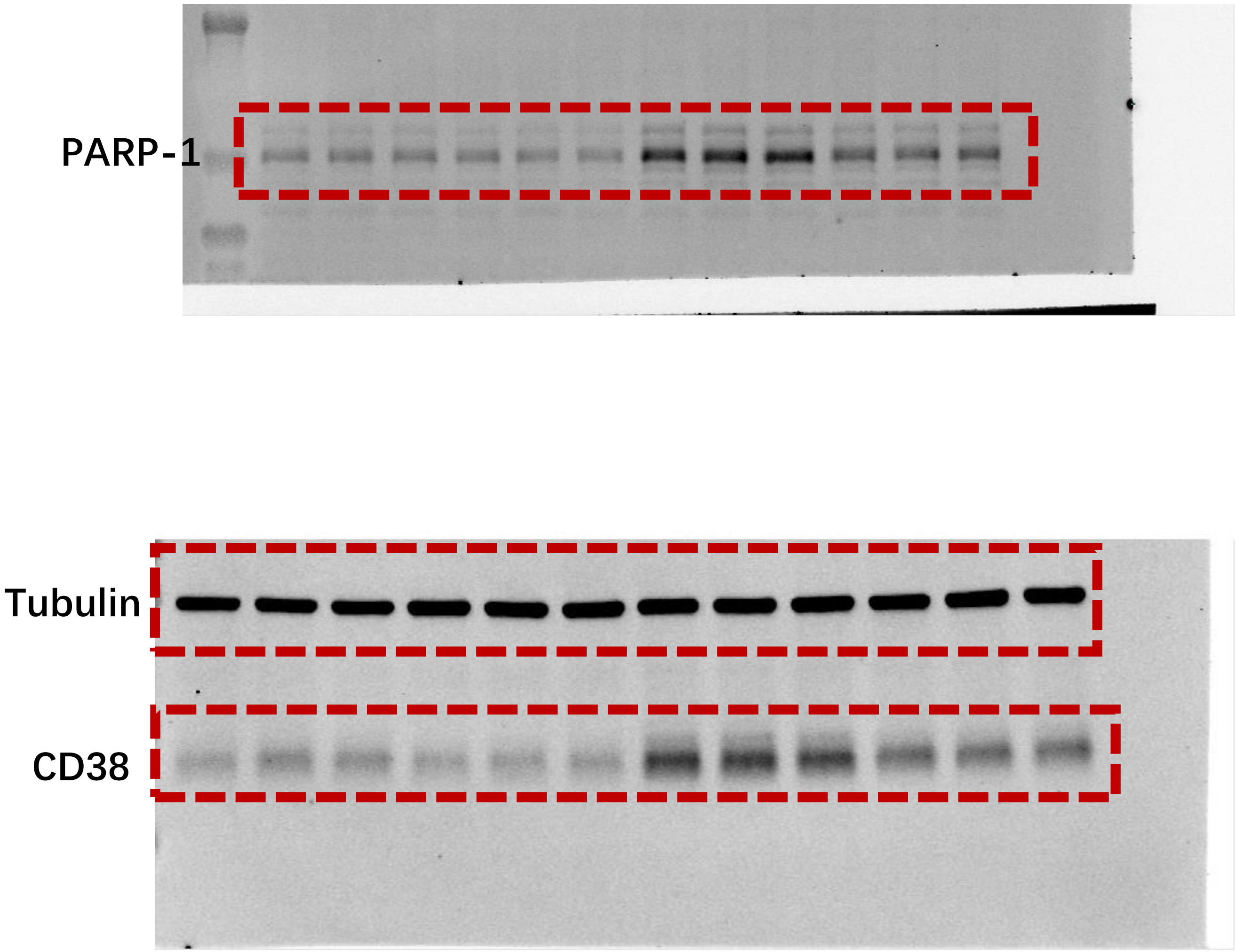

Fig. 3F

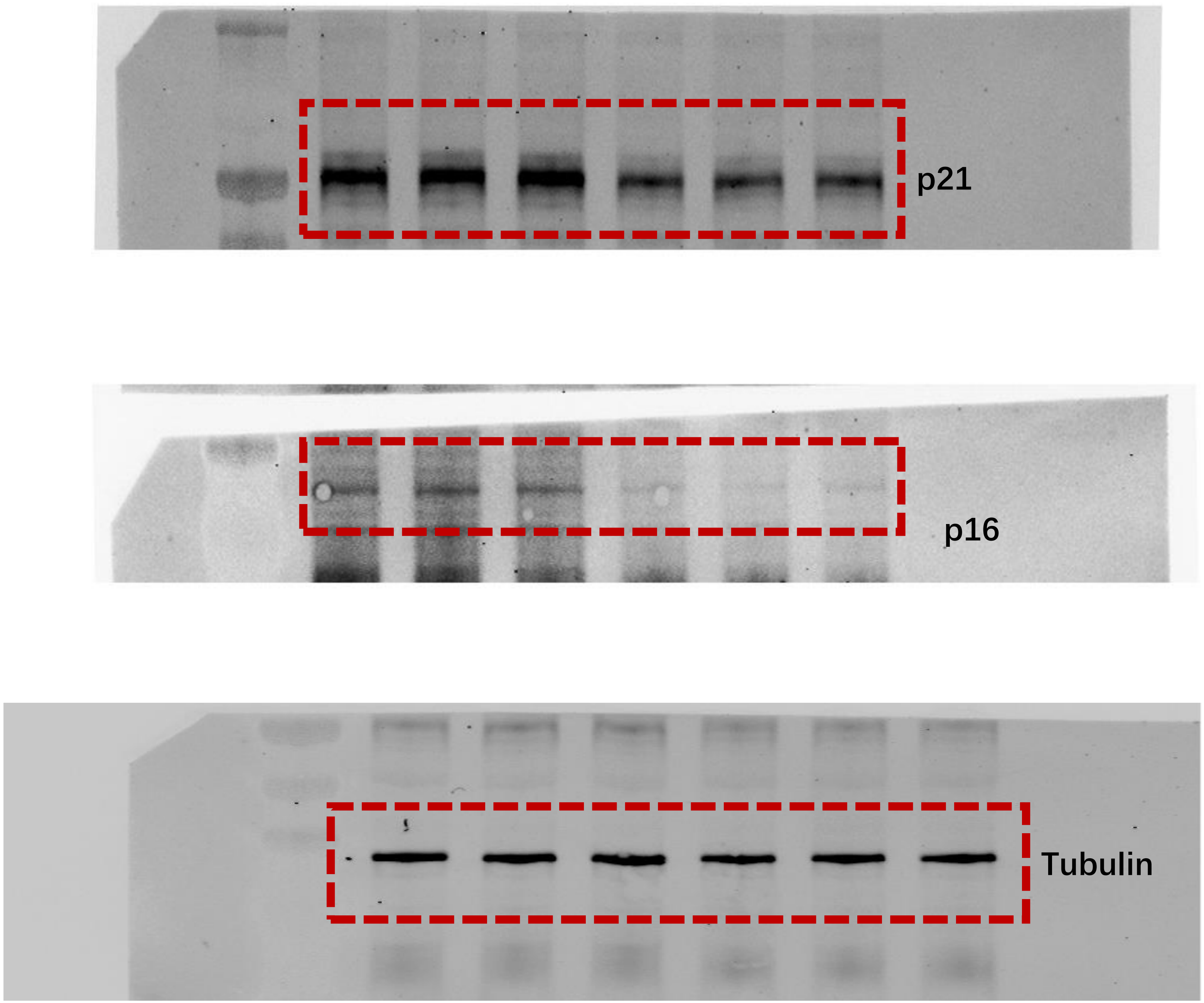

Fig. 3I

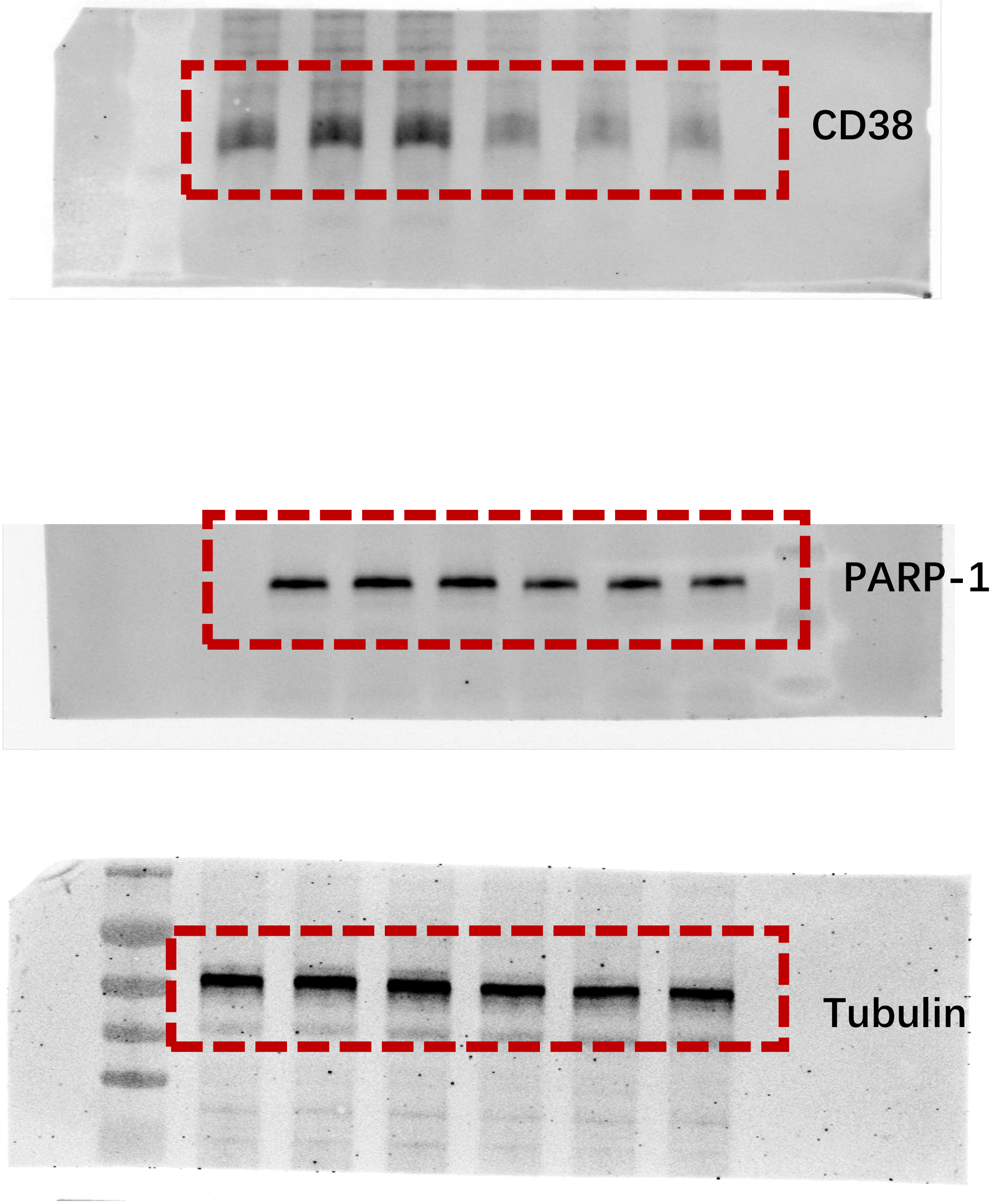

Fig. 4D

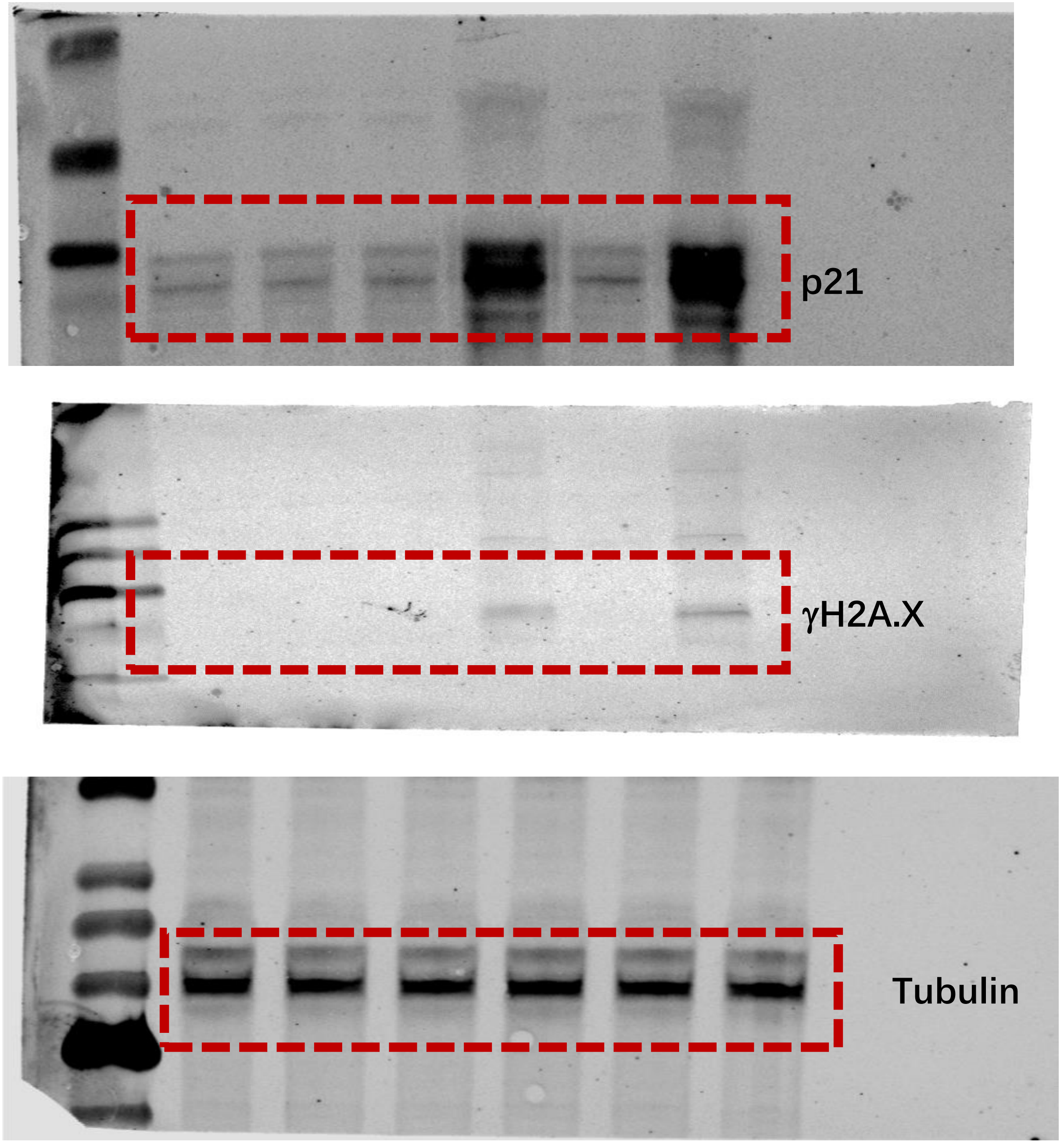

## Fig. 4H

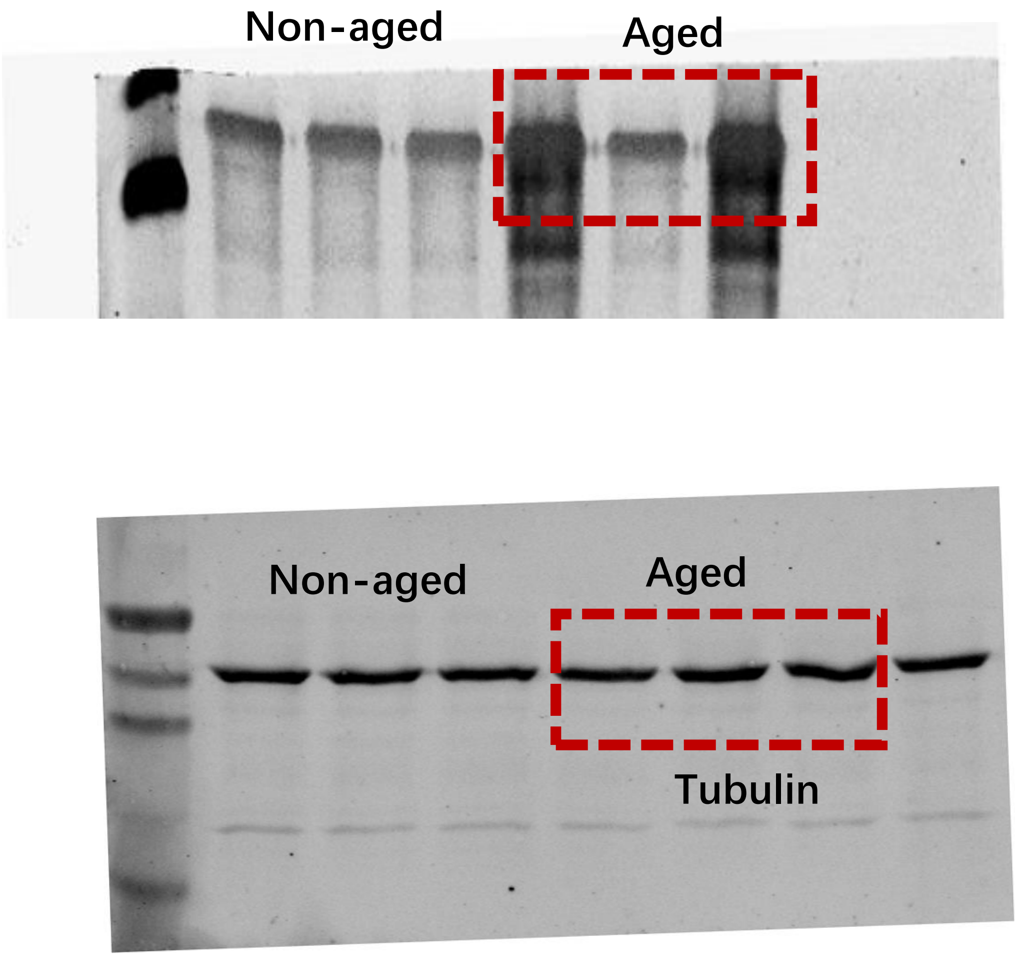


## Fig. 4K

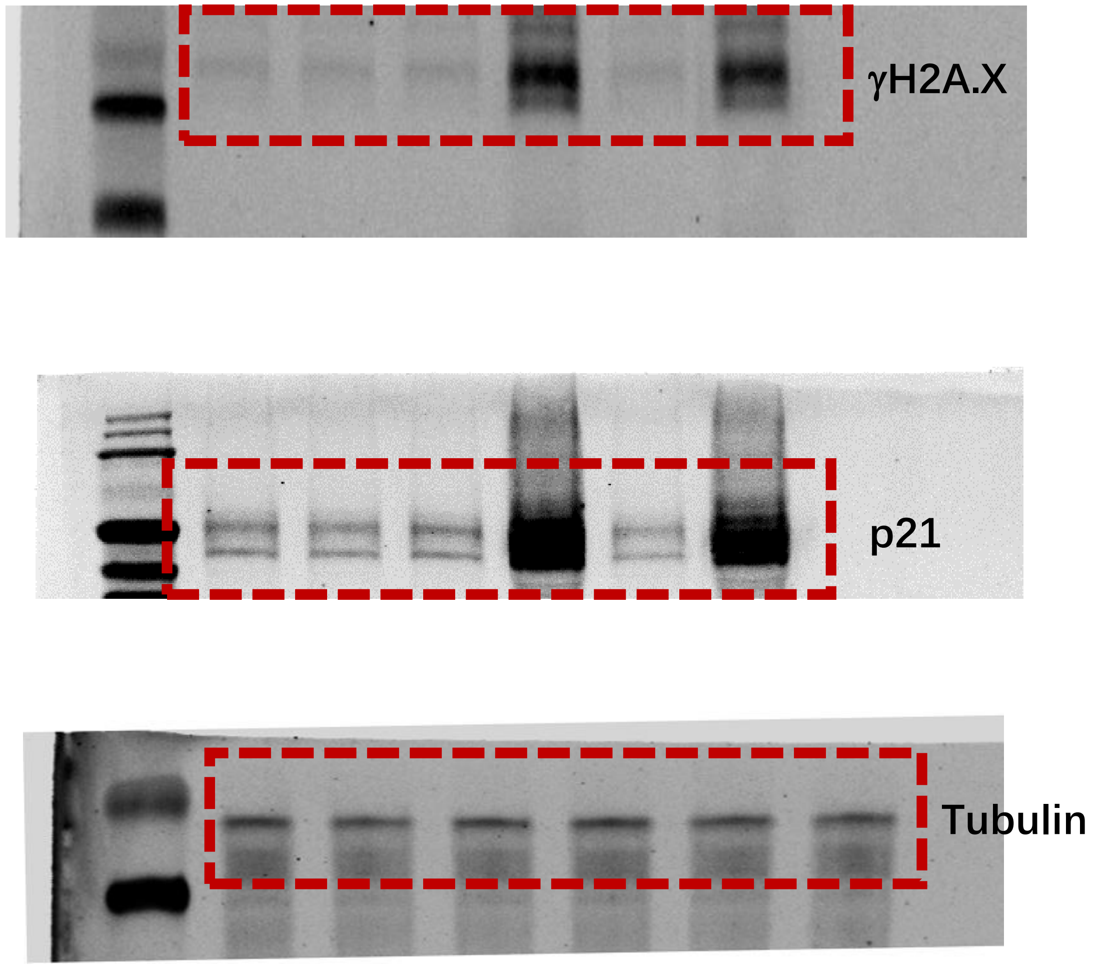

Fig. 4M

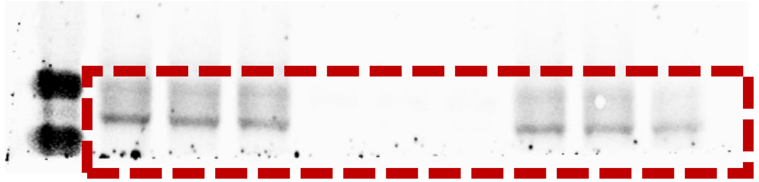

CD38

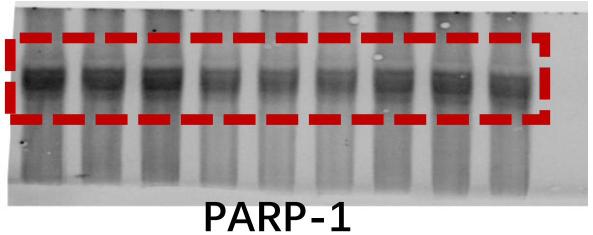

PARP-1

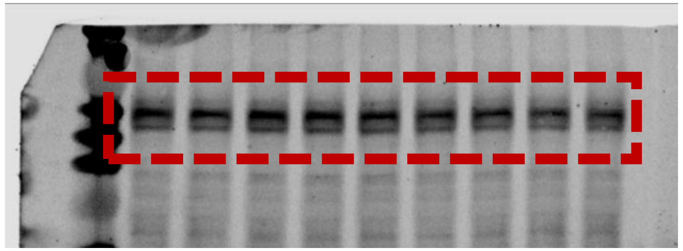

Tubulin

Fig. 5E

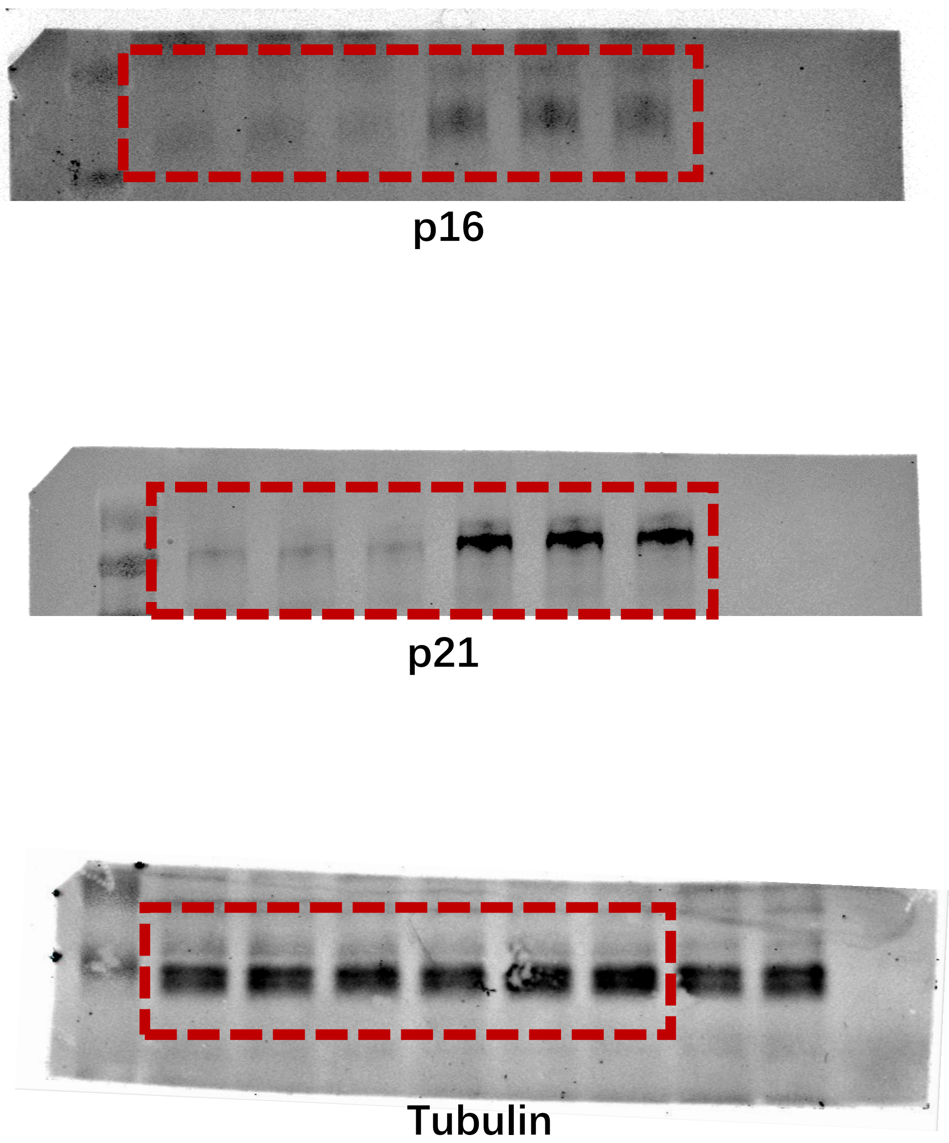


Fig. 5H

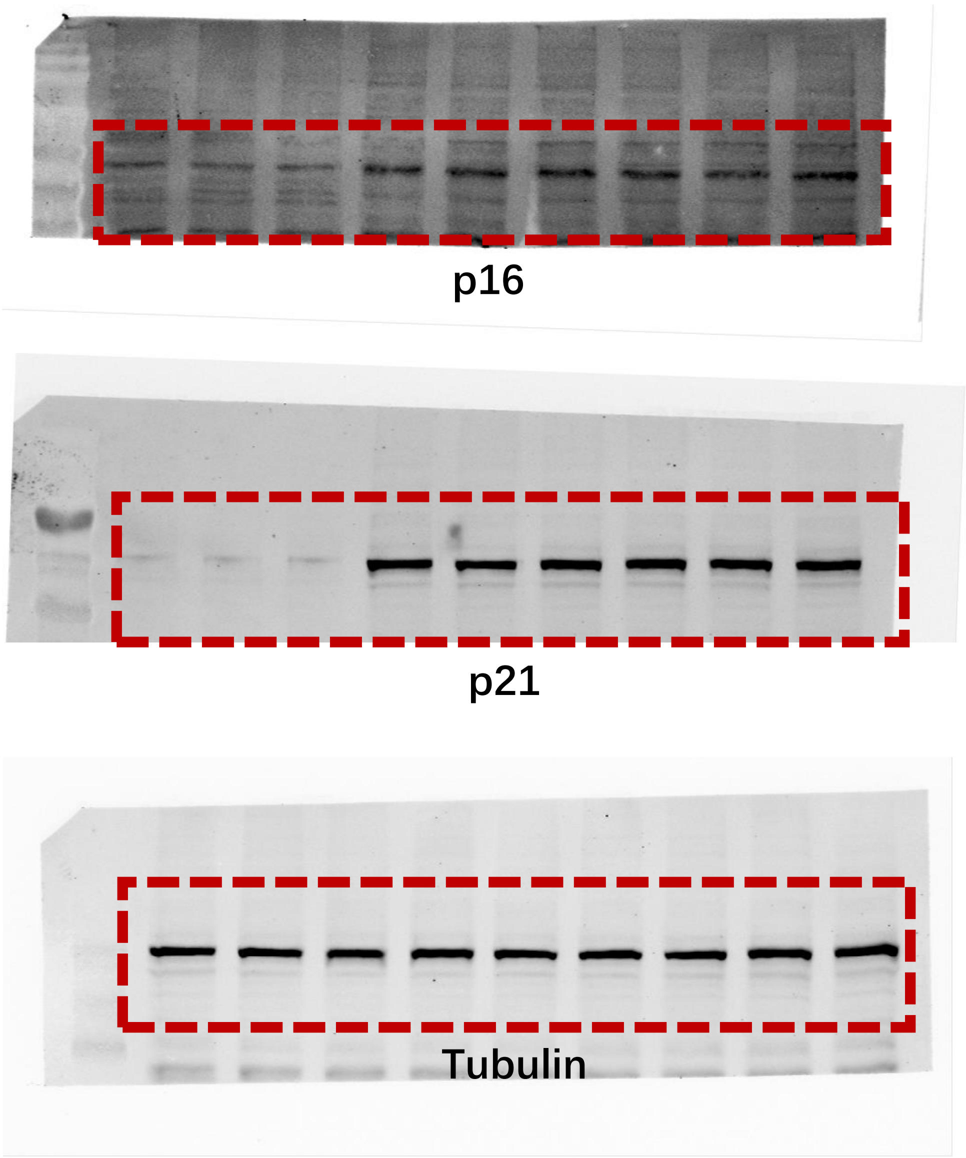

Fig. 6B

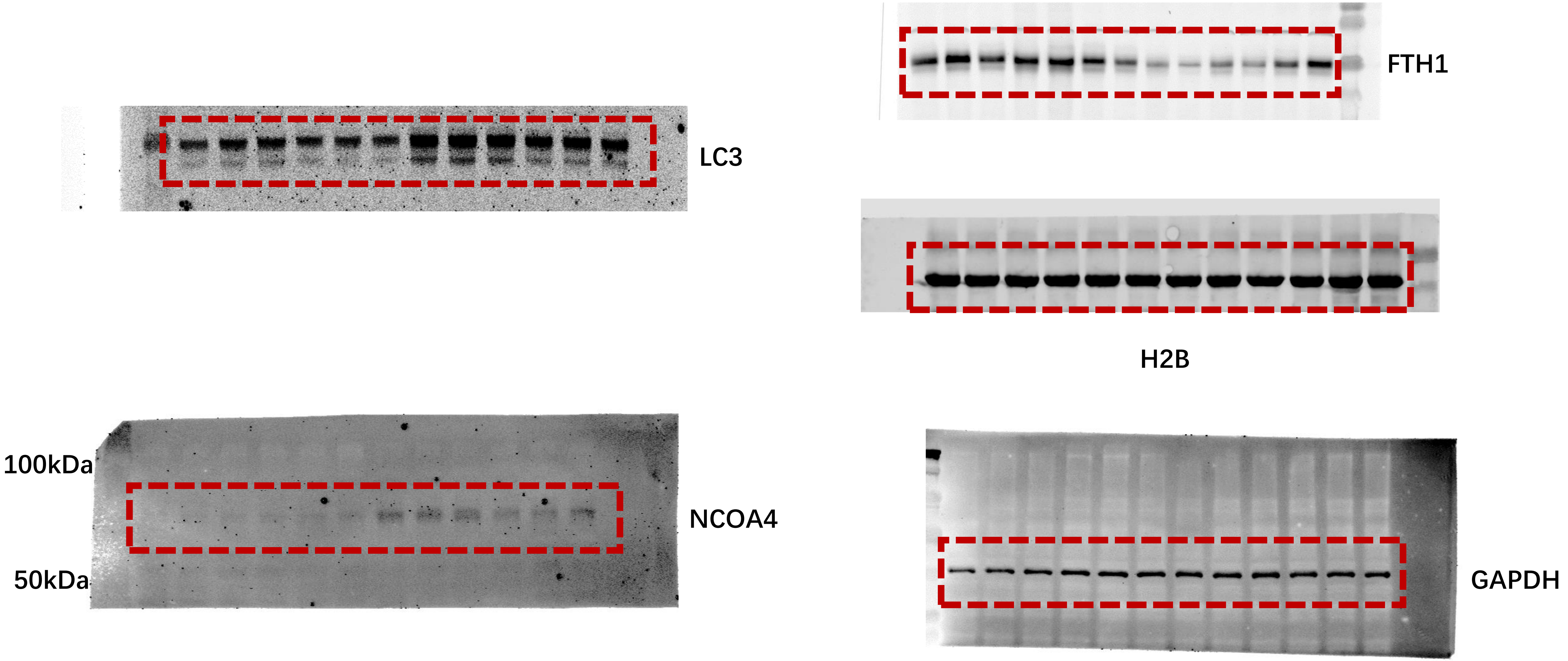

Fig. 6C

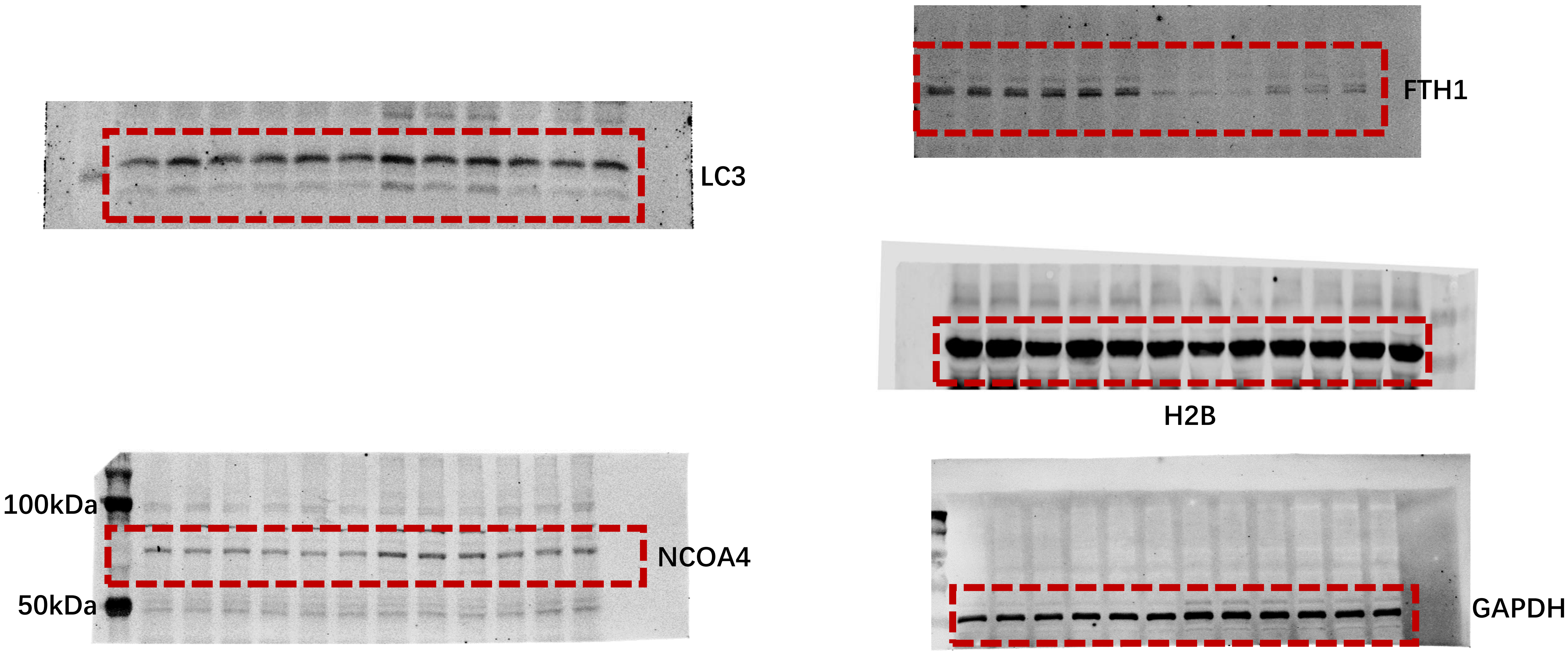

Fig. 7A

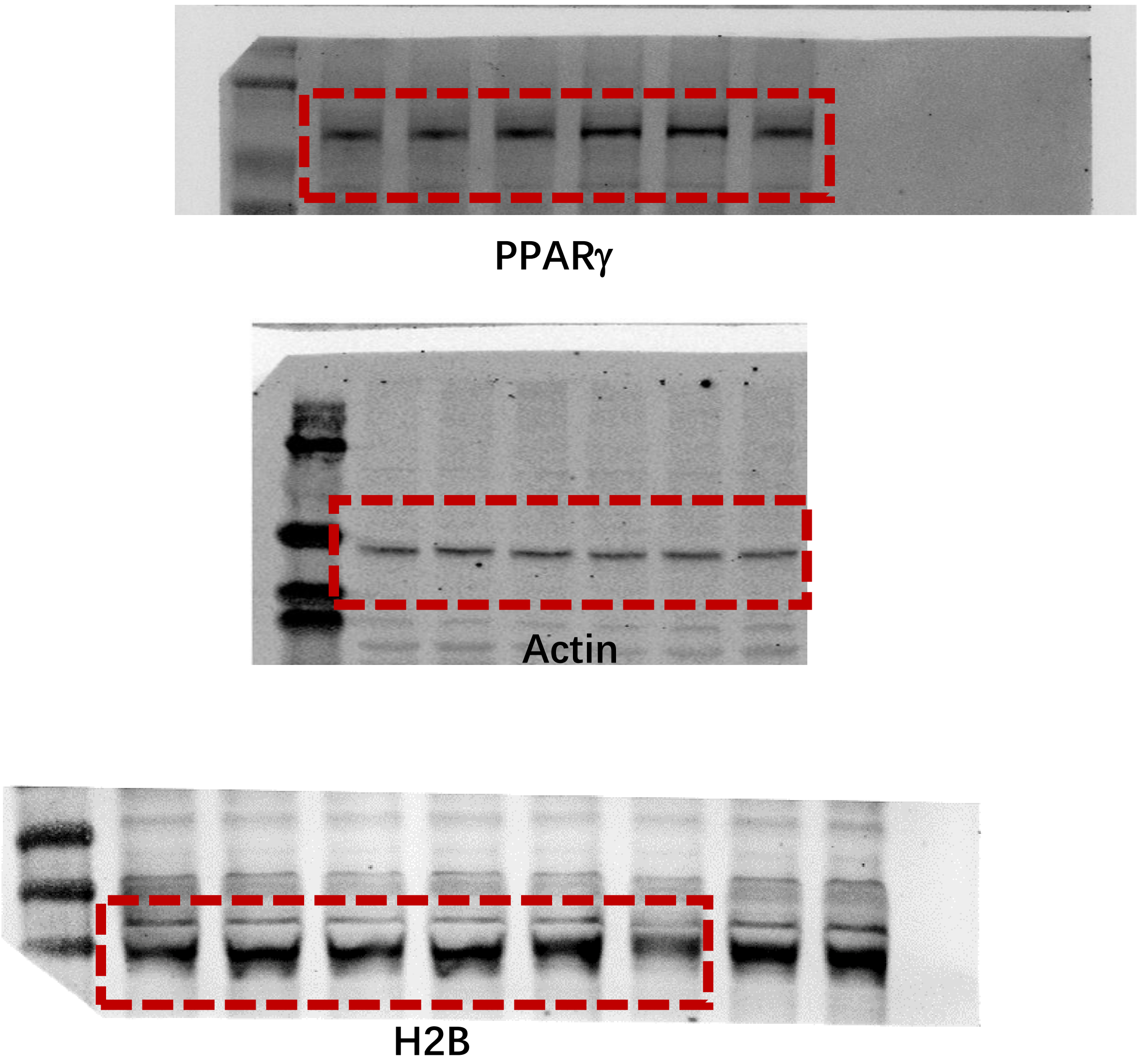


Fig. 7B

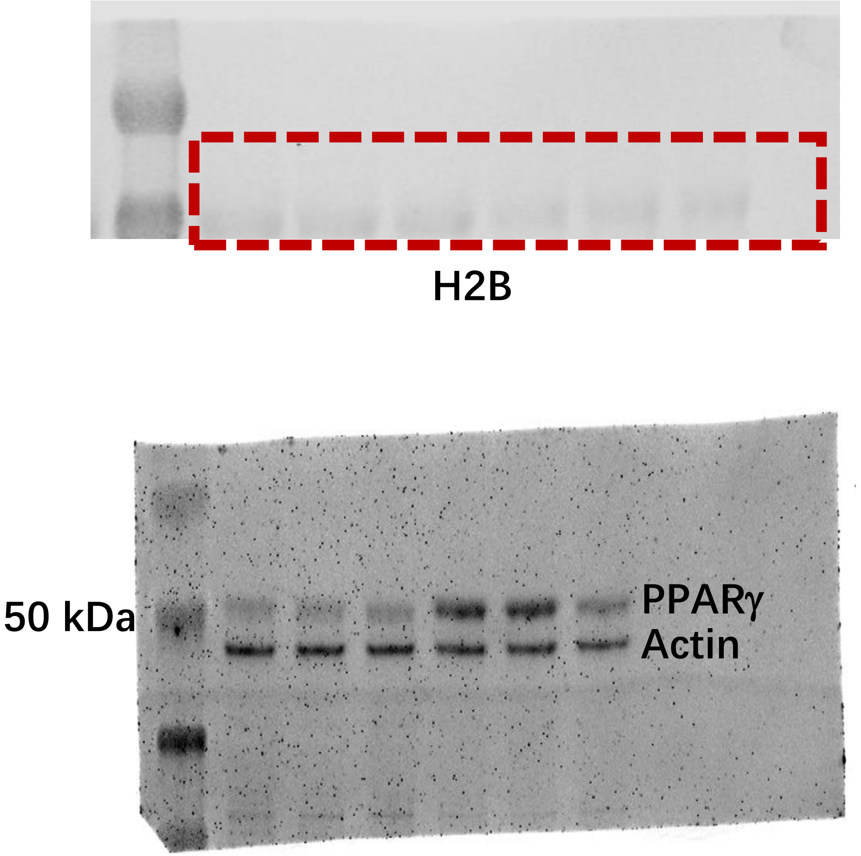

Fig. 7C

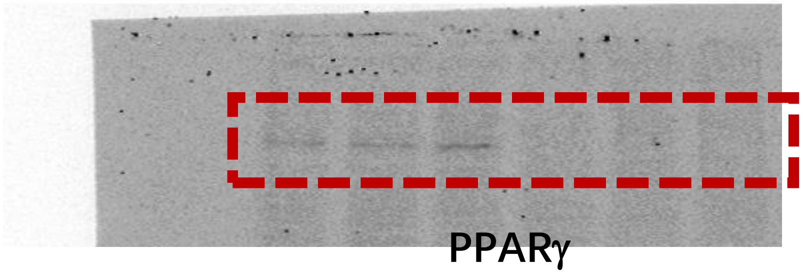


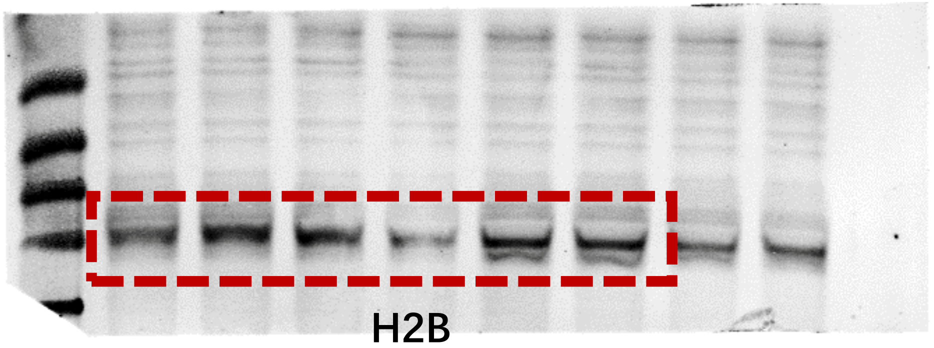


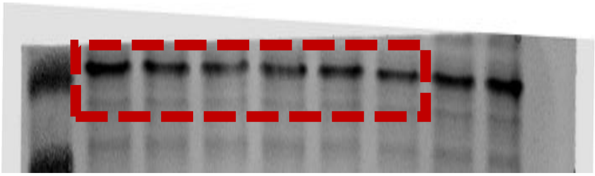


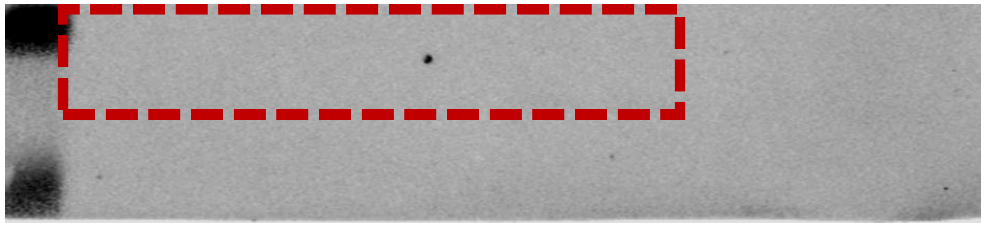

Fig. 7E

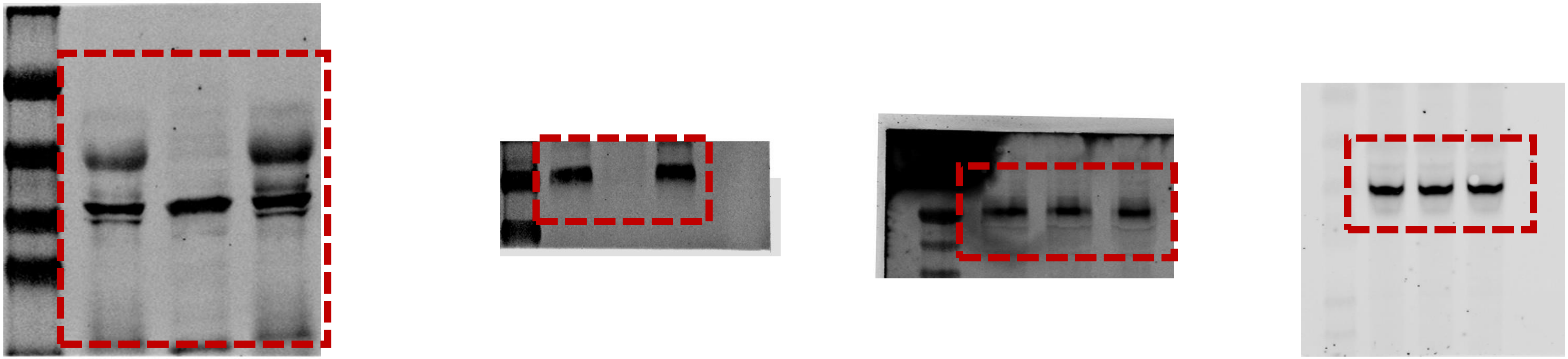

Fig. 7I

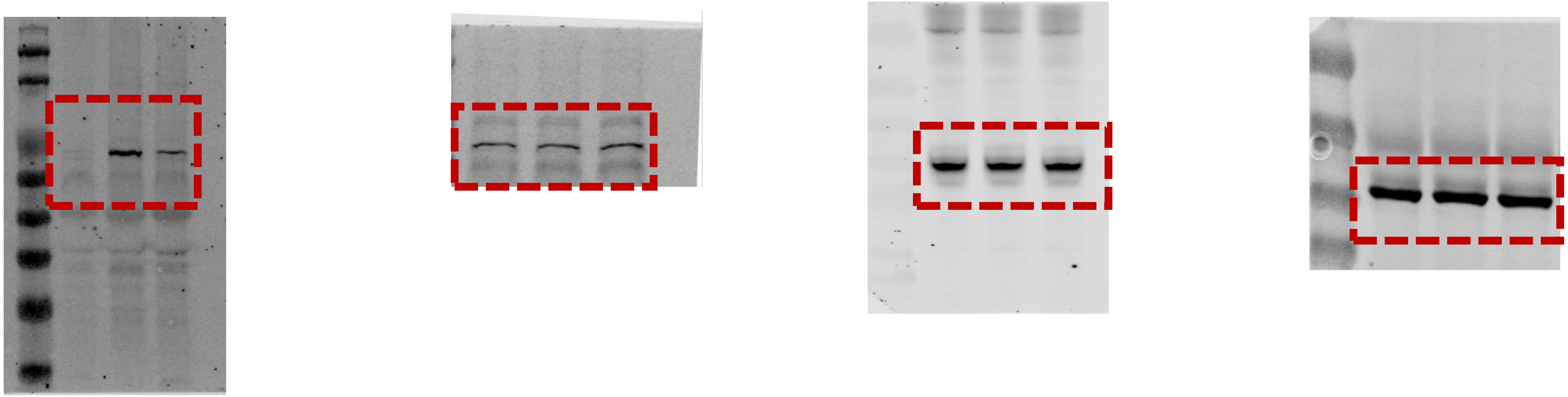

**Fig. 7K**

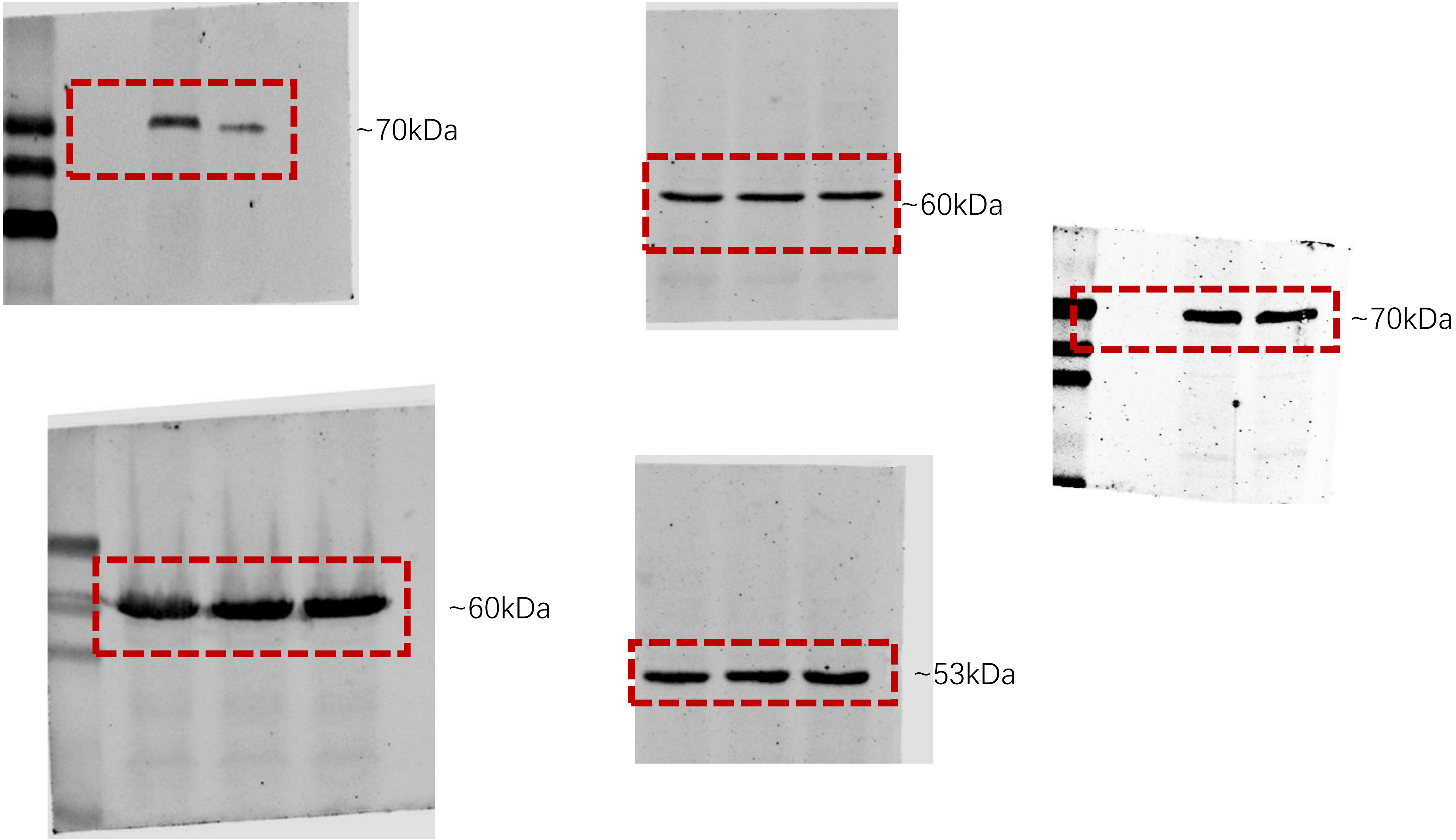

Fig. S1G

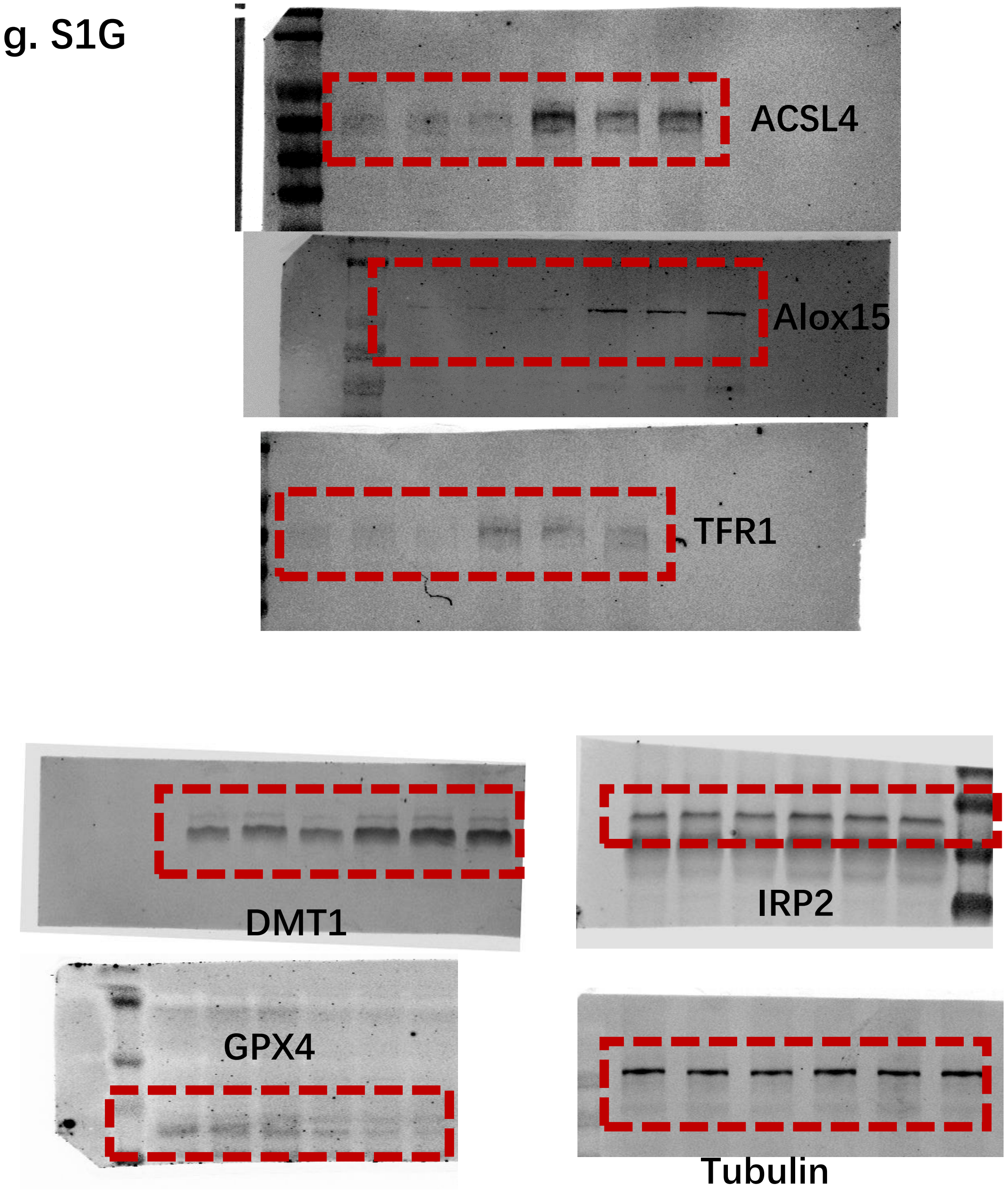


Fig. S2A

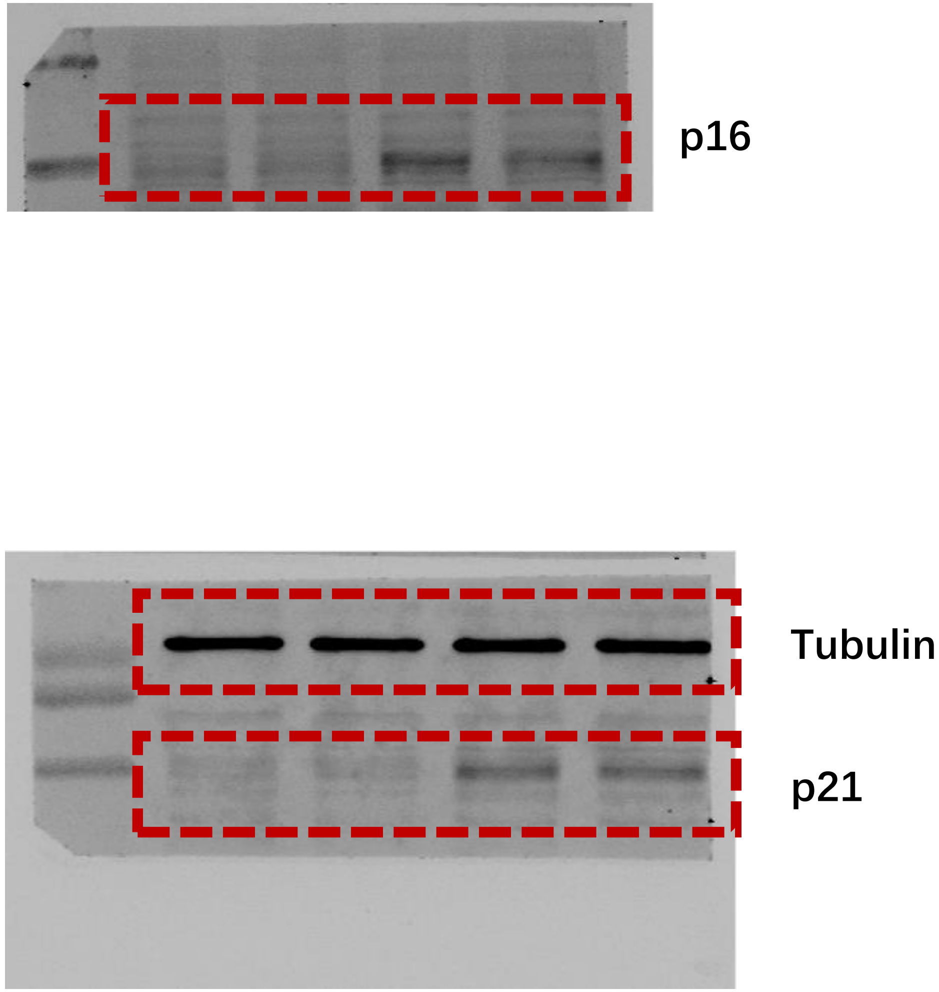

Fig. S2F

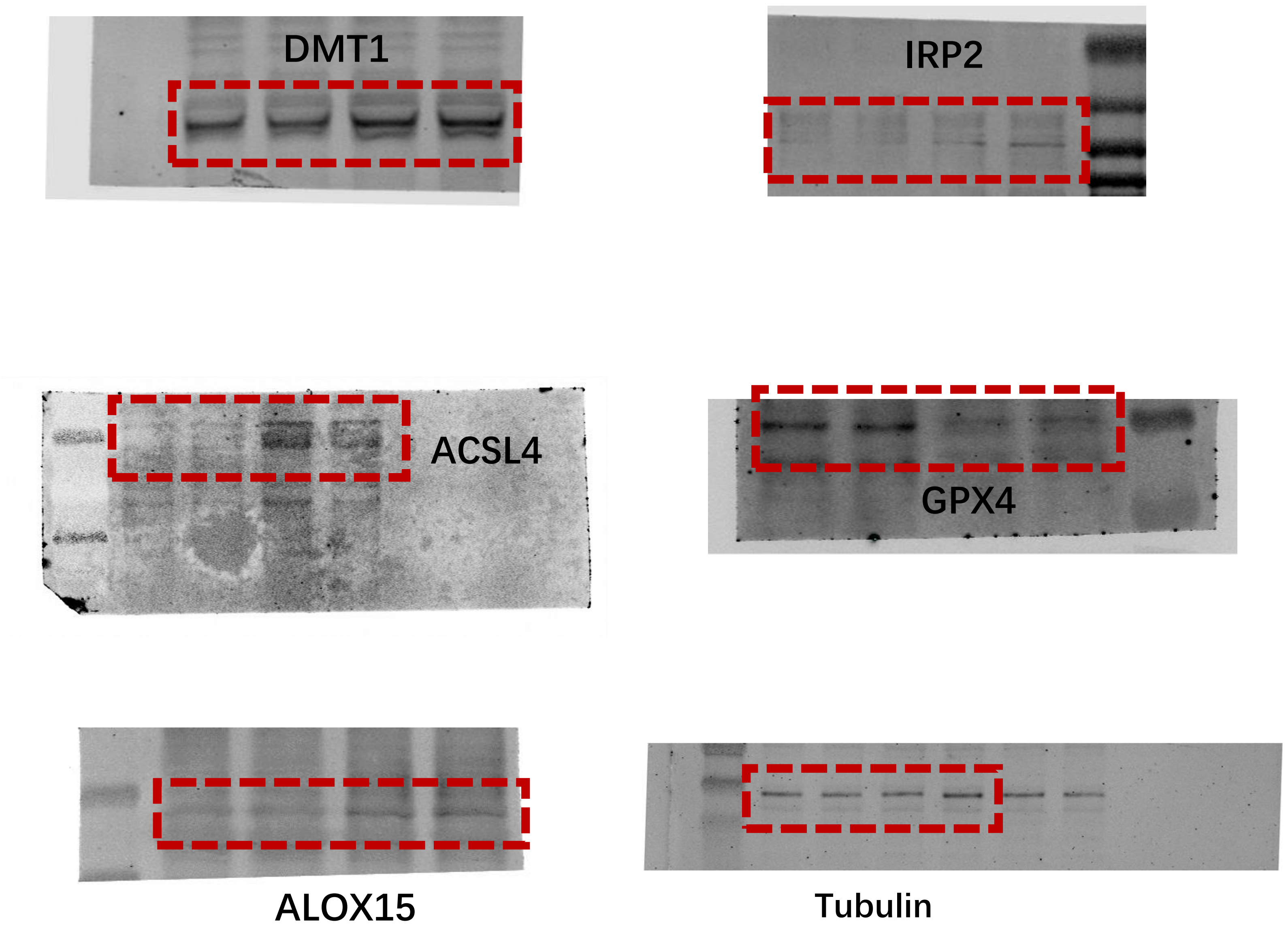


Fig. S3E

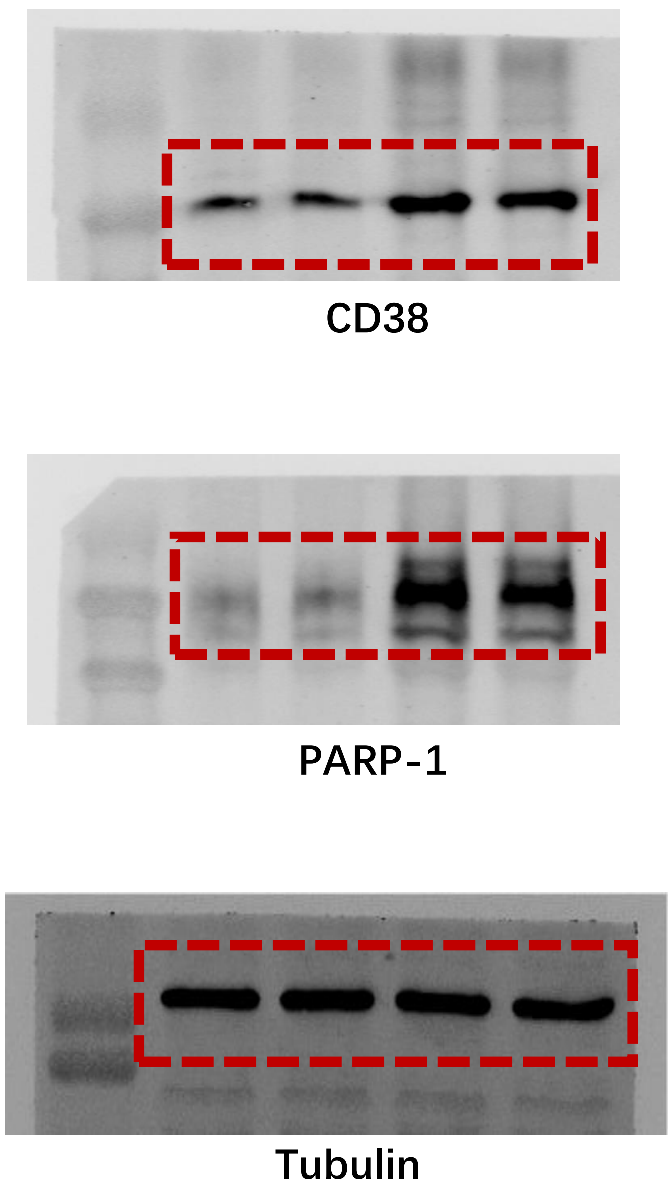

Fig. S4F

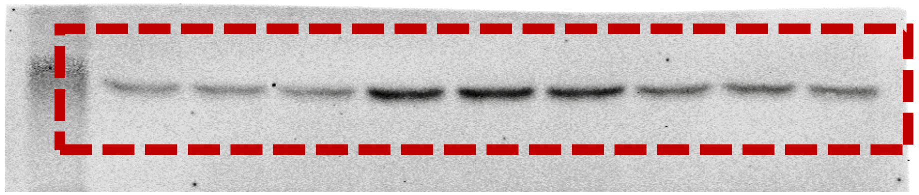

CD38

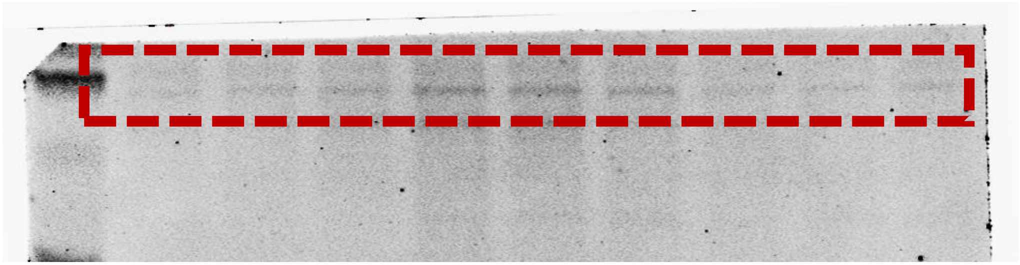

PARP-1

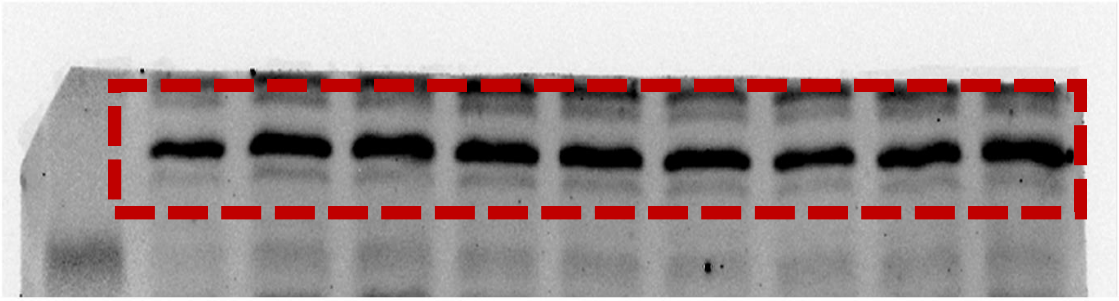

Tubulin

Fig. S4G

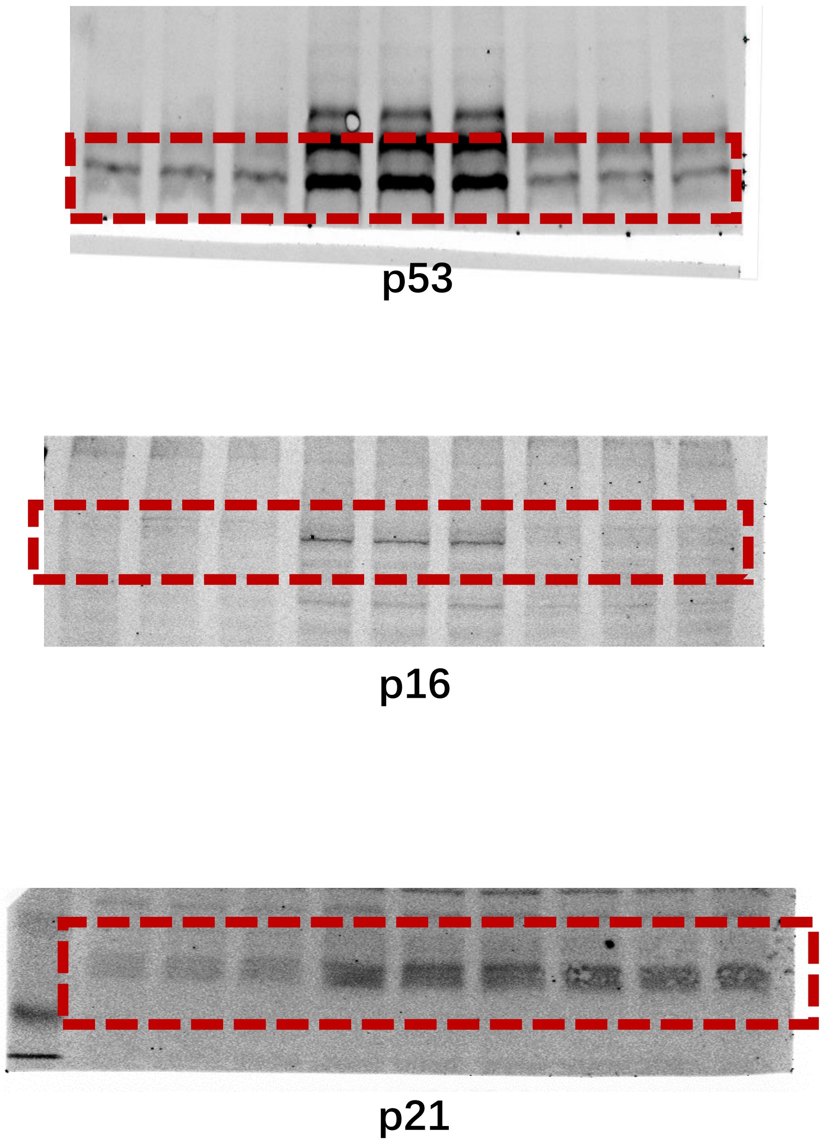


Fig. S4H

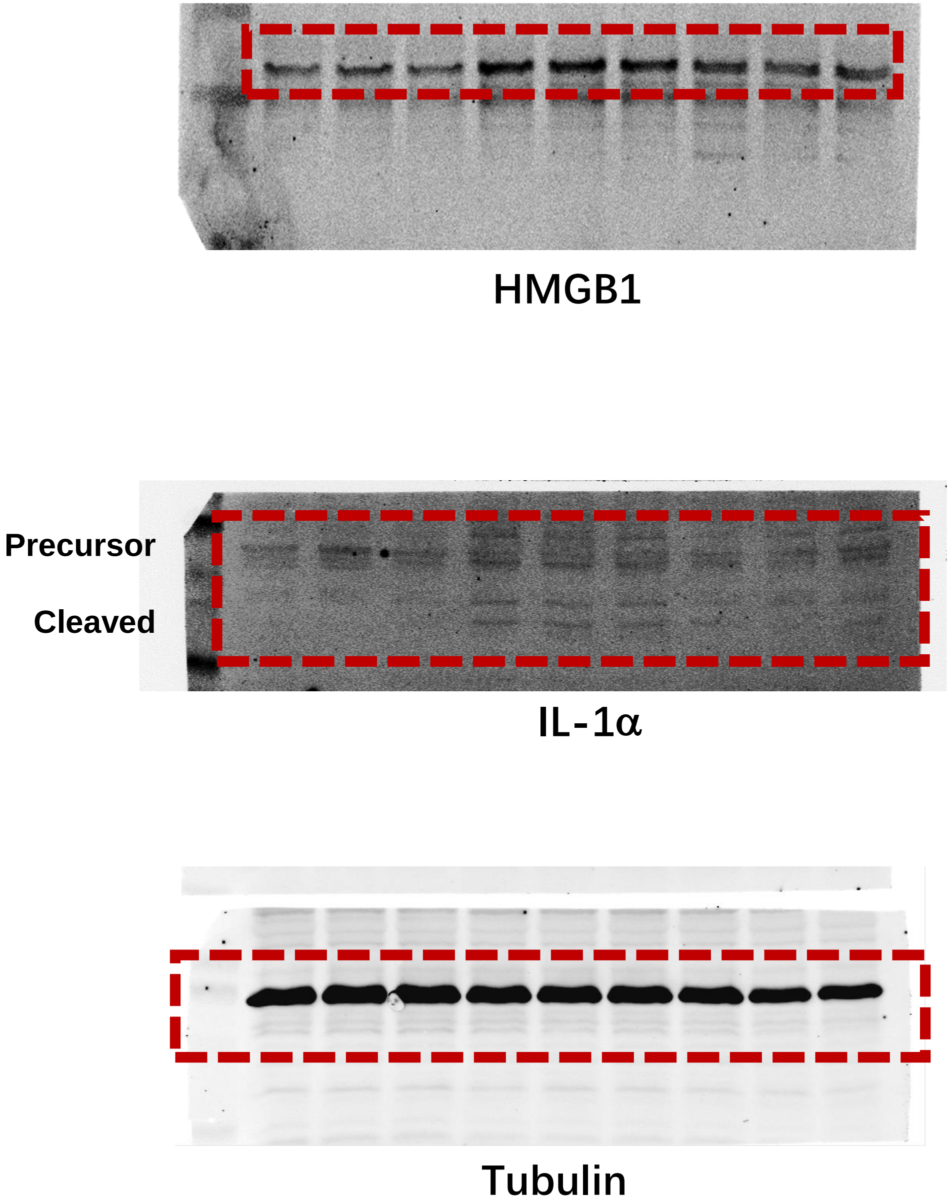

Fig. S5F

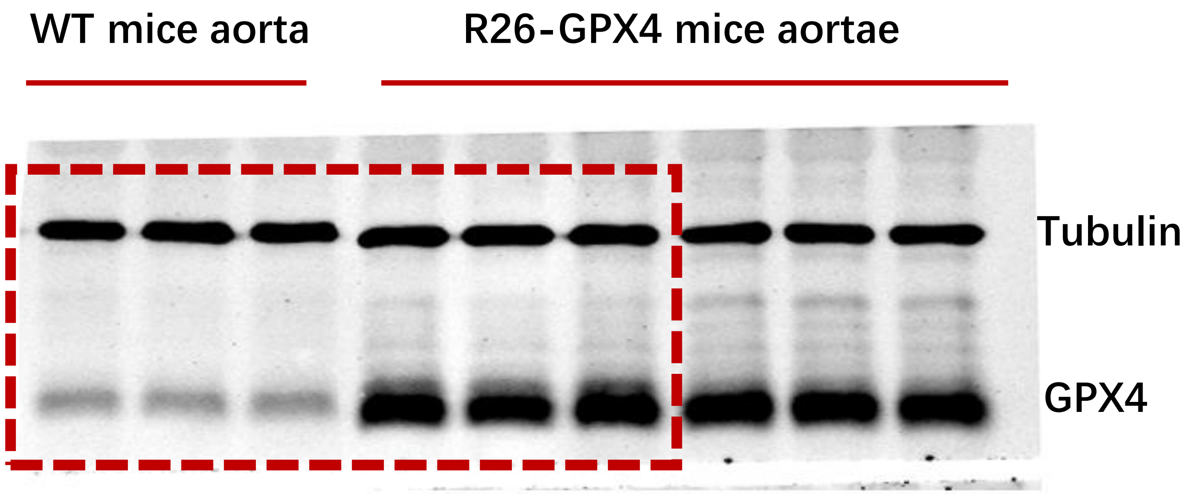

Fig. S6A

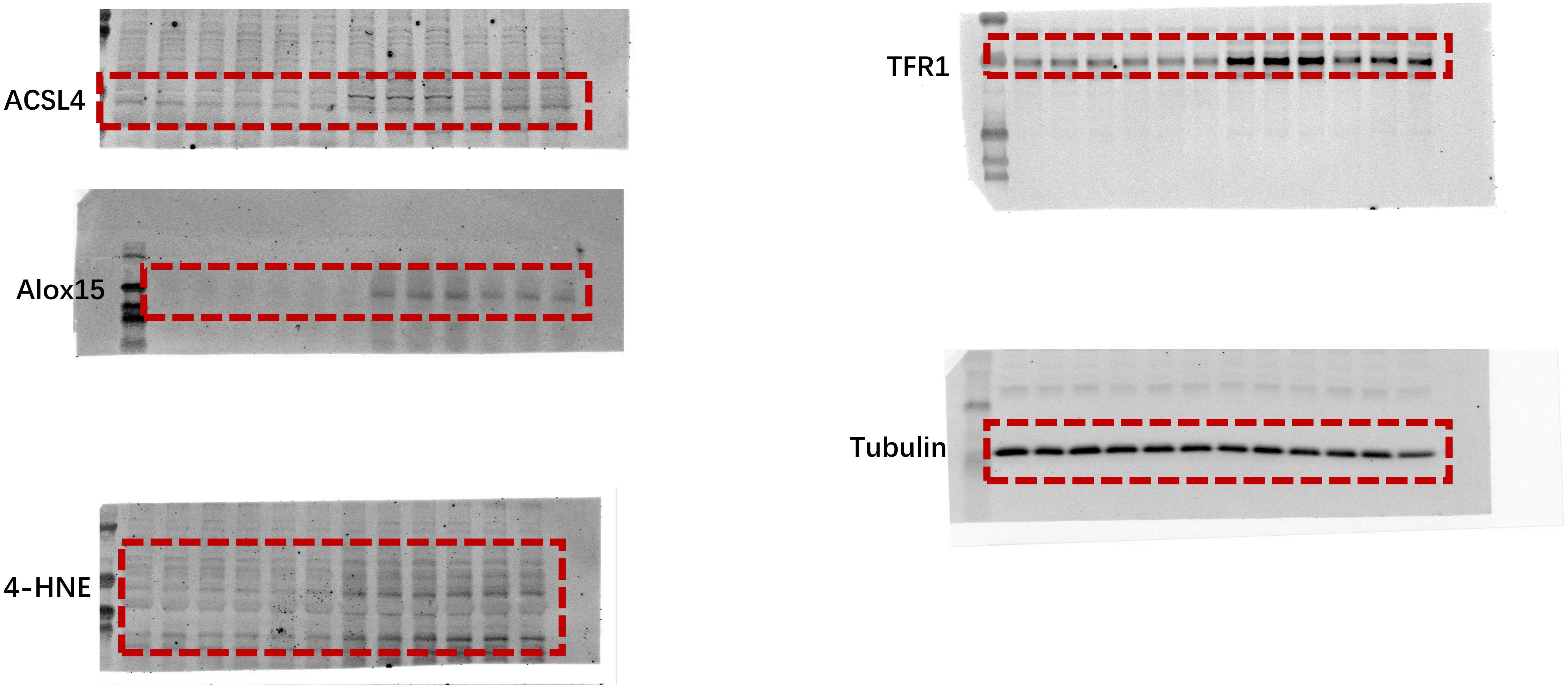

Fig. S6C

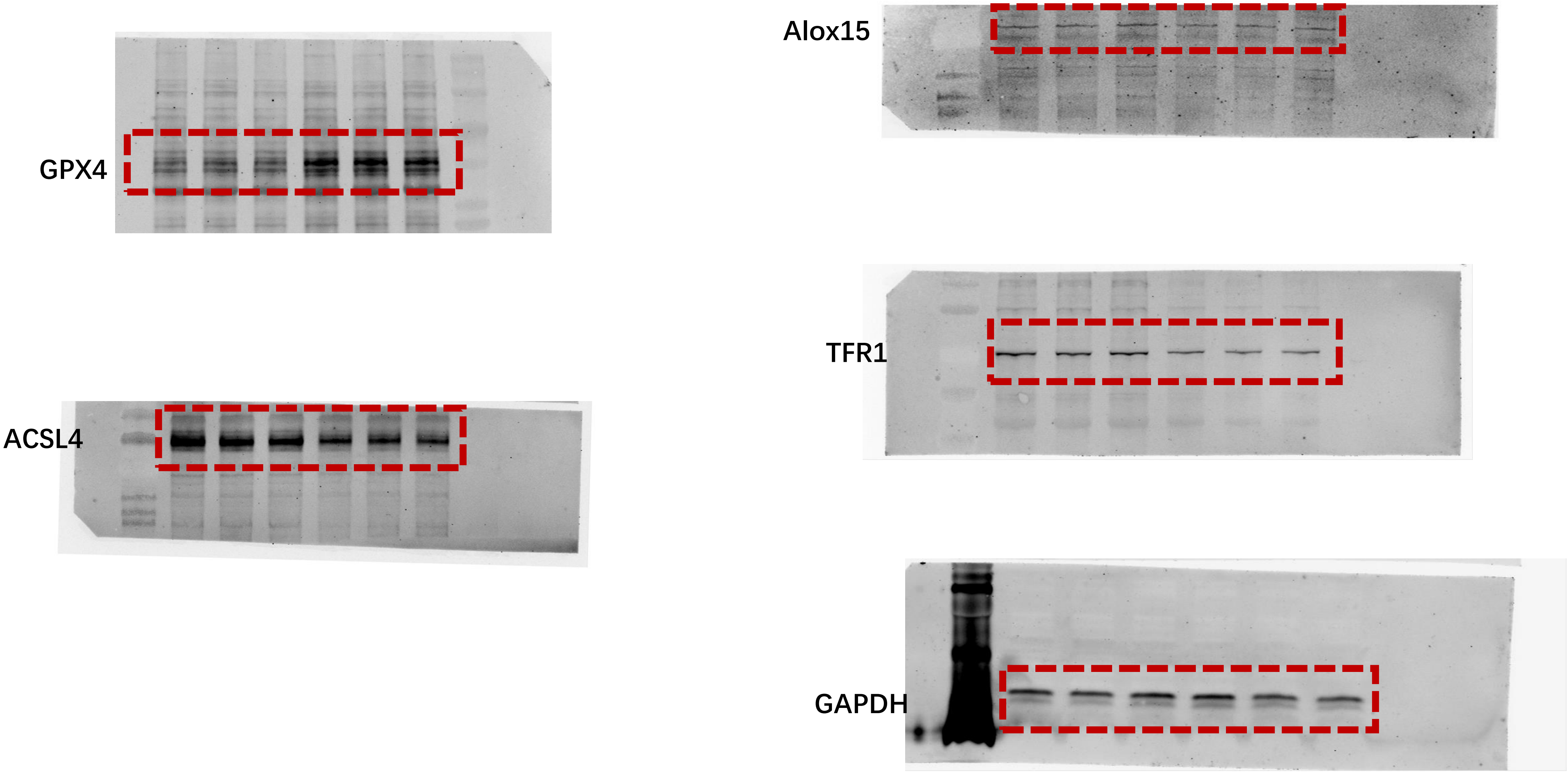

Fig. S7B

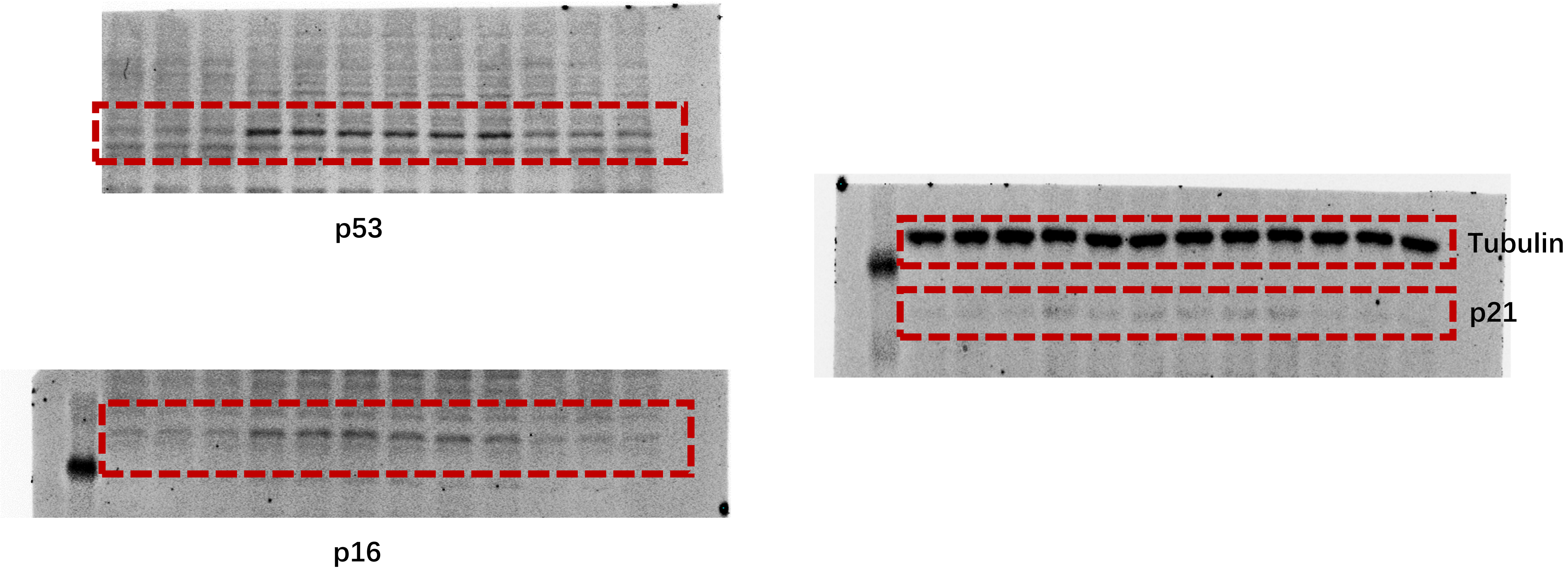

Fig. S7G

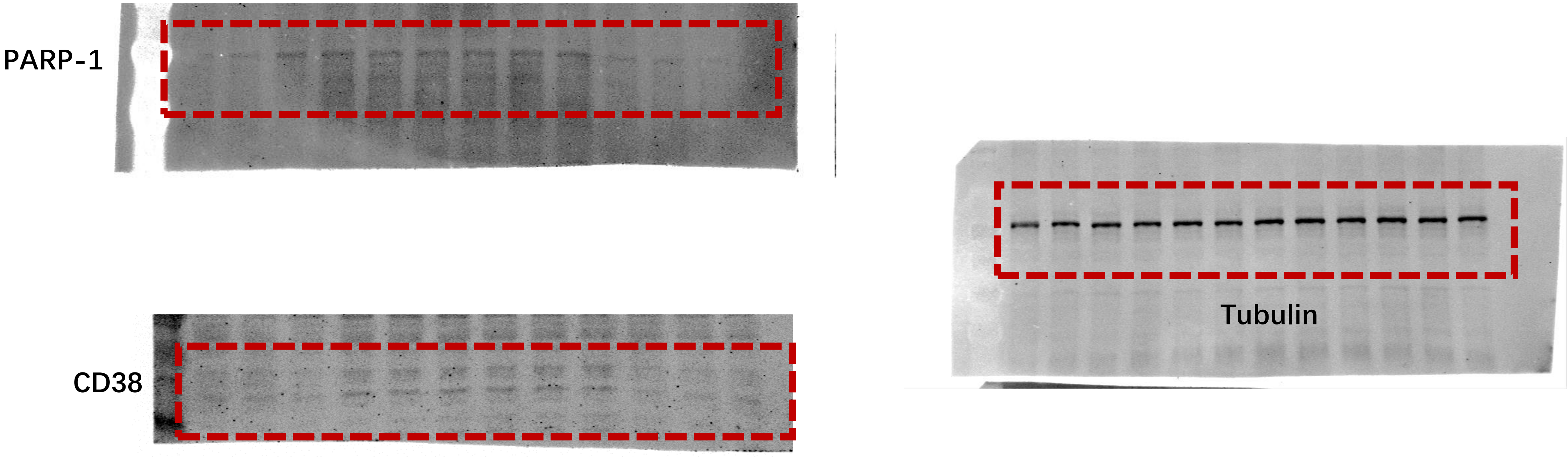

**Fig. S8C**

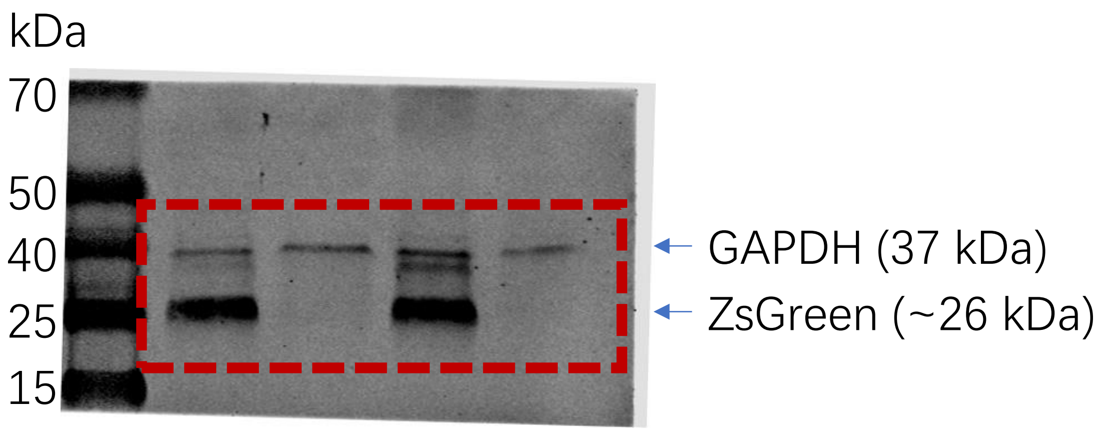


**Fig. S8D**

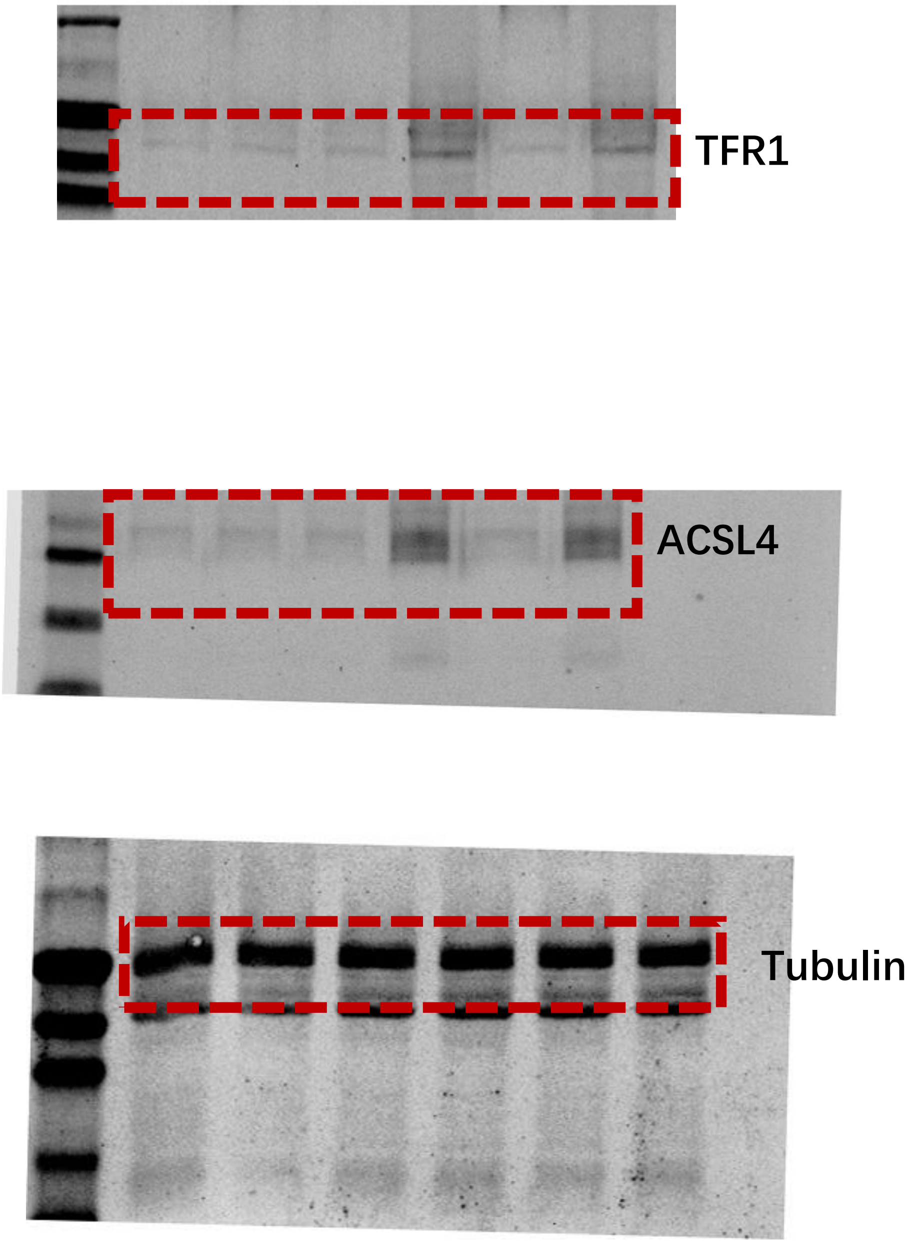

**Fig. S9B**

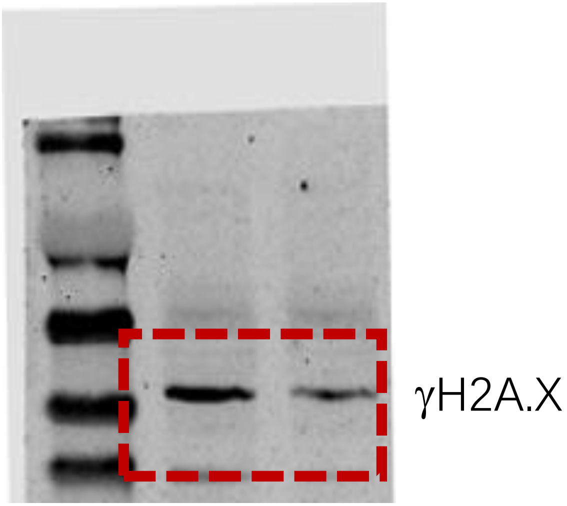


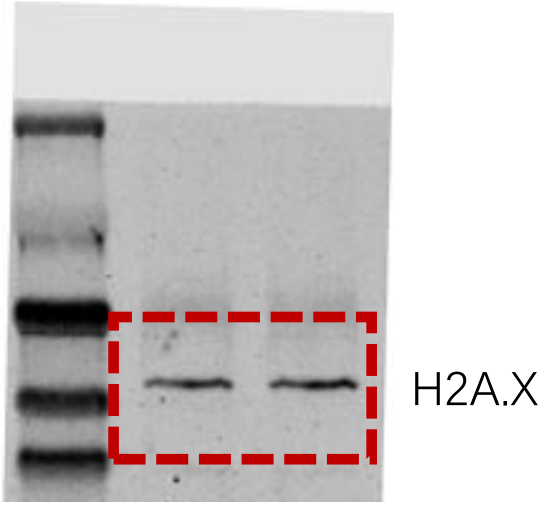

Fig. S11A

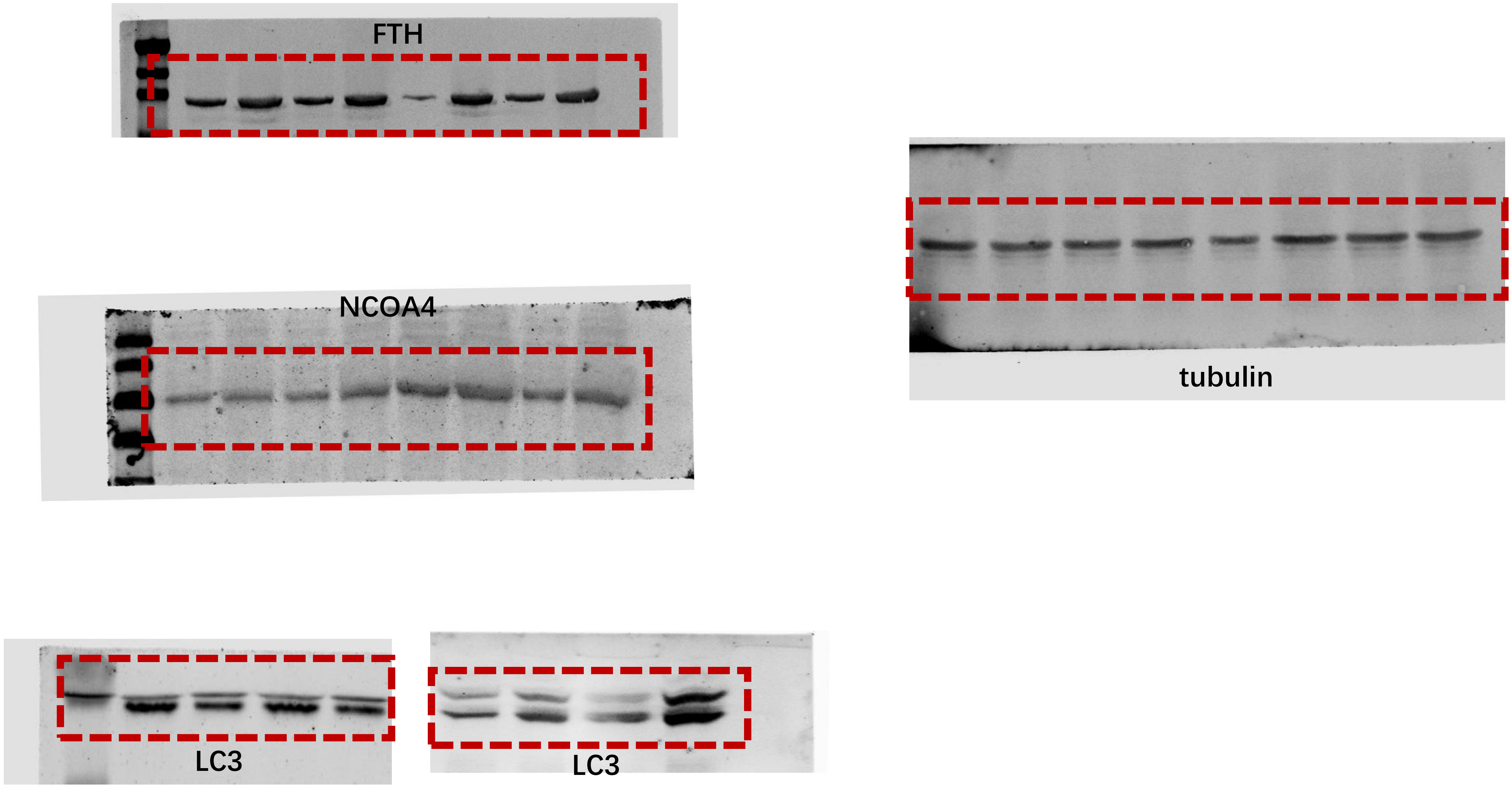

Fig. S12

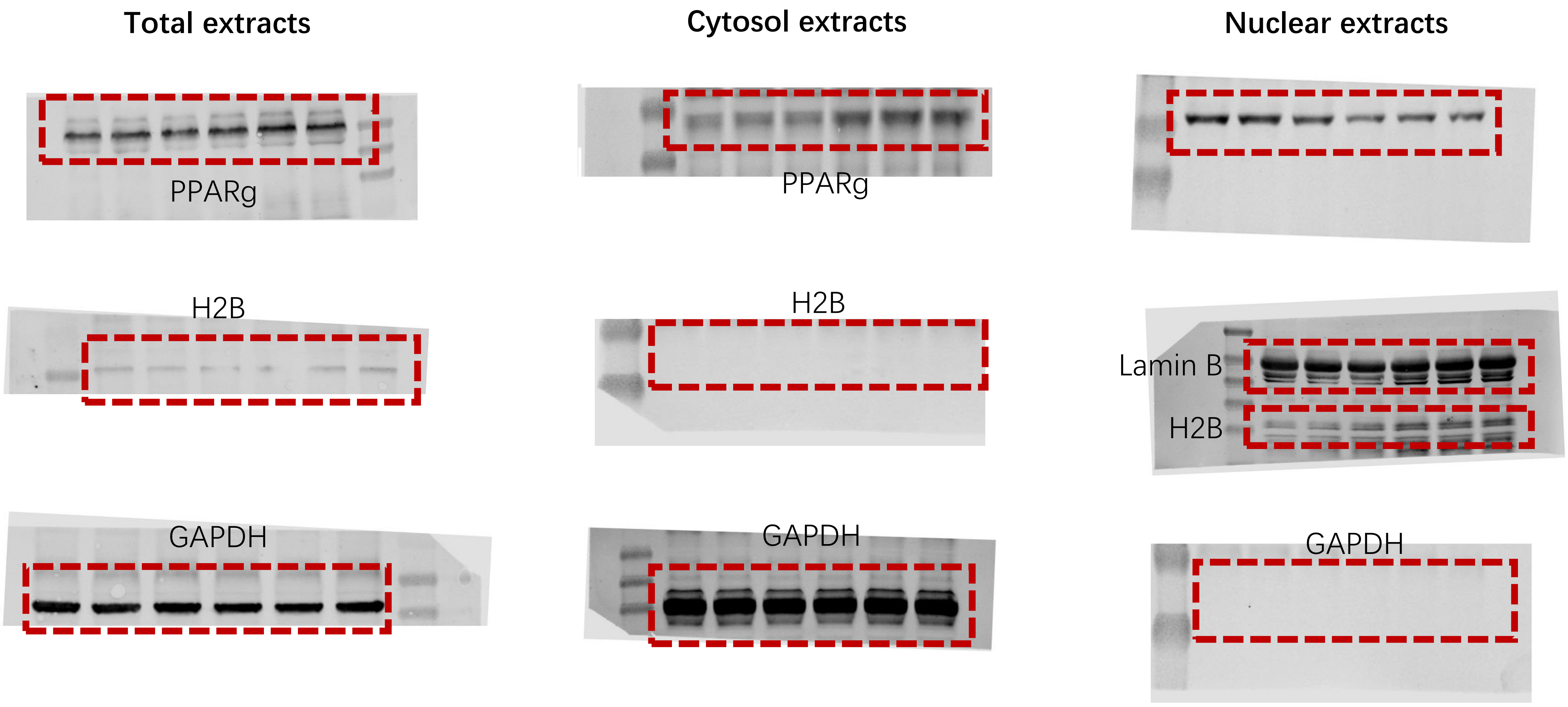
Total extracts
Cytosol extracts
Nuclear extracts
PPARg
PPARg
H2B
H2B
Lamin B
H2B
GAPDH
GAPDH
GAPDH

Fig. S13

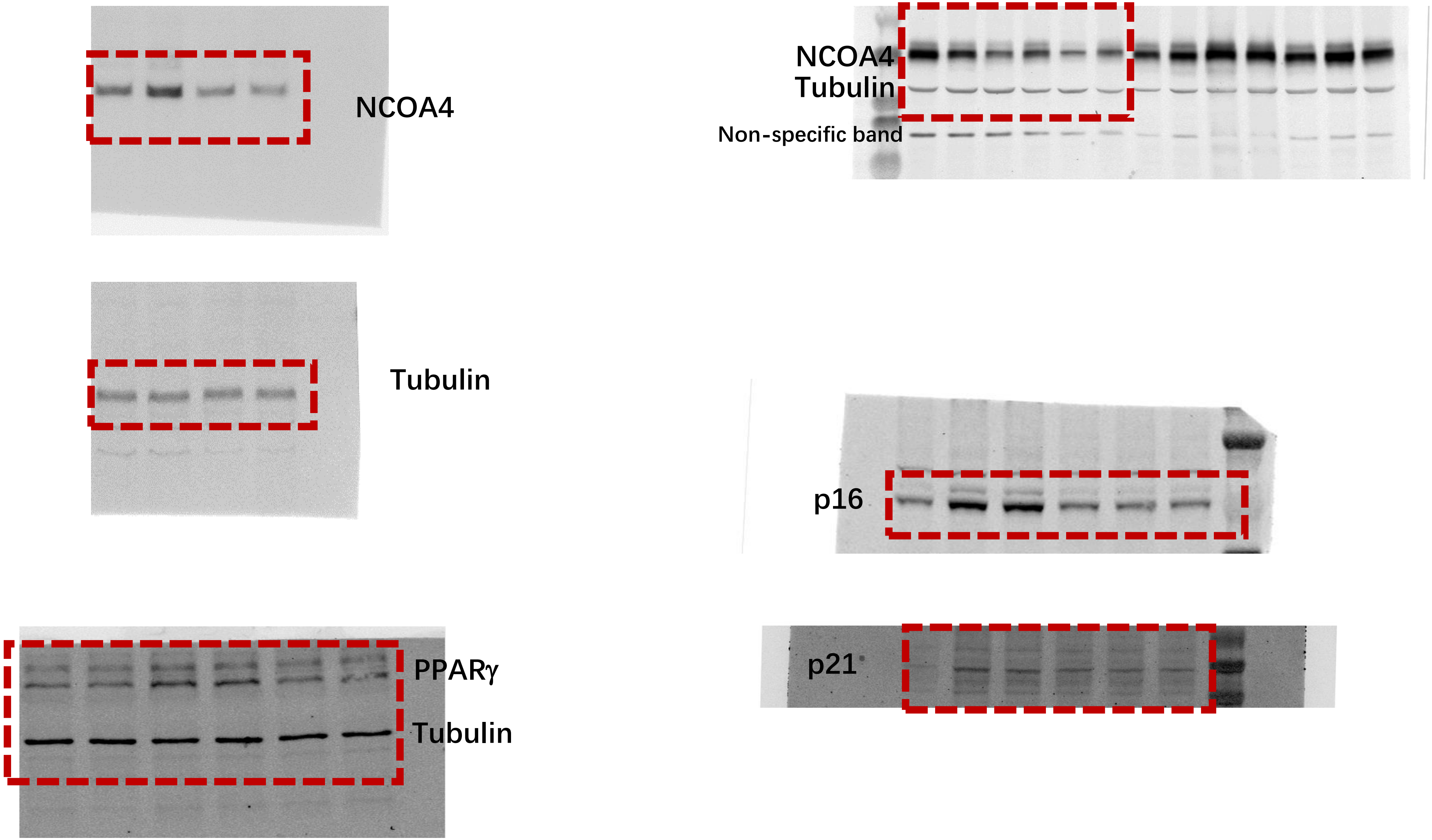
NCOA4
Tubulin
PPARγ
Tubulin
NCOA4
Tubulin
Non-specific band
p16
p21
